# High pressure assisted synthetic approach for novel 6,7-dihydro-5*H*-benzo[6,7]cyclohepta[1,2-*b*]pyridine and 5,6-dihydrobenzo[*h*]quinoline derivatives and their assessment as anticancer agents

**DOI:** 10.1038/s41598-020-78590-x

**Published:** 2020-12-10

**Authors:** Haider Behbehani, Fatemah A. Aryan, Kamal M. Dawood, Hamada Mohamed Ibrahim

**Affiliations:** 1 Chemistry Department, Faculty of Science, Kuwait University, P.O. Box 5969, 13060 Safat, Kuwait; 2 Public Authority of Applied Education and Training, College of Basic Education, Science Department, P.O. Box 23167, 13092 Safat, Kuwait; 3 Chemistry Department, Faculty of Science, Cairo University, Giza, 12613 Egypt; 4 Chemistry Department, Faculty of Science, Fayoum University, P.O. Box 63514, Fayoum, Egypt

**Keywords:** Chemical biology, Green chemistry, Medicinal chemistry

## Abstract

A novel, expedient and effective methodology for the synthesis of distinctly substituted 6,7-dihydro-5*H*-benzo[6,7]cyclohepta[1,2-*b*]pyridine and 5,6-dihydrobenzo[*h*]quinoline systems has been developed with a new synthetic platform. This process includes ammonium acetate-mediated cyclocondensation reactions of 3-oxo-2-arylhydrazonopropanals with benzosuberone and tetralone precursors, respectively, using the high-pressure Q-tube reactor, which has been found to be superior to both conventional heating and microwave irradiation. The novel protocol benefits from its high atom efficiency, economy, ease of workup, broad substrate scope and is also applicable to gram-scale synthesis. To identify and confirm the newly synthesized targeted compounds, the X-ray single-crystal as well as all possible spectroscopic methods were utilized. The cytotoxicity of the newly synthesized 6,7-dihydro-5*H*-benzo[6,7]cyclohepta[1,2-*b*]pyridine **4a–j** and 5,6-dihydrobenzo-[*h*]quinolines derivatives **6a–e** were preliminary examined toward three cell lines of human cancer; lung cancer (A549), breast cancer (MCF-7) and colon cancer (HCT-116), by applying the MTT colorimetric assay. The achieved results reflected the promising profile of the prepared compounds in this study against cancer cells and have shown that members from the synthesized 6,7-dihydro-5*H*-benzo[6,7]cyclohepta[1,2-*b*]pyridine **4a–j** exhibited promising cytotoxicity’s against MCF-7, and A549 cancer cells respectively, while the HCT-116 (colon) cancer cells were inhibited by certain examples of 5,6-dihydrobenzo[*h*]quinoline derivatives **6c,d**. These promising results could serve as a good primary base for further research into the design of anticancer drugs.

## Introduction

**Figure 1:**
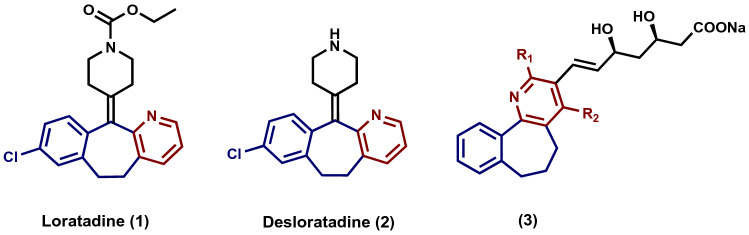
Some dihydro-benzocyclohepta[1,2-*b*]pyridine containing drugs.

The Q-tube was designed as a high-pressure tool to perform various organic reactions and transformations through an inexpensive, green, safe and environmentally benign process. Compared to conventional heating and microwave irradiation, the Q-tube has several characteristics and features^1–8^ including; better yield and performance, a cleaner product profile that means light color (less impurities and by-products), energy saver, lower reaction time and higher reproducibility, in addition to it is cheaper and safer because the sealing and pressing are easy. Such promising and unique features encouraged us to utilize the Q-tube methodology in our research to explore the impact of the high pressure on the reactions profile conducted in this study that aimed at synthesizing two very significant classes of compounds namely; 6,7-dihydro-5*H*-benzo[6,7]cyclohepta[1,2-*b*]pyridine and 5,6-dihydrobenzo[*h*]quinoline derivatives. These two classes of compounds contain a wide range of structurally significant substances, which exhibit numerous medicinally- and pharmaceutically-relevant characteristics and behaviors, For instance, 6,7-dihydro-5*H*-benzo[6,7]cyclohepta[1,2-*b*]pyridine derivatives have demonstrated a number of potent applications and activities, such as anti-cancer^9,10^, anti-inflammatory^11–13^, anti-tumor^14–16^, anti-tuberculosis^17^, anti-HIV^12^, anti-HCV^18^, antioxidant^18^, anti-Alzheimer^19^, as well as anti-histamine^20–24^. Loratadine (**1**) and Desloratadine (**2**) (Fig. 1), for example, are two commercial drugs used for treatment allergies, including allergic rhinitis, nasal congestion and hives. Moreover, 6,7-dihydro-5*H*-benzo[6,7]cyclohepta[1,2-*b*]pyridine systems are important pharmacophores for the development of drug design, for example 6,7-dihydro-5*H*-benzo[6,7]cyclohepta[1,2-*b*]pyridine derivatives **3** (Fig. 1) were reported as patents for the treatment of hypertriglyceridemia, hypercholesterolemia, hyperlipidemia and dyslipidemia^25,26^. They are also used as selective and potent human neurokinin-3 receptor antagonists (HNK-3)^27^. Furthermore, synthetic and drug chemists are attracted by functionalized dihydrobenzo[*h*]quinolines because they are possess some pharmacological activities, including, but not limited to, anti-tumor^28^, anti-cancer^29^, and anti-bacterial activity^30^. As a result of these elegant applications and features and in the continuation of our efforts to develop novel protocols for synthesizing *N*-containing heterocycles^31–42^, 6,7-dihydro-5*H*-benzo[6,7]cyclohepta[1,2-*b*]pyridine and 5,6-dihydrobenzo[*h*]quinoline have attracted our attention. After careful literature survey, several methods have been developed to synthesize these two classes of compounds^16–18,43,44^, but to the best of our knowledge, this is the first route that includes the 3-oxo-2-arylhydrazonopropanals as starting material and the Q-tube reactor. The new methodological approach involves [4 + 2] ammonium acetate-mediated cyclocondensation process between 3-oxohydrazonopropanals with benzosuberone and tetralone precursors, using the Q-tube high-pressure reactor. In this synthesis, the Q-tube pressure reactor was found to be superior to both microwave irradiation and conventional heating. The significant advantages of the novel process are that the reactants are common, easy workup and have a broad substrate scope, high atom economy and efficiency. The compounds prepared in this research were evaluated as anti-cancer agents.

## Results and discussion

**Scheme 1:**
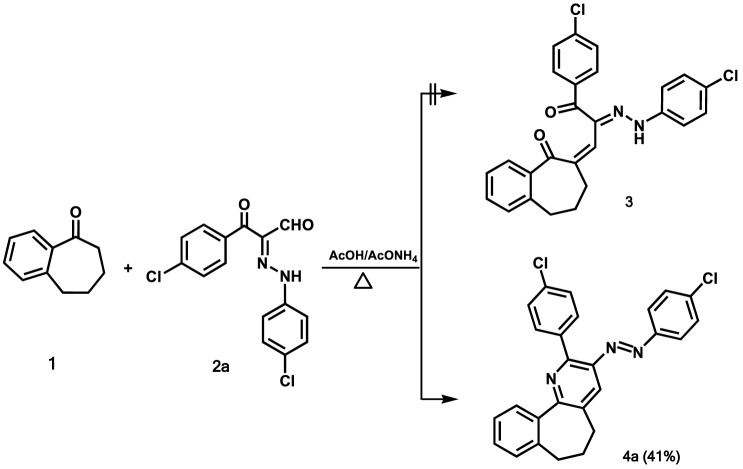
Reactions of benzosuberone **1** and arylhydrazonal **2a**.

**Figure 2:**
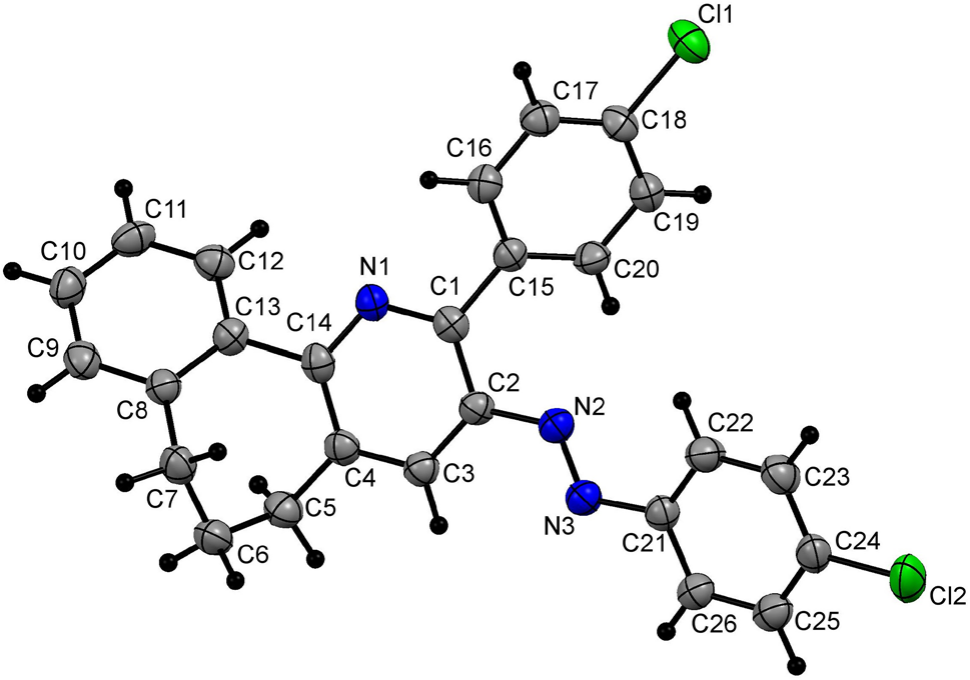
X-ray plot of single crystallographic data collected for **4a**. Mercury (version 3.8) (https://www.ccdc.cam.ac.uk/solutions/csd-system/components/mercury/) was used to create this figure.

In this context, our research aimed to establish a new greener approach focused on the use of high pressure to improve energy efficiency and, as shown latter by the green metrics, this methodology had good records, such as high atom economy, for the synthesis of polysubstituted 6,7-dihydro-5*H*-benzo[6,7]cyclohepta[1,2-*b*]pyridine. This methodology was initiated by exploring the reaction between the benzosuberone **(1)** and 3-(4-chlorophenyl)-2-[2-(4-chlorophenyl)hydrazono]-3-oxopropanal (**2a)** as a model reaction (Table 1). Initially, it was observed that stirring a mixture of benzosuberone (**1**) (5 mmol) and arylhydrazonal **2a** (5 mmol) in various solvents, such as polar aprotic (DMF, dioxane and CH_3_CN), polar protic (ethanol and propanol) (15 ml) containing ammonium acetate (10 mmol) at reflux under normal pressure for 12 h, did not configure any product (entries 1–5, Table 1). On the other hand, carrying out the same reaction in acetic acid (10 ml) as a solvent at reflux for 4 h resulted in the formation of a product in 41% yield (Table 1, entry 6). This product has been assigned to be (4-chlorophenyl)-{2-(4-chlorophenyl)-6,7-dihydro-5*H*-benzo[6,7]cyclohepta[1,2-*b*]pyridin-3-yl}diazene (**4a**) and not the non-cyclized product **3** based on various spectroscopic data (Scheme 1). For instance, the mass and high-resolution mass spectrometric analyses of **4a** revealed that it had a mass of m/z 443 and an exact mass of m/z 443.0950 for the corresponding molecular composition of C_26_H_19_Cl_2_N_3_. The ^1^H NMR spectrum of **4a** in CDCl_3_ showed a set of resonances in the region of 7.31–7.93 ppm due to twelve aromatic protons, a singlet signal at 7.99 ppm for pyridine H-4, in addition to the set of peaks associated with 6 C*H*_2_ protons in the aliphatic region as quintet (2.34 ppm) and multiplet (2.65–2.69 ppm) respectively. Additionally, the ^13^C{^1^H} NMR spectra of **4a** includes 22 signals as expected and devoid of any carbonyl signals. These data were undoubtedly refined and supported by acquisition the single crystallographic data for compound **4a** (Fig. 2; Table 2), which confirmed the assigned structure and demonstrated that only the (*E*)-isomer of (4-chlorophenyl)-[2-(4-chlorophenyl)-6,7-dihydro-5*H*-benzo[6,7]cyclohepta[1,2-*b*]pyridin-3-yl]diazene was isolated and formed.

**Table 1:** Optimized reaction condition between benzosuberone **1** and arylhydrazonal **2a**.

Entry	Solvent	Temp (°C)	Time	Yield (%)
1	DMF	Reflux	12 h	**–**
2	1,4-dioxane	Reflux	12 h	**–**
3	CH_3_CN	Reflux	12 h	**–**
4	EtOH	Reflux	12 h	**–**
5	propanol	Reflux	12 h	**–**
6	AcOH	Reflux	4 h	41^a^
7	AcOH	MW (sealed tube) (120 °C, 250 W)	25 min	63^b^
8	AcOH	MW (sealed tube) (130 or 150 °C, 250 W)	30 min	69^b^
9	AcOH	Q-tube (150 °C)	30 min	85^c^
10	AcOH	Q-tube (155 °C)	30 min	91^c^
11	AcOH	Q-tube (160 °C)	30 min	95^c^
**12**	**AcOH**	**Q-tube (165 °C)**	**30 min**	**98**^c^
13	AcOH	Q-tube (170 °C)	30 min	98^c^

Reaction conditions: ^a^A mixture of benzosuberone (**1**) (5 mmol), 3-oxoarylhydrazonal **2a** (5 mmol) and ammonium acetate (10 mmol) in acetic acid (10 ml) was refluxed under normal pressure for 4 h.

^b^A mixture of benzosuberone (**1**) (2 mmol), 3-oxoarylhydrazonal **2a** (2 mmol) and ammonium acetate (4 mmol) in acetic acid (5 ml) was introduced to the microwave tube (10 ml) and irradiated by MW (250 W) at the reported temperature and time.

^c^A mixture of benzosuberone (**1**) (5 mmol), 3-oxoarylhydrazonal **2a** (5 mmol) and ammonium acetate (10 mmol) in acetic acid (10 ml) was introduced to the Q-tube (35 ml) and heated in an oil bath at the reported temperatures for 30 min.

**Table 2:** Some of the selected bond angles and bond lengths for **4a**.

Bond	Bond length(Å)	Bond	Bond angle(°)
C9–C10	1.384(5)	C6–C7–C8	113.8(3)
C6–C7	1.534(5)	C5–C6–C7	112.2(3)
C5–C6	1.524(4)	C1–N1–C14	118.6(3)
C7–C8	1.507(5)	C2–C3–C4	120.7(3)
N2–C2	1.418(3)	N2–C2–C3	124.1(3)
N3–C21	1.429(3)	N3–N2–C2	115.0(3)
N2–N3	1.261(4)	N2–N3–C21	112.5(3)
N1–C1	1.344(3)	N1–C1–C15	116.4(3)
Cl2–C24	1.734(3)	N1–C14–C13	116.7(3)

This result encouraged us to study the optimization factors that influence this reaction in order to synthesize these targeted compounds in a sustainable and green manner, and to conduct a comparative study between the Q-tube pressure reactor as an affordable, economical alternative to the expensive microwave (MW) that allows a certain chemical reaction to be carried out safely at high pressure due to the easy sealing and pressure relief feature, which prevents the accidental explosions due to increased pressure during the use of the traditional sealed pressure tube. Thus, for the purposes of comparison, initially we conducted the above-mentioned reaction in MW by mixing benzosuberone (**1**) (2 mmol), arylhydrazonal **2a** (2 mmol) and NH_4_OAc (4 mmol) in AcOH (5 ml) in a 10 ml MW tube (sealed tube) which irradiated at 125 °C (25 min, 250 W), resulting in the formation of compound **4a** in 63% yield (Table 1, entry 7). On the other hand, the same reaction is carried out on a larger scale [benzosuberone (**1**) (5 mmol), arylhydrazonal **2a** (5 mmol) and NH_4_OAc (10 mmol) in AcOH (10 ml)] using the Q-tube pressure-reactor tube (35 ml) and after heating at 150 °C for 30 min leading to the formation of the same product **4a** at a higher yield 85% (Table 1, entry 9). It is worth mentioned that doubling the reactant quantities also provided **4a** in a very comparable yield, and it is also noted that longer reaction time does not improve the reaction yield. The noticeable increase in the yield by using the MW and Q-tube reactor can be attributed to the fact that the reaction was performed at an exceptionally high pressure and at a higher temperature than the b.p. of the reagents and solvents, and according to the Arrhenius concept, the rate of the reaction increased as the temperature increased (high b.p.)^45,46^. Additionally, the increased pressure reduces the volume of the reaction, which increases the concentration and collision frequency, thus increasing the reaction rate (speeds up a reaction). Furthermore, the competing degradation of reagents, reactants and even products can be minimized when the Q-tube (uniform heating) was used to conduct reaction since the microwave energy is known to cause localized overheat (hot spots) which could lead to decomposition reactions and formation of by-products specially in heterocyclic synthesis, so using the Q-tube enabling a cleaner pattern of reaction to arise (clean and higher product yields). After the clear emphasis on the efficiency of Q-tube and acetic acid-ammonium acetate buffer system to implement the targeted reaction (Table 1, entry 9), we then proceed to study the effect of the temperature on the reaction course. It is also clear that the temperature has a significant role on the reaction efficacy, so that when the reaction was performed at 155 °C, the reaction yield is found to be 91%, (Table 1, entry 10) whereas when the temperature increases to 160, 165, then 170 °C, **4a** was formed in 95%, 98% and 98% yield respectively, so that the best temperature for this reaction is 165 °C (entry 12, Table 1).

**Figure 3:**
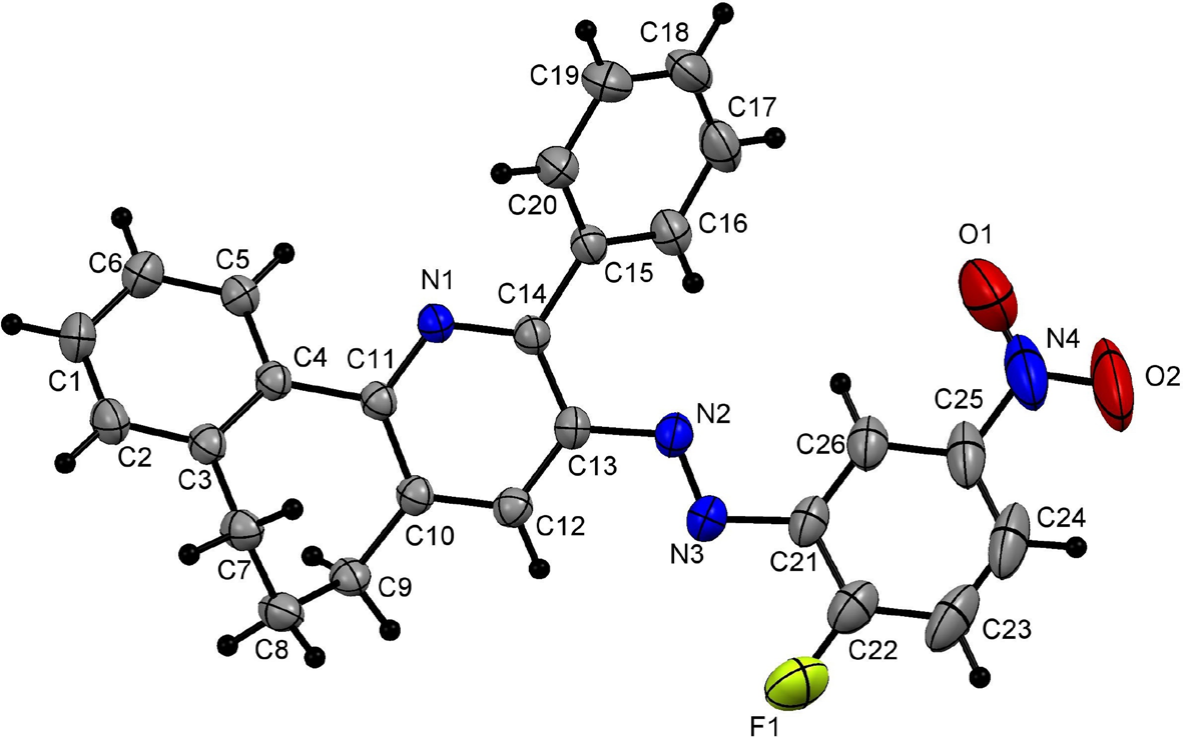
X-ray plot of the crystallographic data collected for **4b**. Mercury (version 3.8) (https://www.ccdc.cam.ac.uk/solutions/csd-system/components/mercury/) was used to create this figure.

**Figure 4:**
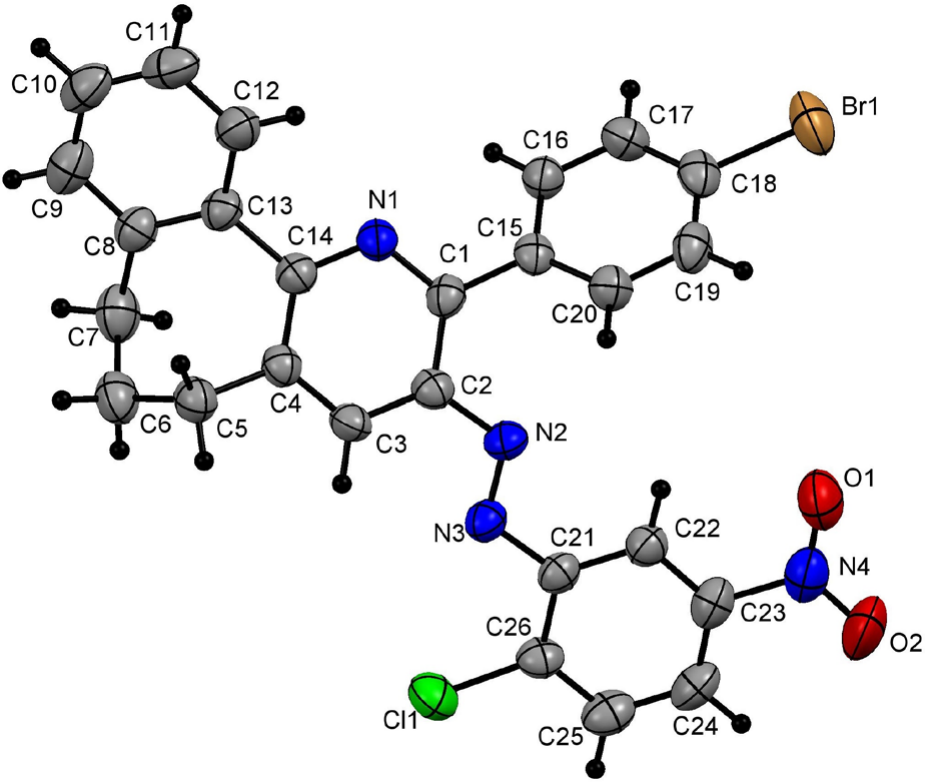
X-ray plot of the crystallographic data collected for **4d**. Mercury (version 3.8) (https://www.ccdc.cam.ac.uk/solutions/csd-system/components/mercury/) was used to create this figure.

Following the development of optimized reaction conditions for the new 6,7-dihydro-5*H*-benzo[6,7]cyclohepta[1,2-*b*]pyridine **4a** forming process (Entry 12, Table 1), a further study was conducted to investigate its substrate scope. For this purpose, a series of 3-oxo-2-arylhydrazonopropanals **2b–j** containing an assortment aryl substituted moieties, like phenyl, phenyl-substituted with electron donating group, phenyl-substituted with one or two electron withdrawing groups, or heteroaryl. Table 3 showed that reactions between benzosuberone **1** and **2b–j** take place in the same sequences and lead to the formation of the corresponding 6,7-dihydro-5*H*-benzo[6,7]cyclohepta[1,2-*b*]pyridine **4b–j** at a comparable high-yield (Table 3) and, consequently, that the number or the nature of substituents on both aryl moieties in the arylhydrazonopropanal precursor either electron-withdrawing or -donating has no effect on the efficacy of the new developed high pressure-assisted cyclocondensation process. Furthermore, members from the newly-prepared 6,7-dihydro-5*H*-benzo[6,7]cyclohepta[1,2-*b*]pyridine family were proven and authenticated by acquiring their X-ray single crystallographic data such as **4b,d** as shown in Figs. 3, 4, all of which indicate that only the (*E*)-isomer was isolated and formed.

**Table 3:** Cyclocondensation reactions between benzosuberone (**1**) and arylhydrazonals **2a–j** using Q-tube.^a^

Entry	Reactants	Ar_1_	Ar_2_	Product	Yield (%)
**1**	**1 + 2a**	4-Cl-Ph	4-Cl-Ph		**98**
**2**	**1 + 2b**	Ph	2-F-5-NO_2_-Ph		**94**
**3**	**1+ 2c**	4-Cl-Ph	2-Cl-5-NO_2_-Ph		**99**
**4**	**1 + 2d**	4-Br-Ph	2-Cl-5-NO_2_-Ph		**96**
**5**	**1 + 2e**	4-Cl-Ph	2-F-5-NO_2_-Ph		**97**
**6**	**1 + 2f**	4-NO_2_-Ph	2-F-5-NO_2_-Ph		**95**
**7**	**1 + 2g**	4-Br-Ph	2-F-5-NO_2_-Ph		**96**
**8**	**1 + 2h**	4-Br-Ph	2,4-diF-Ph		**99**
**9**	**1+ 2i**	4-MeO-Ph	2-F-5-NO_2_-Ph		**91**
**10**	**1 + 2j**	C_4_H_3_S	2-F-5-NO_2_-Ph		**93**

^a^Reaction conditions: a mixture of benzosuberone (**1**) (5 mmol), arylhydrazonopropanal **2a–j** (5 mmol), and NH_4_OAc (10 mmol) in AcOH (10 ml) was charged in the Q-tube reactor's 35 ml glass tube and heated for 30 min at 165 °C (oil bath).

**Figure 5:**
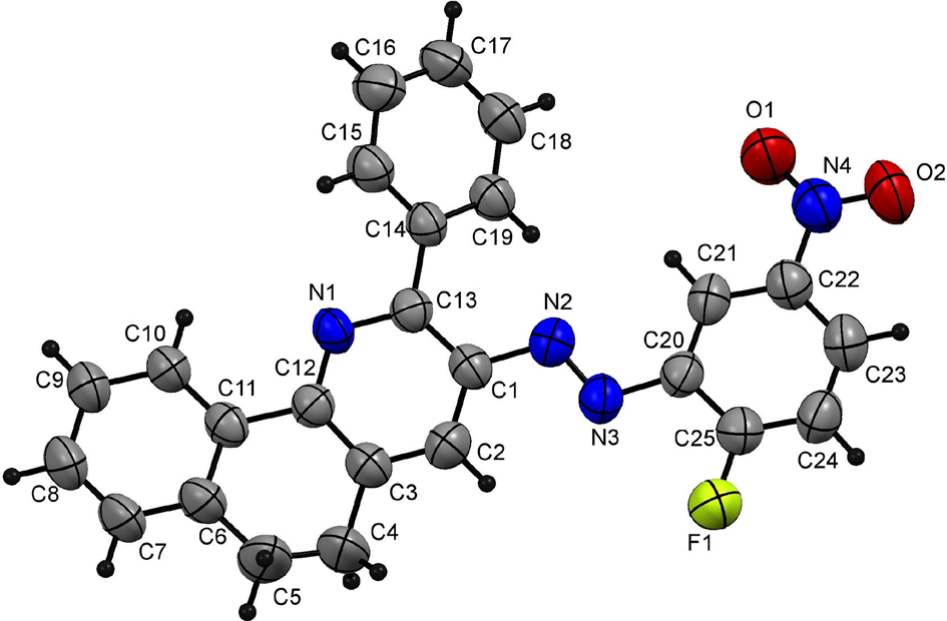
X-ray plot of single crystallographic data collected for **6b**. Mercury (version 3.8) (https://www.ccdc.cam.ac.uk/solutions/csd-system/components/mercury/) was used to create this figure.

Furthermore, the proposed strategy could also be applied successfully to tetralone. Thus, reacting tetralone (**5**) (5 mmol), arylhydrazonals **2a–d, k** (5 mmol) in the presence of NH_4_OAc (10 mmol) and AcOH (10 ml) as a solvent using the high pressure Q-tube reactor at 165 °C for 30 min, producing the unreported 5,6-dihydrobenzo[*h*]quinoline derivatives **6a–e** in excellent yields (Table 4). It is worth noting that, when using the 3-oxo-2-arylhydrazonopropanal **2k** containing the aliphatic methyl moiety, the quinoline derivative **6e** was formed at a lower yield (85%) compared to the arylhydrazonal derivatives **2a–d** containing phenyl or aryl moiety substituted with electron-deficient group, which yield the respective products **6a–d** in excellent yields (94–98%) (Table 4). One member of this family **6b** (Table 5) was also authenticated by the acquisition of its X-ray single crystallographic data which proves (*E*)-isomerization as shown in Fig. 5.

**Table 4:** Cyclocondensation reactions between tetralone (**5**) and arylhydrazonals **2a–d, k** using Q-tube.^a^

Entry	Reactants	Ar_1_	Ar_2_	Product	Yield (%)
**1**	**5 + 2a**	4-Cl-Ph	4-Cl-Ph		**94**
**2**	**5 + 2b**	Ph	2-F-5-NO_2_-Ph		**95**
**3**	**5+ 2c**	4-Cl-Ph	2-Cl-5-NO_2_-Ph		**98**
**4**	**1 + 2d**	4-Br-Ph	2-Cl-5-NO_2_-Ph		**97**
**5**	**5 + 2k**	Me	2-Cl-5-NO_2_-Ph		**85**

^a^Reaction conditions: a mixture of tetralone (**5**) (5 mmol), arylhydrazonals **2a–d,k** (5 mmol) and NH_4_OAc (10 mmol) in AcOH (10 ml) was charged in the Q-tube reactor's 35 ml glass tube and heated for 30 min at 165 °C (oil bath).

**Table 5:** Some of the selected bond angles and bond lengths for **6b**.

Bond	Bond length(Å)	Bond	Bond angle(°)
C8–C9	1.376(8)	C6–C7–C8	122.0(6)
C5–C6	1.497(8)	C4–C5–C6	119.5(6)
C3–C4	1.501(8)	C3–C4–C5	115.4(6)
C2–C3	1.367(7)	C12–N1–C13	119.4(4)
N1–C12	1.334(6)	N2–C1–C13	119.2(4)
N2–C1	1.422(6)	N3–N2–C1	114.1(4)
N2–N3	1.254(6	N2–N3–C20	114.0(4)
N3–C20	1.417(6)	N3–C20–C25	117.9(5)
N4–C22	1.461(8)	O1–N4–O2	123.6(6)

**Scheme 2:**
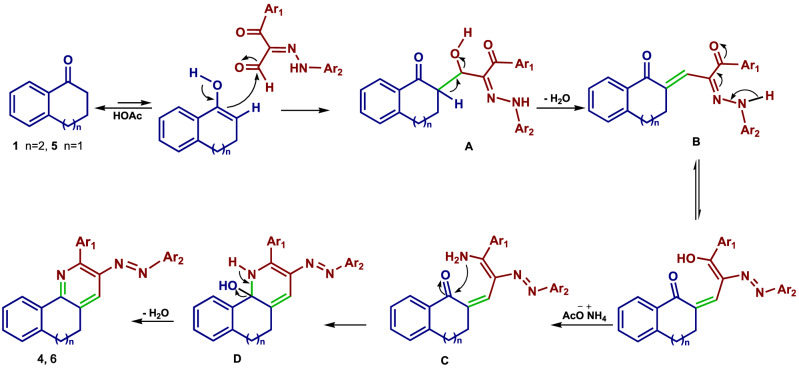
Mechanistic pathway postulated for the formation of compounds **4** and **6**.

As illustrated in Scheme 2, the mechanistic path of this ammonium acetate-mediated cyclocondensation comprises two consecutive condensation reactions. In this way, the enol form of benzosuberone (**1**) or tetralone (**5**) formed by AcOH induced enolization was subjected to nucleophilic addition to the arylhydrazonal aldehyde carbonyl-carbon to form the adduct **A** that generates the alkylidene intermediate **B** by losing one water molecule. Next, in the presence of ammonium acetate, this intermediate was converted to the non-isolable intermediate **C**. By the second nucleophilic addition, benzosuberone (**1**) or tetralone (**5**) carbonyl carbon was targeted by the NH_2_ moiety to form the adduct **D**, which lost the second water molecule to produce compounds **4** and **6**.

**Scheme 3:**
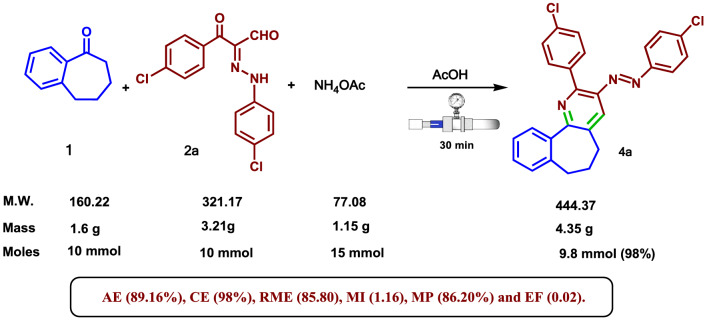
Scaled-up synthesis of 6,7-dihydro-5*H*-benzo[6,7]cyclohepta[1,2-*b*]pyridine **4a** and its green metrics.

Lastly, in order to demonstrate the practicality of the above-mentioned methodology, the current protocol was extended for a larger gram-scale preparation of 6,7-dihydro-5*H*-benzo[6,7]cyclohepta[1,2-*b*]pyridine **4a**, thus the reaction of benzosuberone (**1**) (10 mmol), arylhydrazonal **2a** (10 mmol), and NH_4_OAc (15 mmol) in AcOH (15 ml), was scaled up under the optimal reaction conditions, the required product **4a** was formed by crystallization from the dioxane/DMF mixture (2:1) in a 98% yield (Scheme 3). Furthermore, green metrics^47,48^ like CE (carbon efficiency), RME (reaction mass efficiency), AE (atom economy), MP (mass productivity), MI (mass intensity), and E-factor (EF) have been calculated for the assessment of the current strategy on the ‘greenness’ scale. The new methodology, as outlined in Scheme 3, has established a good combination of AE (89.16%), CE (98%), RME (85.80), MI (1.16), MP (86.20%) and EF (0.02). The reaction was scaled up and had good data on the 'greenness' scale, so that it could have some benefit in industrial chemistry.

### Anti-cancer screening (in vitro)

**Figure 6:**
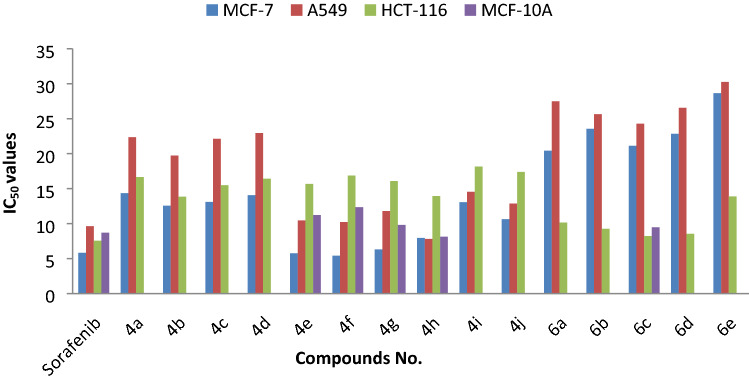
IC_50_ (μM) of **4a–j** and **6a–e** against the tested cancer cell lines.

In this study, the biological activity of the synthesized 6,7-dihydro-5*H*-benzo[6,7]cyclohepta[1,2-*b*]pyridine and 5,6-dihydrobenzo[*h*]quinoline derivatives was preliminary investigated as anti-cancer agents. In the initial stage of this endeavor, the cytotoxic activity of **4a–j** and **6a–e** was investigated for three cell lines of human cancer; lung cancer (A549), breast cancer (MCF-7) and colon cancer (HCT-116), beside normal breast cell line (MCF10A). This evaluation was conducted by utilizing the MTT colorimetric assay [3-(4,5-dimethyl-thiazol-2-yl)-2,5-diphenyltetrazoliumbromide]^49,50^, and the use of sorafenib as an anti-cancer reference drug, during this screening, three independent determinations were applied for three concentrations (12.5, 25 and 50 μM) of the synthesized compounds for an incubation time of 48 h, since the concentration of less than 12.5 μM did not have a significant effect. The obtained results have been analyzed and interpreted in order to calculate IC_50_ values for the newly synthesized compounds, and the results were tabulated in Table 6. Many of the tested compounds in this investigation revealed promising cytotoxicity against the tested cancer cell lines. The IC_50_ values shown in Table 6 ranged from 5.42–30.25 μM, whereas the sorafenib (reference drug) displays IC_50_ ranged from 5.84–9.63 μM against the tested cancer cell lines (Fig. 6). Initially, compounds **4e** and **4f** for the MCF-7 cell line displayed the highest potent cytotoxic activity with respective IC_50_ values 5.76 and 5.42 μM, while compounds **4g** and **4h** exhibited IC_50_ (6.31, 7.95 μM) respectively. The remaining compounds showed moderate to less cytotoxic activity. These results have shown that the compounds containing the benzosuberone moiety, {6,7-dihydro-5*H*-benzo[6,7]cyclohepta[1,2-*b*]pyridine **4a–j}** exhibit more promising cytotoxic activity against the tested cell lines than those containing tetralone moiety {5,6-dihydrobenzo[*h*]quinoline derivatives **6a–e**}. Furthermore, the nature of the substituent on both aryl moieties of compounds **4a–j** played an important role in this cytotoxic activity, as noted by the presence of the electron-withdrawing substituent on the aryl moiety at 2-position (Ar_1_) enhances this activity (NO_2_ > Cl > Br), compared with the phenyl or aryl moiety substituted with the electron-donating group (OMe) and even better than the thiophen moiety at 2-position, also the combination of two electron-withdrawal substituents on Ar_2_ improves the cytotoxic behavior better than the mono-substituted *N*-aryl moiety in the following order (F, NO_2_ > di-F > Cl, NO_2_ > Cl). On the other hand, compound **4h** which incorporates Ar_2_ = 2,4-difluorophenyl, showed better cytotoxic activity (IC_50_ = 7.82 μM) against the A549 cell line reflecting the importance of the difluoro substituent on the activity against this type of cancer cell line, while compounds **4e,f** showed IC_50_ values (10.45, 10.23 μM). Moreover, compounds **4a–j** displayed lower cytotoxicity towards the HCT-116 cell line, but few examples of the benzo[*h*]quinoline series such as compounds **6b–d**, showed good cytotoxic properties with IC_50_ 9.26, 8.22 and 8.55 μM respectively.

**Table 6:** IC_50_ (μM) for **4a–j** and **6a–e** against the tested cancer cell lines.

Compound	MCF-7	A549	HCT-116
4a	14.36 ± 0.87	22.36 ± 1.39	16.65 ± 1.09
4b	12.57 ± 1.41	19.73 ± 2.18	13.85 ± 1.44
4c	13.11 ± 2.05	22.13 ± 1.58	15.49 ± 1.72
4d	14.06 ± 1.13	22.95 ± 1.36	16.43 ± 2.11
4e	5.76 ± 1.53	10.45 ± 1.04	15.67 ± 1.68
4f	5.42 ± 1.40	10.23 ± 1.77	16.88 ± 0.85
4g	6.31 ± 1.08	11.79 ± 1.82	16.09 ± 1.67
4h	7.95 ± 1.95	7.82 ± 1.66	13.94 ± 1.18
4i	13.07 ± 1.64	14.55 ± 1.62	18.16 ± 1.30
4j	10.63 ± 1.77	12.87 ± 2.03	17.39 ± 1.84
6a	20.42 ± 1.39	27.48 ± 0.98	10.15 ± 0.97
6b	23.55 ± 1.45	25.65 ± 1.19	9.26 ± 1.51
6c	21.13 ± 1.73	24.29 ± 1.50	8.22 ± 1.25
6d	22.84 ± 0.97	26.56 ± 1.82	8.55 ± 1.17
6e	28.65 ± 2.16	30.25 ± 1.33	13.89 ± 1.48
Sorafenib	5.84 ± 0.87	9.63 ± 1.12	7.56 ± 1.08

The compounds that had the best cytotoxicity against the cancer cell lines used in this research, such as **4e**, **4f**, **4g**, **4h** and **6c**, were tested against the normal breast cell line (MCF-10A) by applying the same procedure. The obtained IC_50_ values for this normal cell (MCF-10A) presented encouraging results as their values [**4e** (11.23 ± 1.42 μM), **4f** (12.36 ± 1.69 μM), **4g** (9.81 ± 0.097 μM), **4h** (8.13 ± 1.84 μM), **6c** (9.47 ± 2.16 μM)] while sorafenib IC_50_ (8.71 ± 1.43) when applied to the MCF-10A cell line.

## Conclusion

In summary, the above-mentioned research study developed an efficient high-pressurized Q-tube assisted protocol for synthesizing unprecedented series of 6,7-dihydro-5*H*-benzo[6,7]cyclohepta[1,2-*b*]pyridine and 5,6-dihydrobenzo[*h*]quinolines via ammonium acetate-mediated cyclocondensation reactions of 3-oxo-2-arylhydrazonopropanals with benzosuberone, and tetralone precursors respectively, using the high-pressure Q-tube reactor as a secure, efficient and environmentally benign tool. Besides these features, the process has good data based on the scale of ‘greenness’ such as atomic economy, carbon efficiency and E-factor. It has been shown that members from the synthesized 6,7-dihydro-5*H*-benzo[6,7]cyclohepta[1,2-*b*]pyridine display promising cytotoxicities against breast (MCF-7), and lung (A549) cancerous cells, while HCT-116 (colon) cancer cells have been inhibited by certain examples of 5,6-dihydrobenzo[*h*]quinoline derivatives. Accordingly, these primary promising findings could serve as a key basis for further investigations to design a patent anti-cancer drug.

## Experimental

### General

Melting points were recorded on a Griffin melting point apparatus and are uncorrected. IR spectra were recorded using KBr disks and a Jasco FT-IR-6300 spectrophotometer. ^1^H NMR (400 MHz) or (600 MHz) and ^13^C{^1^H} NMR (100 MHz) or (150 MHz) spectra were recorded at 25 °C using CDCl_3_ or DMSO-*d*_*6*_ as solvents with TMS as an internal standard on a Bruker DPX 400 or 600 super-conducting NMR spectrometer. Chemical shifts (*δ*) are reported in ppm. Low-resolution electron impact mass spectra [MS (EI)] and high-resolution electron impact mass spectra [HRMS (EI)] were performed using a high-resolution GC–MS (DFS) thermo spectrometer at 70.1 eV and a magnetic sector mass analyzer. Following the courses of reactions and checking homogeneity of products were performed using thin layer chromatography (TLC). The Q-tube assisted reactions were performed in a Q-tube pressure monitor safe reactor from Q Labtech (distributed by Sigma-Aldrich), equipped with stainless steel adapter attached with pressure gauge (300 psi), high pressure adapter (180 psi), a needle adapter, a borosilicate glass pressure tube (35 mL), Teflon sleeve, a PTFE faced silicon septa and a catch bottle. The microwave irradiation was performed on a single mode cavity Explorer Microwave synthesizer (CEM Corporation, NC, USA). The X-ray crystallographic data were collected by using a Bruker X8 Prospector at room temperature by using Cu-Kα radiation. The structures were solved by using direct methods and expanded using Fourier techniques. The non-hydrogen atoms were refined anisotropically. The structures were solved and refined using the Bruker SHELXTL Software Package (Structure solution program—SHELXS-97 and Refinement program—SHELXL-97)^51^. Data were corrected for the absorption effects using the multi-scan method (SADABS). The crystal image was created by the software Mercury (version 3.8)^52^. The three human cancer cell lines including HCT-116 (colon cancer), MCF-7 (breast cancer) and A549 (lung cancer) were obtained from the American Type Culture Collection (ATCC).

### General method for preparing compounds 4a–j and 6a–e

A mixture of benzosuberone (**1**), tetralone (**5**) (5 mmol), arylhydrazonals **2** (5 mmol), NH_4_OAc (10 mmol) and glacial AcOH (10 ml) was charged in the glass tube (35 ml) of the Q-tube reactor (Labtech) then a septa was mounted on the top of each tube and the required cap and pressure adapter were utilized. The mixtures were heated for 30 min at 165 °C (oil bath). The Progression of each reaction was tracked utilizing GC/MS and TLC. After cooling to room temperature, the solids formed were filtered off, washed with EtOH and re-crystallized from the proper solvent (as shown below) to provide 6,7-dihydro-5*H*-benzo[6,7]cyclohepta[1,2-*b*]pyridine **4a–j** and 5,6-dihydrobenzo[*h*]quinoline **6a–e** as pure products .

- **Method B for preparing compound 4a.**
  A mixture of benzosuberone (**1**) (2 mmol), arylhydrazonal **2** (2 mmol), NH_4_OAc (4 mmol) and glacial AcOH (5 ml) was charged in the microwave glass tube (10 ml) and irradiated by MW (250 W) at 130 °C for 30 min. The formed solid upon cooling was filtered off, washed with EtOH and re-crystallized from dioxane/DMF mixture (2:1), to provide **4a** as reddish-brown crystal, yield: 0.61 g (69%).
- **Method C for preparing compound 4a.**
  A mixture of benzosuberone (**1**) (5 mmol), arylhydrazonal **2** (5 mmol), NH_4_OAc (10 mmol) and glacial AcOH (10 ml) was charged in round bottomed flask (25 ml) fitted with condenser and refluxed at normal pressure 4 h. The formed solid upon cooling was filtered off, washed with EtOH and re-crystallized from dioxane/DMF mixture (2:1), to provide **4a** as deep reddish orange crystal, yield: 0.90 g (41%).

#### (E)-(4-Chlorophenyl)-[2-(4-chlorophenyl)-6,7-dihydro-5H-benzo[6,7]cyclohepta[1,2-b]pyridin-3-yl]diazene (4a)

Recrystallized from dioxane/DMF mixture (2:1), reddish orange crystal, yield: 2.15 g (98%, Q-tube), m.p. 215–216 °C; IR (KBr): *v*/cm^−1^ 1652 (C=N), 1577 (N=N); ^1^H-NMR (600 MHz, CDCl_3_): *δ* = 2.34 (p, 2H, H of CH_2_), 2.65–2.69 (m, 4H, H of CH_2_), 7.31(d, *J* = 7.8 Hz, 1H, Ar–H), 7.42–7.46 (m, 2H, Ar–H), 7.48 (d, *J* = 8.4 Hz, 2H, Ar–H), 7.53 (d, *J* = 8.4 Hz, 2H, Ar–H), 7.84–7.87 (m, 4H, Ar–H), 7.93 (d, *J* = 7.8 Hz, 1H, Ar–H), 7.99 (s, 1H, pyridine H-4); ^13^C{^1^H} NMR (150 MHz, CDCl_3_): *δ* = 30.46, 31.33, 32.80 (3*C*H_2_), 124.74, 124.79, 127.15, 128.18, 128.92, 129.65, 129.83, 129.96, 130.69, 132.94, 135.38, 135.70, 137.89, 138.98, 139.96, 144.38, 151.42, 154.01, 160.96; MS (EI): *m/z* (%) 445 (M^+^ + 2, 66.59), 444 (M^+^ + 1, 89.95), 443 (M^+^, 99.85), 442 (M^+^ − 1, 100.00). HRMS (EI): *m/z* calcd. for C_26_H_19_Cl_2_N_3_ (M^+^) 443.0951, found 443.0950. Crystal Data, moiety formula: C_26_H_19_Cl_2_N_3_, M = 444.36, triclinic, a = 8.726(1) Å, b = 11.072(2) Å, c = 12.059(2) Å, V = 1063.6(3) Å^3^, α = 104.412(8)°, β = 104.064(8)°, γ = 99.295(7)°, space group: P-1 (#2), Z = 2, D_calc_ = 1.387 g·cm^−3^, No. of reflection measured: 9587, unique: 4334, 2θ _max_ = 52.7°, R1 = 0.0493 (CCDC 2013410)^53^.

#### (E)-(2-Fluoro-5-nitrophenyl)-(2-phenyl-6,7-dihydro-5H-benzo[6,7]cyclohepta[1,2-b]pyridin-3-yl)diazene (4b)

Recrystallized from dioxane/DMF mixture (3:1), deep reddish orange crystal, yield: 1.23 g (94%), m.p. 253–254 °C; IR (KBr): *v*/cm^−1^ 1635 (C=N), 1585 (N=N); ^1^H-NMR (600 MHz, CDCl_3_): *δ* = 2.35 (p, 2H, H of CH_2_), 2.66–2.71 (m, 4H, H of CH_2_), 7.31 (d, *J* = 7.8 Hz, 1H, Ar–H), 7.43–7.50 (m, 3H, Ar–H), 7.53–7.57 (m, 3H, Ar–H), 7.91 (d, *J* = 8.4 Hz, 2H, Ar–H), 7.98 (d, *J* = 7.8 Hz, 1H, Ar–H), 8.07 (s, 1H, pyridine H-4), 8.36–8.39 (m, 1H, Ar–H), 8.50–8.51 (m, 1H, Ar–H); ^13^C{^1^H} NMR (150 MHz, CDCl_3_): *δ* = 30.45, 31.36, 32.75 (3*C*H_2_), 114.73, (118.33, 118.48) (d, ^2^*J*_CF_ = 22.5 Hz), 124.36, (127.11, 127.18) (d, ^3^*J*_CF_ = 10.5 Hz), 128.11, 128.46, 128.92, 129.48, 129.75, 130.06, 130.42, 131.72, 135.42, 140.02, (141.01, 141.07) (d, ^3^*J*_CF_ = 9.0 Hz), 144.32, 144.97, 156.66, 162.54, (162.45, 164.23) (d, ^1^*J*_CF_ = 267 Hz); MS (EI): *m/z* (%) 440 (M^+^ + 2, 5.01), 439 (M^+^ + 1, 25.98), 438 (M^+^, 100.00), 437 (M^+^ − 1, 64.39). HRMS (EI): *m/z* calcd. for C_26_H_19_FN_4_O_2_ (M^+^) 438.1487, found 438.1487. Crystal Data, moiety formula: C_26_H_19_FN_4_O_2_, M = 438.45, monoclinic, a = 14.9070(3) Å, b = 9.3911(2) Å, c = 15.6885(3) Å, V = 2151.27(8) Å^3^, α = γ = 90°, β = 101.6200(10)°, space group: P 1 21/c 1, Z = 4, D_calc_ = 1.354 g·cm^−3^, No. of reflection measured: 34,297, unique: 3755, θ _max_ = 66.60°, R1 = 0.0526 (CCDC 2013411)^53^.

#### (*E*)-(2-Chloro-5-nitrophenyl)-[2-(4-chlorophenyl)-6,7-dihydro-5*H*-benzo[6,7]cyclohepta[1,2-*b*]pyridin-3-yl]diazene (4c)

Recrystallized from dioxane/DMF mixture (1:3), reddish brown crystal, yield: 1.45 g (99%), m.p. 250–251 °C; IR (KBr): *v*/cm^−1^ 1637 (C=N), 1579 (N=N); ^1^H-NMR (600 MHz, CDCl_3_): *δ* = 2.28 (p, 2H, H of CH_2_), 2.58–2.63 (m, 4H, H of CH_2_), 7.24 (d, *J* = 7.2 Hz, 1H, Ar–H), 7.36–7.40 (m, 2H, Ar–H), 7.44 (d, *J* = 8.0 Hz, 2H, Ar–H), 7.71 (d, *J* = 8.4 Hz, 1H, Ar–H), 7.80 (d, *J* = 8.0 Hz, 2H, Ar–H), 7.86 (d, *J* = 7.8 Hz, 1H, Ar–H), 7.98 (s, 1H, pyridine H-4), 8.20 (dd, *J* = 2.8, 8.8 Hz, 1H, Ar–H), 8.32 (d, *J* = 2.4 Hz, 1H, Ar–H); ^13^C{^1^H} NMR (150 MHz, CDCl_3_): *δ* = 30.46, 31.33, 32.69 (3*C*H_2_), 113.45, 124.45, 125.57, 127.18, 128.35, 128.94, 129.53, 130.03, 131.93, 132.86, 135.63, 135.74, 136.33, 139.38, 139.97, 141.82, 144.23, 147.43, 149.26, 155.51, 162.86; MS (EI): *m/z* (%) 490 (M^+^ + 2, 68.09), 489 (M^+^ + 1, 75.13), 488 (M^+^, 100.00), 487 (M^+^ − 1, 71.68). HRMS (EI): *m/z* calcd. for C_26_H_18_Cl_2_N_4_O_2_ (M^+^) 488.0801, found 488.0800.

#### (*E*)-[2-(4-Bromophenyl)-6,7-dihydro-5*H*-benzo[6,7]cyclohepta[1,2-*b*]pyridin-3-yl]-(2-chloro-5-nitrophenyl)diazene (4d)

Recrystallized from DMF mixture as reddish brown crystal, yield: 1.53 g (96%), m.p. 256–257 °C; IR (KBr): *v*/cm^−1^ 1639 (C=N), 1568 (N=N); ^1^H-NMR (600 MHz, CDCl_3_): *δ* = 2.28 (p, 2H, H of CH_2_), 2.58–2.64 (m, 4H, H of CH_2_), 7.24 (d, *J* = 7.2 Hz, 1H, Ar–H), 7.35–7.42 (m, 2H, Ar–H), 7.60 (d, *J* = 8.4 Hz, 2H, Ar–H), 7.71–7.74 (m, 3H, Ar–H), 7.86 (d, *J* = 7.2 Hz, 1H, Ar–H), 7.98 (s, 1H, pyridine H-4), 8.21 (dd, *J* = 2.8, 8.8 Hz, 1H, Ar–H), 8.33 (d, *J* = 2.4 Hz, 1H, Ar–H); ^13^C{^1^H} NMR (150 MHz, CDCl_3_): *δ* = 30.28, 31.14, 32.50 (3*C*H_2_), 113.27, 124.22, 125.38, 127.00, 128.75, 129.32, 129.81, 130.72, 131.12, 131.74, 132.93, 135.44, 136.71, 139.28, 139.78, 141.62, 144.02, 147.25, 149.08, 155.39, 162.74; MS (EI): *m/z* (%) 534 (M^+^ + 2, 100.00), 533 (M^+^ + 1, 84.97), 532 (M^+^, 73.59), 531 (M^+^ − 1, 48.91). HRMS (EI): *m/z* calcd. for C_26_H_18_BrClN_4_O_2_ (M^+^) 532.0296, found 532.0296. Crystal Data, moiety formula: C_26_H_18_BrClN_4_O_2_, M = 533.81, triclinic, a = 9.058(2) Å, b = 10.791(3) Å, c = 13.031(3) Å, V = 1154.9(5) Å^3^, α = 103.776(8)°, β = 94.977(7)°, γ = 108.471(8)°, space group: P-1, Z = 2, D_calc_ = 1.535 g·cm^−3^, No. of reflection measured: 9158, unique: 4061, 2θ _max_ = 50.0°, R1 = 0.0501 (CCDC 2013412)^53^.

#### (*E*)-[2-(4-Chlorophenyl)-6,7-dihydro-5*H*-benzo[6,7]cyclohepta[1,2-*b*]pyridin-3-yl]-(2-fluoro-5-nitrophenyl)diazene (4e)

Recrystallized from dioxane/DMF mixture (1:3), reddish orange crystal, yield: 1.35 g (97%), m.p. 202–203 °C; IR (KBr): *v*/cm^−1^ 1619 (C=N), 1581 (N=N); ^1^H-NMR (600 MHz, CDCl_3_): *δ* = 2.35 (p, 2H, H of CH_2_), 2.66–2.71 (m, 4H, H of CH_2_), 7.32 (d, *J* = 7.2 Hz, 1H, Ar–H), 7.47–7.52 (m, 3H, Ar–H), 7.54 (d, *J* = 7.8 Hz, 2H, Ar–H), 7.88 (d, *J* = 7.8 Hz, 2H, Ar–H), 7.95 (dd, *J* = 1.2, 8.4 Hz, 1H, Ar–H), 8.07 (s, 1H, pyridine H-4), 8.39–8.42 (m, 1H, Ar–H), 8.50–8.51 (m, 1H, Ar–H); ^13^C{^1^H} NMR (150 MHz, CDCl_3_): *δ* = 30.44, 31.33, 32.72 (3*C*H_2_), 114.69, (118.43, 118.57) (d, ^2^*J*_CF_ = 21 Hz), 124.48, 127.21 (127.29, 127.35) (d, ^3^*J*_CF_ = 9.0 Hz), 128.38, 128.98, 129.09, 129.65, 130.17, 130.67, 132.92, 135.75, (135.87, 135.94) (d, ^3^*J*_CF_ = 10.5 Hz), 139.05, 140.02, 144.29, 155.12, 155.32, 160.31, (162.68, 164.28) (d, ^1^*J*_CF_ = 240 Hz); MS (EI): *m/z* (%) 474 (M^+^ + 2, 36.21), 473 (M^+^ + 1, 52.09), 472 (M^+^, 100.00), 471 (M^+^ − 1, 75.96). HRMS (EI): *m/z* calcd. for C_26_H_18_ClFN_4_O_2_ (M^+^) 472.1097, found 472.1095.

#### (*E*)-(2-Fluoro-5-nitrophenyl)-[2-(4-nitrophenyl)-6,7-dihydro-5*H*-benzo[6,7]cyclohepta[1,2-*b*]pyridin-3-yl]diazene (4f)

Recrystallized from dioxane/DMF mixture (1:3), red crystal, yield: 1.40 g (95%), m.p. 214–215 °C; IR (KBr): *v*/cm^−1^ 1622 (C=N), 1581 (N=N); ^1^H-NMR (400 MHz, DMSO-*d*_*6*_): *δ* = 2.27 (p, *J* = 6.8 Hz, 2H, H of C*H*_2_), 2.57 (t, *J* = 6.8 Hz, 2H, H of C*H*_2_), 2.63 (t, *J* = 6.8 Hz, 2H, H of C*H*_2_), 7.38–7.40 (m, 1H, Ar–H), 7.46–7.49 (m, 2H, Ar–H), 7.83–7.88 (m, 2H, Ar–H), 8.10 (s, 1H, pyridine H-4), 8.14 (d, *J* = 8.8 Hz, 2H, Ar–H), 8.25–8.27 (m, 1H, Ar–H), 8.35 (d, *J* = 8.8 Hz, 2H, Ar–H), 8.47–8.50 (m, 1H, Ar–H); ^13^C{^1^H} NMR (100 MHz, DMSO-*d*_*6*_): *δ* = 29.41, 30.44, 32.04 (3*C*H_2_), 113.79, (119.01, 119.23) (d, ^2^*J*_CF_ = 22 Hz), 122.68, 124.04, 126.76 (128.21, 128.31) (d, ^3^*J*_CF_ = 10.0 Hz), 128.84, 129.09, 129.88, 132.47, 136.45, 138.59, 139.58, (139.72, 139.80) (d, ^3^*J*_CF_ = 8.0 Hz), 143.70, 143.79, 144.39, 147.50, 153.26, 161.92, (161.11, 163.75) (d, ^1^*J*_CF_ = 264 Hz); MS (EI): *m/z* (%) 484 (M^+^ + 1, 21.84), 483 (M^+^, 100.00), 482 (M^+^ − 1, 64.07). HRMS (EI): *m/z* calcd. for C_26_H_18_FN_5_O_4_ (M^+^) 483.1337, found 483.1337.

#### (*E*)-[2-(4-Bromophenyl)-6,7-dihydro-5*H*-benzo[6,7]cyclohepta[1,2-*b*]pyridin-3-yl]-(2-fluoro-5-nitrophenyl)diazene (4g)

Recrystallized from dioxane/DMF mixture (1:3), reddish brown crystal, yield: 1.50 g (96%), m.p. 234–235 °C; IR (KBr): *v*/cm^−1^ 1624 (C=N), 1574 (N=N); ^1^H-NMR (600 MHz, CDCl_3_): *δ* = 2.35 (p, 2H, H of CH_2_), 2.66–2.71 (m, 4H, H of CH_2_), 7.32 (d, *J* = 7.8 Hz, 1H, Ar–H), 7.44–7.52 (m, 3H, Ar–H), 7.68 (d, *J* = 8.4 Hz, 2H, Ar–H), 7.81 (d, *J* = 8.4 Hz, 2H, Ar–H), 7.94 (dd, *J* = 1.2, 8.4 Hz, 1H, Ar–H), 8.05 (s, 1H, pyridine H-4), 8.39–8.42 (m, 1H, Ar–H), 8.50–8.51 (m, 1H, Ar–H); ^13^C{^1^H} NMR (150 MHz, CDCl_3_): *δ* = 30.49, 31.25, 32.73 (3*C*H_2_), 114.71, (118.54, 118.69) (d, ^2^*J*_CF_ = 22.5 Hz), 125.06, 125.92, 127.15, 127.24, (127.69, 127.76) (d, ^3^*J*_CF_ = 10.5 Hz), 129.09, 130.32, (130.92, 130.97) (d, ^3^*J*_CF_ = 7.5 Hz), 131.41, 132.07, 133.42, 136.58, 137.01, 139.69, 140.15, 144.37, 154.74, 158.99, 161.80, (162.45, 164.22) (d, ^1^*J*_CF_ = 265.5 Hz); MS (EI): *m/z* (%) 518 (M^+^ + 2, 100.00), 517 (M^+^ + 1, 89.13), 516 (M^+^, 98.02), 515 (M^+^ − 1, 60.04). HRMS (EI): *m/z* calcd. for C_26_H_18_BrFN_4_O_2_ (M^+^) 516.0592, found 516.0591.

#### (*E*)-[2-(4-Bromophenyl)-6,7-dihydro-5*H*-benzo[6,7]cyclohepta[1,2-*b*]pyridin-3-yl]-(2,4-difluorophenyl)diazene (4h)

Recrystallized from dioxane/DMF mixture (1:2), red crystal, yield: 1.45 g (99%), m.p. 185–186 °C; IR (KBr): *v*/cm^−1^ 1631 (C=N), 1579 (N=N); ^1^H-NMR (400 MHz, CDCl_3_): *δ* = 2.50 (p, 2H, H of CH_2_), 2.80–2.86 (m, 4H, H of CH_2_), 7.15 (t, *J* = 8.0 Hz, 1H, Ar–H), 7.24 (t, *J* = 8.0 Hz, 1H, Ar–H), 7.45–7.48 (m, 1H, Ar–H), 7.58–7.65 (m, 2H, Ar–H), 7.79–7.86 (m, 3H, Ar–H), 7.95 (d, *J* = 8.4 Hz, 2H, Ar–H), 8.09 (d, *J* = 7.6 Hz, 1H, Ar–H), 8.17 (s, 1H, pyridine H-4); ^13^C{^1^H} NMR (150 MHz, CDCl_3_): *δ* = 30.13, 31.07, 32.47 (3*C*H_2_), (105.02, 105.26, 105.51) ( t, ^2^*J*_CF_ = 25.0 Hz), [(112.08, 112.11), (112.30, 112.33)] (dd, ^2^*J*_CF_ = 3.0, 25.0 Hz), (119.23, 119.33) (d, ^3^*J*_CF_ = 10.0 Hz), 123.46, 124.47, 126.87, 128.66, 129.27, 129.65, 130.85, 131.47, 132.91, 135.45, (137.69, 137.80) (d, ^3^*J*_CF_ = 11.0 Hz), 139.70, 144.24, 154.03, 159.50, 161.05, (162.10, 162.22) (d, ^3^*J*_CF_ = 11.0 Hz), [(163.54, 163.66), (166.09, 166.21)] (dd, ^1^*J*_CF_ = 12.0, 255.0 Hz); MS (EI): *m/z* (%) 491 (M^+^ + 2, 91.03), 490 (M^+^ + 1, 100.00), 489 (M^+^, 93.65), 488 (M^+^ − 1, 94.89). HRMS (EI): *m/z* calcd. for C_26_H_18_BrF_2_N_3_ (M^+^) 489.0647, found 489.0645.

#### (E)-(2-Fluoro-5-nitrophenyl)-[2-(4-methoxyphenyl)-6,7-dihydro-5H-benzo[6,7]cyclohepta[1,2-b]pyridin-3-yl]diazene (4i)

Recrystallized from dioxane/DMF mixture (2:1), reddish brown crystal, yield: 1.30 g (91%), m.p. 215–216 °C; IR (KBr): *v*/cm^−1^ 1639 (C=N), 1580 (N=N); ^1^H-NMR (400 MHz, DMSO-*d*_*6*_): *δ* = 2.24 (p, 2H, H of CH_2_), 2.56–2.58 (m, 4H, H of CH_2_), 3.87 (s, 3H, OC*H*_3_), 7.07–7.14 (m, 3H, Ar–H), 7.38 (d, *J* = 7.6 Hz, 1H, Ar–H), 7.46 (t, *J* = 7.6 Hz, 1H, Ar–H), 7.78–7.87 (m, 3H, Ar–H), 7.99 (d, *J* = 8.0 Hz, 1H, Ar–H), 8.15 (s, 1H, pyridine H-4), 8.29–8.31 (m, 1H, Ar–H), 8.44–8.45 (m, 1H, Ar–H); ^13^C{^1^H} NMR (150 MHz, CDCl_3_): *δ* = 30.47, 31.31, 32.72 (3*C*H_2_), 57.93 (O*C*H_*3*_), 114.74, (118.45, 118.60) (d, ^2^*J*_CF_ = 22.5 Hz), 124.28, 126.02, (127.21, 127.28) (d, ^3^*J*_CF_ = 10.5 Hz), 128.18, 128.59, 128.97, 129.61, 129.83, 130.17, 130.55, 131.89, 135.61, 140.21, (140.98, 141.05) (d, ^3^*J*_CF_ = 10.5 Hz), 144.46, 145.13, 156.85, 162.61, (162.70, 164.46) (d, ^1^*J*_CF_ = 264.0 Hz); MS (EI): *m/z* (%) 469 (M^+^ + 1, 28.35), 468 (M^+^, 100), 467 (M^+^ − 1, 72.69). HRMS (EI): *m/z* calcd. for C_27_H_21_FN_4_O_3_ (M^+^) 468.1592, found 468.1592.

#### (*E*)-(2-Fluoro-5-nitrophenyl)-(2-thiophen-2-yl-6,7-dihydro-5*H*-benzo[6,7]cyclohepta[1,2-*b*]pyridin-3-yl)diazene (4j)

Recrystallized from dioxane/DMF mixture (1:2), reddish brown crystal, yield: 1.22 g (93%), m.p. 238–239 °C; IR (KBr): *v*/cm^−1^ 1639 (C=N), 1573 (N=N); ^1^H-NMR (400 MHz, DMSO-*d*_*6*_): *δ* = 2.23 (p, 2H, H of CH_2_), 2.55–2.57 (m, 4H, H of CH_2_), 7.25 (t, *J* = 7.2 Hz, 1H, Ar–H), 7.38–7.50 (m, 3H, Ar–H), 7.80–7.88 (m, 3H, Ar–H), 7.97–7.98 (m, 2H, 1 Ar–H and pyridine H-4), 8.51–8.55 (m, 1H, Ar–H), 8.59–8.61 (m, 1H, Ar–H); ^13^C{^1^H} NMR (100 MHz, DMSO-*d*_*6*_): *δ* = 30.36, 31.28, 32.67 (3*C*H_2_), 114.79, (118.25, 118.49) (d, ^2^*J*_CF_ = 24.0 Hz), 124.53, (126.95, 127.07) (d, ^3^*J*_CF_ = 12.0 Hz), 128.29, 128.39, 128.98, 129.36, 129.88, 130.24, 130.65, 131.93, 135.18, 140.61, (141.23, 141.35) (d, ^3^*J*_CF_ = 12.0 Hz), 144.51, 145.13, 156.89, 162.48, (161.59, 164.24) (d, ^1^*J*_CF_ = 265.0 Hz); MS (EI): *m/z* (%) 445 (M^+^ + 1, 30.12), 444 (M^+^, 100.00), 443 (M^+^ − 1, 74.06). HRMS (EI): *m/z* calcd. for C_24_H_17_FN_4_O_2_S (M^+^) 444.1051, found 444.1050.

#### (*E*)-1-(4-Chlorophenyl)-2-[2-(4-chlorophenyl)-5,6-dihydrobenzo[*h*]quinolin-3-yl]diazene (6a)

Recrystallized from dioxane, reddish pink crystal, yield: 1.20 g (94%), m.p. 205–206 °C; IR (KBr): *v*/cm^−1^ 1639 (C=N), 1581 (N=N); ^1^H-NMR (600 MHz, CDCl_3_): *δ* = 2.96–3.02 (m, 4H, H of CH_2_), 7.22 (d, *J* = 8.4 Hz, 1H, Ar–H), 7.32–7.35 (m, 2H, Ar–H), 7.40–7.44 (m, 4H, Ar–H), 7.74 (d, *J* = 8.4 Hz, 2H, Ar–H), 7.79 (d, *J* = 8.4 Hz, 2H, Ar–H), 7.86 (s, 1H, pyridine H-4), 8.46 (d, *J* = 8.4 Hz, 1H, Ar–H); ^13^C{^1^H} NMR (150 MHz, CDCl_3_): *δ* = 27.99, 28.11 (2*C*H_2_), 123.20, 124.69, 126.07, 127.50, 128.07, 128.18, 129.74, 130.24, 131.97, 132.83, 134.10, 135.05, 137.04, 137.54, 138.95, 144.33, 151.38, 154.53, 154.75; MS (EI): *m/z* (%) 431 (M^+^ + 2, 61.24), 430 (M^+^ + 1, 84.91), 429 (M^+^, 88.79), 428 (M^+^ − 1, 100.00). HRMS (EI): *m/z* calcd. for C_25_H_17_Cl_2_N_3_ (M^+^) 429.0794, found 429.0794.

#### (*E*)-1-(2-Fluoro-5-nitrophenyl)-2-(2-phenyl-5,6-dihydrobenzo[*h*]quinolin-3-yl)diazene (6b)

Recrystallized from dioxane, reddish orange crystal, yield: 1.21 g (95%), m.p. 243–244 °C; IR (KBr): *v*/cm^−1^ 1635 (C=N), 1585 (N=N); ^1^H-NMR (600 MHz, CDCl_3_): *δ* = 3.06–3.14 (m, 4H, H of CH_2_), 7.31 (d, *J* = 8.4 Hz, 1H, Ar–H), 7.41–7.49 (m, 3H, Ar–H), 7.55–7.60 (m, 3H, Ar–H), 7.94 (d, *J* = 8.4 Hz, 2H, Ar–H), 8.06 (s, 1H, pyridine H-4), 8.35–8.38 (m, 1H, Ar–H), 8.50 (dd, *J* = 3.0, 9.6 Hz, 1H, Ar–H), 8.62 (dd, *J* = 1.8, 8.4 Hz, 1H, Ar–H); ^13^C{^1^H} NMR (150 MHz, CDCl_3_): *δ* = 27.54, 27.87 (2*C*H_2_), 114.47, (118.11, 118.25) (d, ^2^*J*_CF_ = 21.0 Hz), 123.33, 126.61, (126.81, 126.88) (d, ^3^*J*_CF_ = 10.5 Hz), 127.44, 127.88, 128.05, 128.69, 129.37, 130.66, 131.55, 131.91, (133.36, 133.44) (d, ^3^*J*_CF_ = 12.0 Hz), 139.14, 140.79, 144.19, 144.70, 155.66, 156.81, (162.26, 164.04) (d, ^1^*J*_CF_ = 267 Hz); MS (EI): *m/z* (%) 425 (M^+^ + 1, 26.14), 424 (M^+^, 100.00), 423 (M^+^ − 1, 74.95). HRMS (EI): *m/z* calcd. for C_25_H_17_FN_4_O_2_ (M^+^) 424.1330, found 424.1330. Crystal Data, moiety formula: C_25_H_17_FN_4_O_2_, M = 424.43, monoclinic, a = 9.353(2) Å, b = 14.012(2) Å, c = 15.585(2) Å, V = 2036.8(5) Å^3^, α = γ = 90°, β = 94.265(7)°, space group: P2_1_/c (#14), Z = 4, D_calc_ = 1.384 g·cm^−3^, No. of reflection measured: 11,363, unique: 3578, 2θ _max_ = 50.1°, R1 = 0.0676 (CCDC 2013409)^53^.

#### (*E*)-1-(2-Chloro-5-nitrophenyl)-2-(2-(4-chlorophenyl)-5,6-dihydrobenzo[*h*]quinolin-3-yl)diazene (6c)

Recrystallized from dioxane, red crystal, yield: 1.40 g (98%), m.p. 269–270 °C; IR (KBr): *v*/cm^−1^ 1636 (C=N), 1581 (N=N); ^1^H-NMR (400 MHz, CDCl_3_): *δ* = 3.04–3.11 (m, 4H, H of CH_2_), 7.29 (d, *J* = 8.4 Hz, 1H, Ar–H), 7.39–7.42 (m, 2H, Ar–H), 7.52 (d, *J* = 8.0 Hz, 2H, Ar–H), 7.75 (d, *J* = 8.4 Hz, 1H, Ar–H), 7.88 (d, *J* = 8.0 Hz, 2H, Ar–H), 8.01 (s, 1H, pyridine H-4), 8.25 (dd, *J* = 2.8, 8.4 Hz, 1H, Ar–H), 8.36 (d, *J* = 2.8 Hz, 1H, Ar–H), 8.53 (dd, *J* = 2.8, 8.4 Hz, 1H, Ar–H); ^13^C{^1^H} NMR (100 MHz, CDCl_3_): *δ* = 27.84, 27.88 (2*C*H_2_), 113.25, 123.29, 125.25, 126.26, 127.43, 128.08, 128.13, 129.00, 130.54, 131.71, 131.99, 132.70, 133.76, 135.53, 136.46, 139.09, 141.60, 144.18, 149.09, 155.80, 156.07; MS (EI): *m/z* (%) 476 (M^+^ + 2, 68.97), 475 (M^+^ + 1, 78.29), 474 (M^+^, 100.00), 473 (M^+^ − 1, 84.93). HRMS (EI): *m/z* calcd. for C_25_H_16_Cl_2_N_4_O_2_ (M^+^) 474.0645, found 474.0644.

#### (*E*)-1-(2-(4-Bromophenyl)-5,6-dihydrobenzo[*h*]quinolin-3-yl)-2-(2-chloro-5-nitrophenyl)diazene (6d)

Recrystallized from dioxane/DMF mixture (2:1), reddish brown crystal, yield: 1.50 g (97%), m.p. 282–283 °C; IR (KBr): *v*/cm^−1^ 1640 (C=N), 1583 (N=N); ^1^H-NMR (400 MHz, CDCl_3_): *δ* = 3.06–3.14 (m, 4H, H of CH_2_), 7.30 (d, *J* = 8.8 Hz, 1H, Ar–H), 7.41–7.44 (m, 2H, Ar–H), 7.69 (d, *J* = 8.8 Hz, 2H, Ar–H), 7.77 (d, *J* = 8.8 Hz, 1H, Ar–H), 7.82 (d, *J* = 8.8 Hz, 2H, Ar–H), 8.03 (s, 1H, pyridine H-4), 8.27 (dd, *J* = 2.4, 8.8 Hz, 1H, Ar–H), 8.38 (d, *J* = 2.4 Hz, 1H, Ar–H), 8.56 (dd, *J* = 2.4, 8.8 Hz, 1H, Ar–H); ^13^C{^1^H} NMR (100 MHz, CDCl_3_): *δ* = 28.06, 28.12 (2*C*H_2_), 113.47, 123.57, 124.19, 125.48, 126.51, 127.65, 128.29, 129.36, 130.05, 130.80, 131.29, 131.92, 132.26, 133.16, 136.38, 139.30, 141.81, 144.25, 149.21, 155.75, 156.41; MS (EI): *m/z* (%) 520 (M^+^ + 2, 100.00), 519 (M^+^ + 1, 97.98), 518 (M^+^, 77.56), 517 (M^+^ − 1, 61.23). HRMS (EI): *m/z* calcd. for C_25_H_16_BrClN_4_O_2_ (M^+^) 518.0140, found 518.0139.

#### (*E*)-1-(2-Chloro-5-nitrophenyl)-2-(2-methyl-5,6-dihydrobenzo[*h*]quinolin-3-yl)diazene (6e)

Recrystallized from dioxane, deep orange crystal, yield: 0.95 g (85%), m.p. 248–249 °C; IR (KBr): *v*/cm^−1^ 1637 (C=N), 1575 (N=N); ^1^H-NMR (600 MHz, CDCl_3_): *δ* = 3.00–3.04 (m, 4H, H of CH_2_), 3.09 (s, 3H, CH_3_), 7.28 (d, *J* = 7.2 Hz, 1H, Ar–H), 7.38–7.43 (m, 2H, Ar–H), 7.77 (d, *J* = 8.4 Hz, 1H, Ar–H), 7.91 (s, 1H, pyridine H-4), 8.27 (dd, *J* = 3.0, 8.4 Hz, 1H, Ar–H), 8.49 (d, *J* = 7.2 Hz, 1H, Ar–H), 8.55 (d, *J* = 2.4 Hz, 1H, Ar–H); ^13^C{^1^H} NMR (150 MHz, CDCl_3_): *δ* = 20.95 (*C*H_3_), 27.95, 28.21 (2*C*H_2_), 113.17, 122.92, 125.30, 125.99, 126.32, 127.61, 128.21, 130.54, 131.83, 139.30, 141.66, 145.20, 147.45, 149.37, 156.10, 158.48; MS (EI): *m/z* (%) 380 (M^+^ + 2, 9.15), 379 (M^+^ + 1, 6.38), 378 (M^+^, 22.08). HRMS (EI): *m/z* calcd. for C_20_H_15_ClN_4_O_2_ (M^+^) 378.0878, found 378.0877.

### MTT assay (in vitro)^42^

The cytotoxicity of 15 substances **4a–j** and **6a–e** was tested for three cancer cell lines, including MCF-7 (breast cancer), A549 (lung cancer), and HCT-116 (colon cancer), plus MCF10A (breast cell) as non-malicious cell, using a colorimetric MTT assay. Cell lines were matured in a Minimum Critical Media (MEM) bolstered at 37 °C with 10% fetal bovine serum (FBS), streptomycin, and penicillin in a humid carbon dioxide (5%) environment. In DMSO, the compounds studied were dissolved and the resulting solutions were diluted by adding the culture medium to the necessary concentrations (DMSO final concentration 0.1%). In a media bolstered by (10%) FBS, each cell line (10^4^ cells/well) was dispersed and plated onto sterilized plates (96 well), then remedied with the compounds tested in this study (3 concentrations), and sorafenib. The control experiment was also conducted by treating cells with 0.1% DMSO. In all cases, the duration of incubation was 48 h (37 °C, 5% CO_2_). Then the medium was withdrawn and the MTT solution was applied and the plates were incubated for 4 h once more. The MTT solution was withdrawn after incubation, and the purple formazan dye obtained was dissolved in DMSO. Using a microplate spectrophotometer, the absorbance was eventually measured at 490 nm (triplicate-tests were conducted). The IC_50_ values (Table 6) were determined using the GraphPad Prism (7.0) software.

## Supplementary Information

Supplementary Figures.

Supplementary Information 1.

Supplementary Information 2.

Supplementary Information 3.

Supplementary Information 4.
