# Supplementary material for: High pressure assisted synthetic approach for novel 6,7-dihydro-5H-benzo[6,7]cyclohepta[1,2-b]pyridine and 5,6-dihydrobenzo[h]quinoline derivatives and their assessment as anticancer agents
Source: Sci Rep. 2020 Dec 10;10:21691. doi: 10.1038/s41598-020-78590-x (PMC7728779; doi:10.1038/s41598-020-78590-x)
Supplement: Supplementary file 1 — Supplementary Figures. [file 41598_2020_78590_MOESM1_ESM.pdf]

Supporting Information

For

High pressure assisted synthetic approach for novel 6,7-dihydro-5*H*-benzo[6,7]cyclohepta[1,2-*b*]pyridine and 5,6-dihydrobenzo[*h*]quinoline derivatives and their assessment as anticancer agents

Haider Behbehani,*[1] Fatemah A. Aryan,*[2] Kamal M. Dawood,[3] and Hamada Mohamed Ibrahim,*[1,4]

[1]Chemistry Department, Faculty of Science, Kuwait University, P.O. Box 5969, Safat 13060, Kuwait.
[2] Authority of Applied Education and Training, Basic Education College, Science Department P.O. Box 23167, Safat 13092, Kuwait.
[3]Chemistry Department, Faculty of Science, Cairo University, Giza 12613, Egypt.
[4]Chemistry Department, Faculty of Science, Fayoum University, P.O. Box 63514, Fayoum, Egypt.

Contents

1- Copies of MS, ^{1}H MR, and ^{13}C NMR for the reported compounds. S2-S43

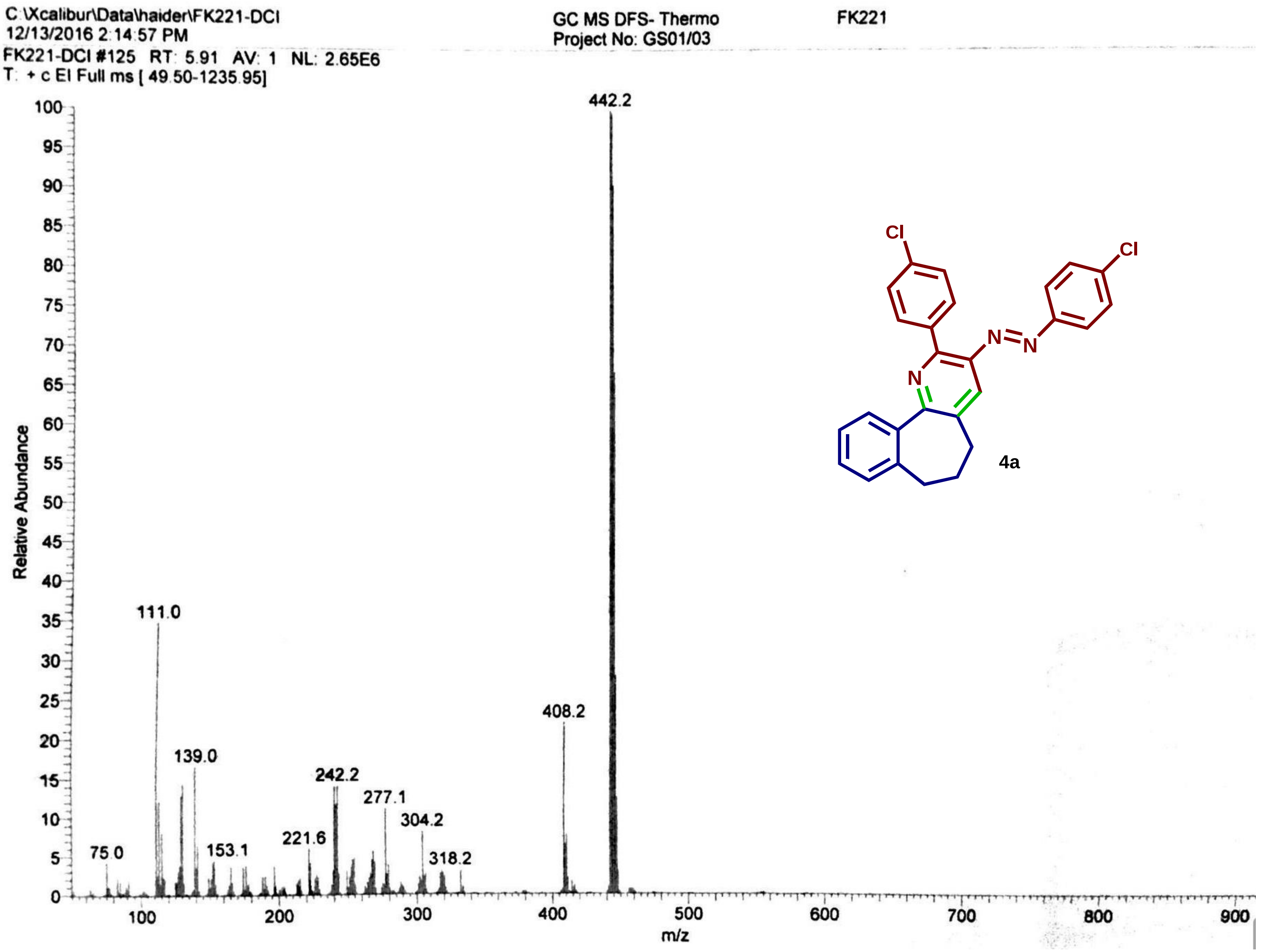

Figure S1. Mass Spectra for compound **4a**.

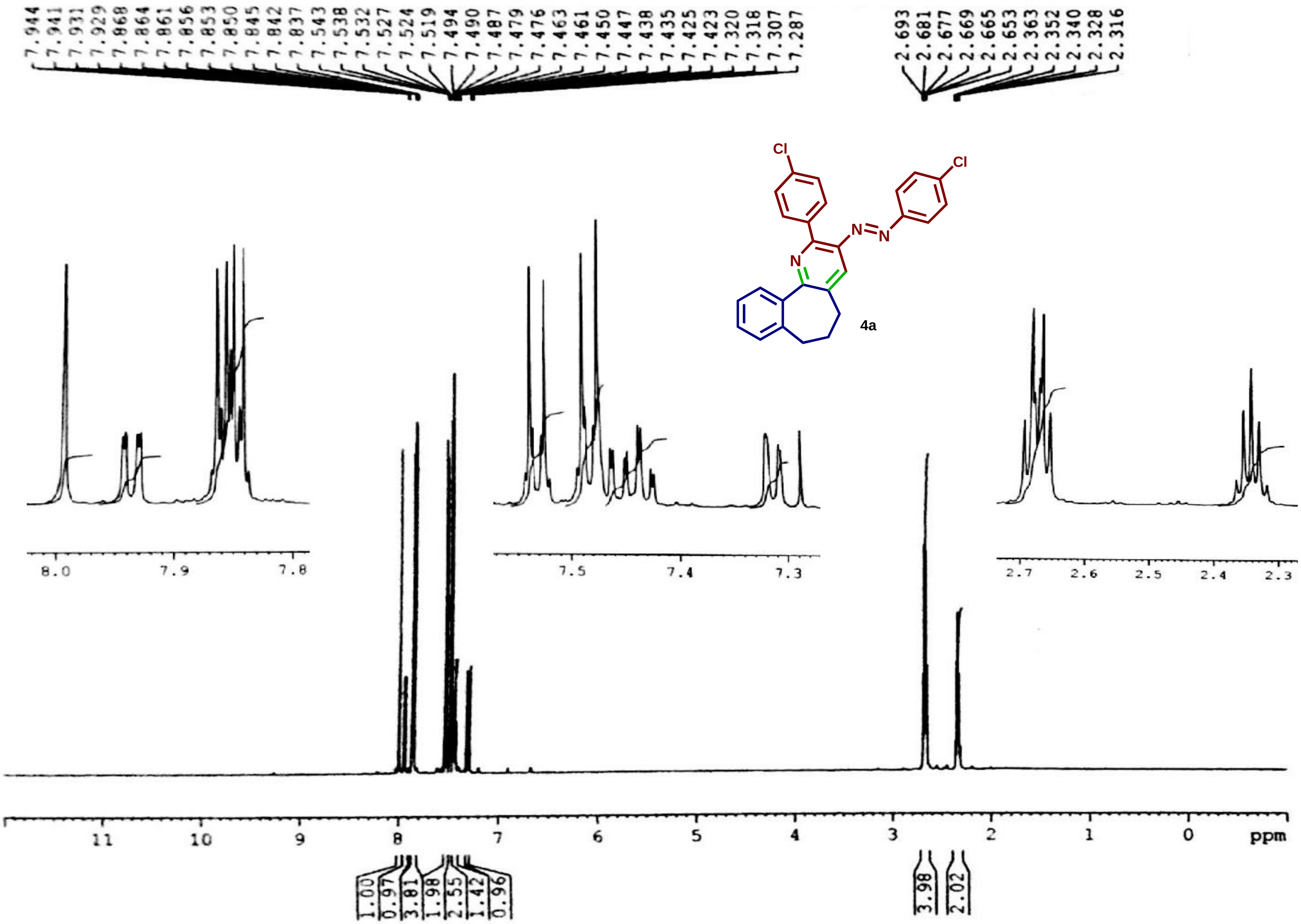

Figure S2. ^{1}H NMR Spectra ($CDCl_3$, 600 MHz) for compound **4a**.

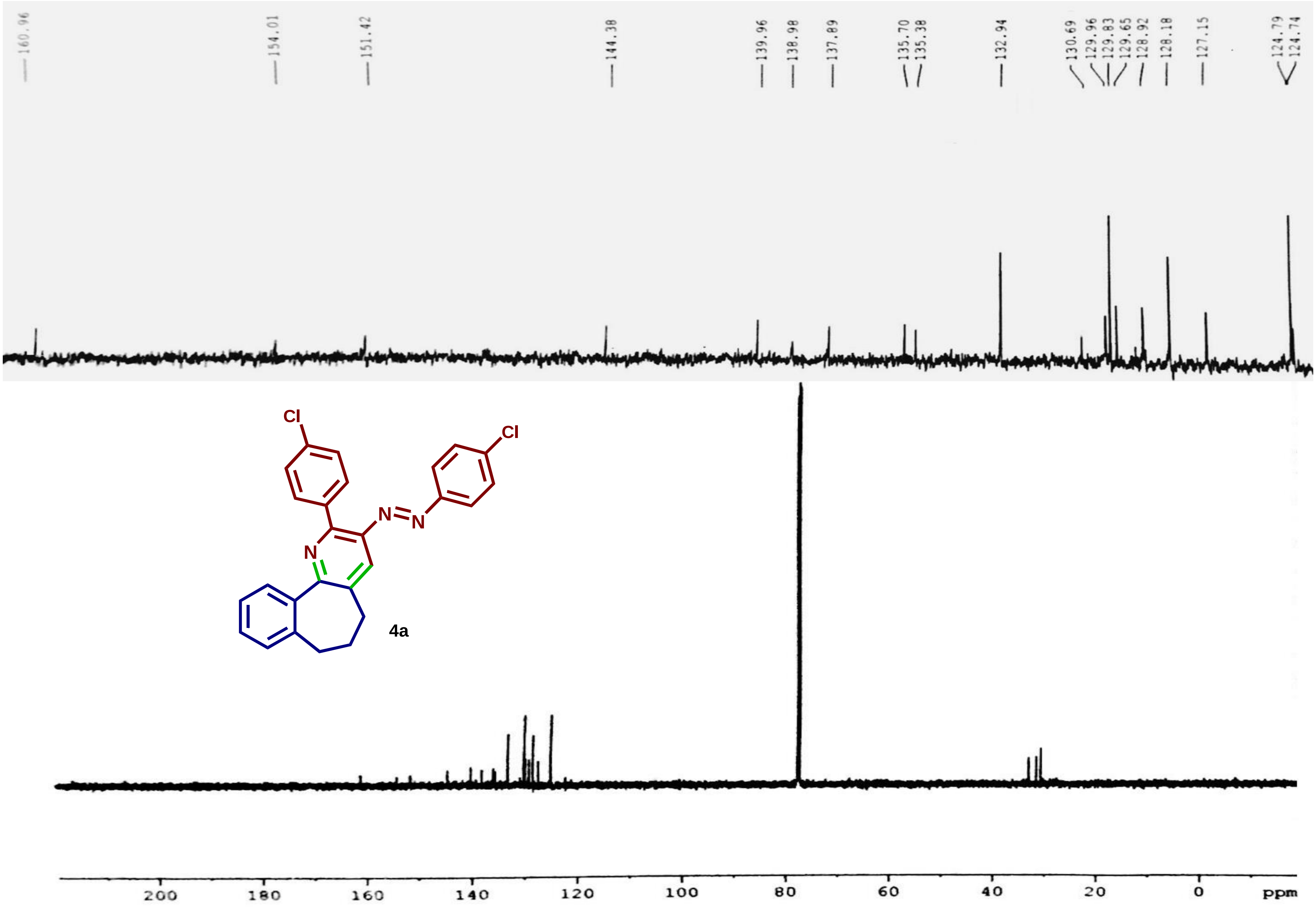

Figure S3. ^{13}C NMR Spectra ($CDCl_3$, 150 MHz) for compound **4a**.

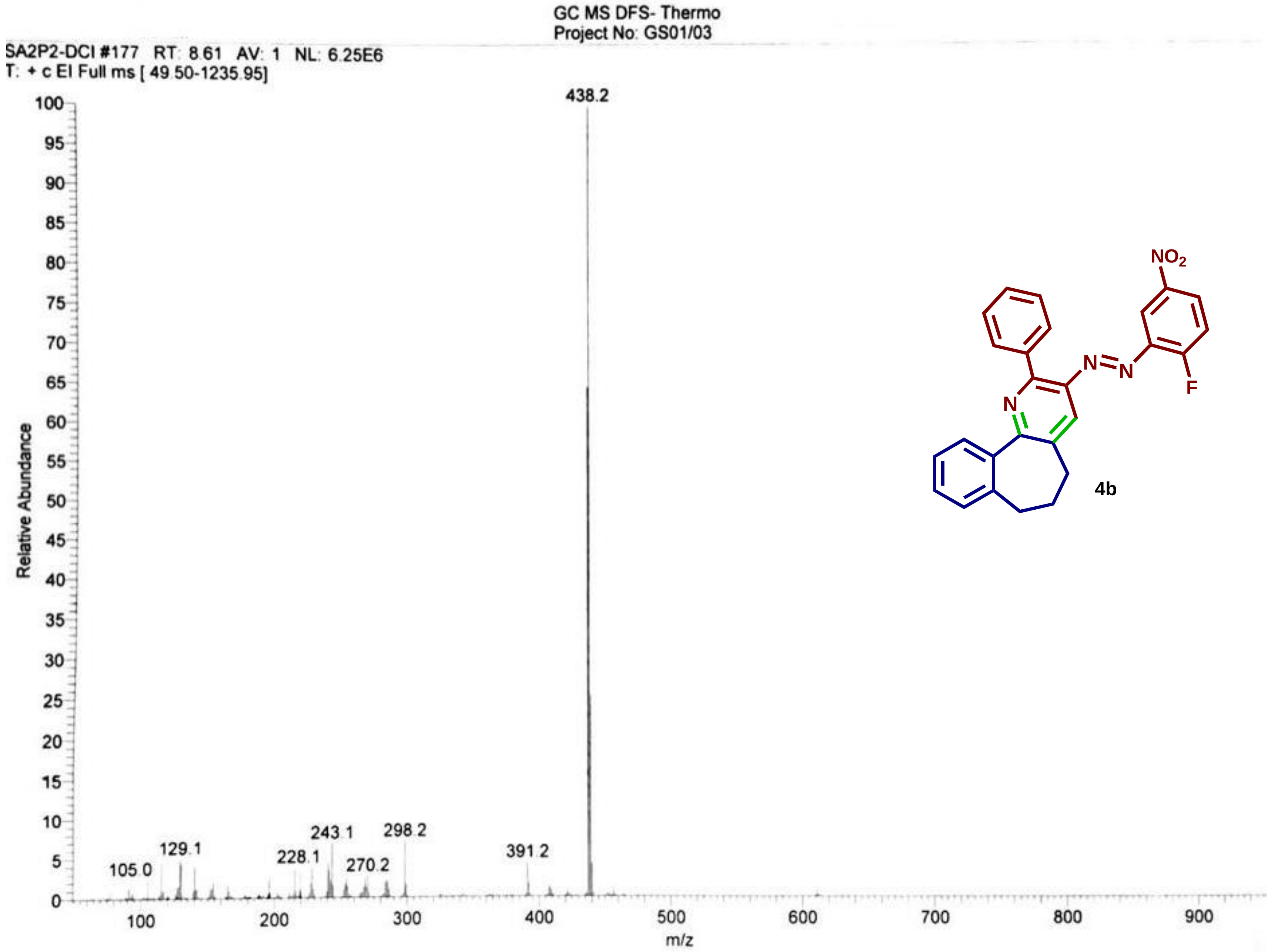

Figure S4. Mass Spectra for compound **4b**.

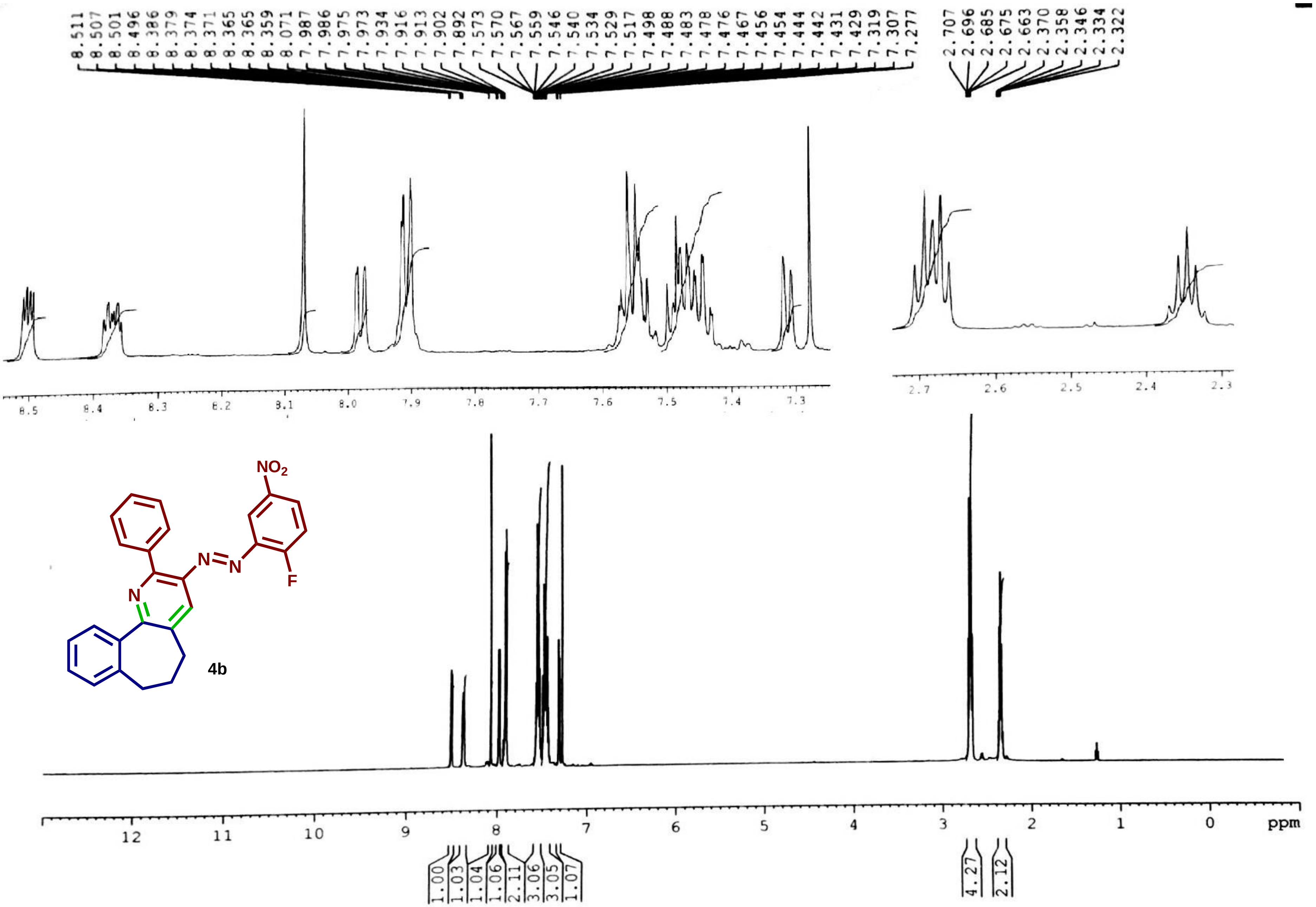

Figure S5. ^{1}H NMR Spectra (600 MHz, $CDCl_3$) for compound **4b**.

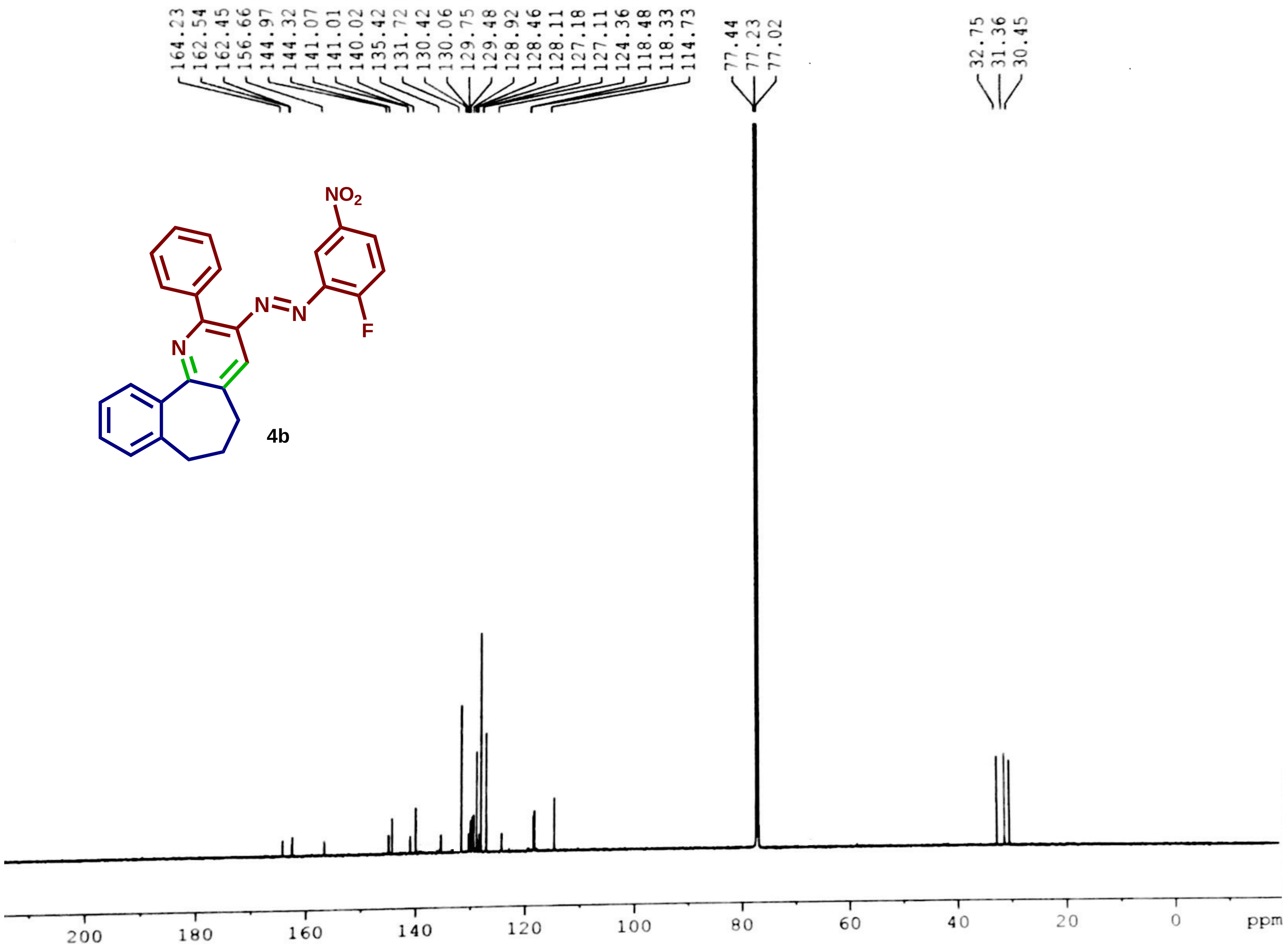

Figure S6. ^{13}C NMR Spectra ($CDCl_3$, 150 MHz) for compound **4b**.

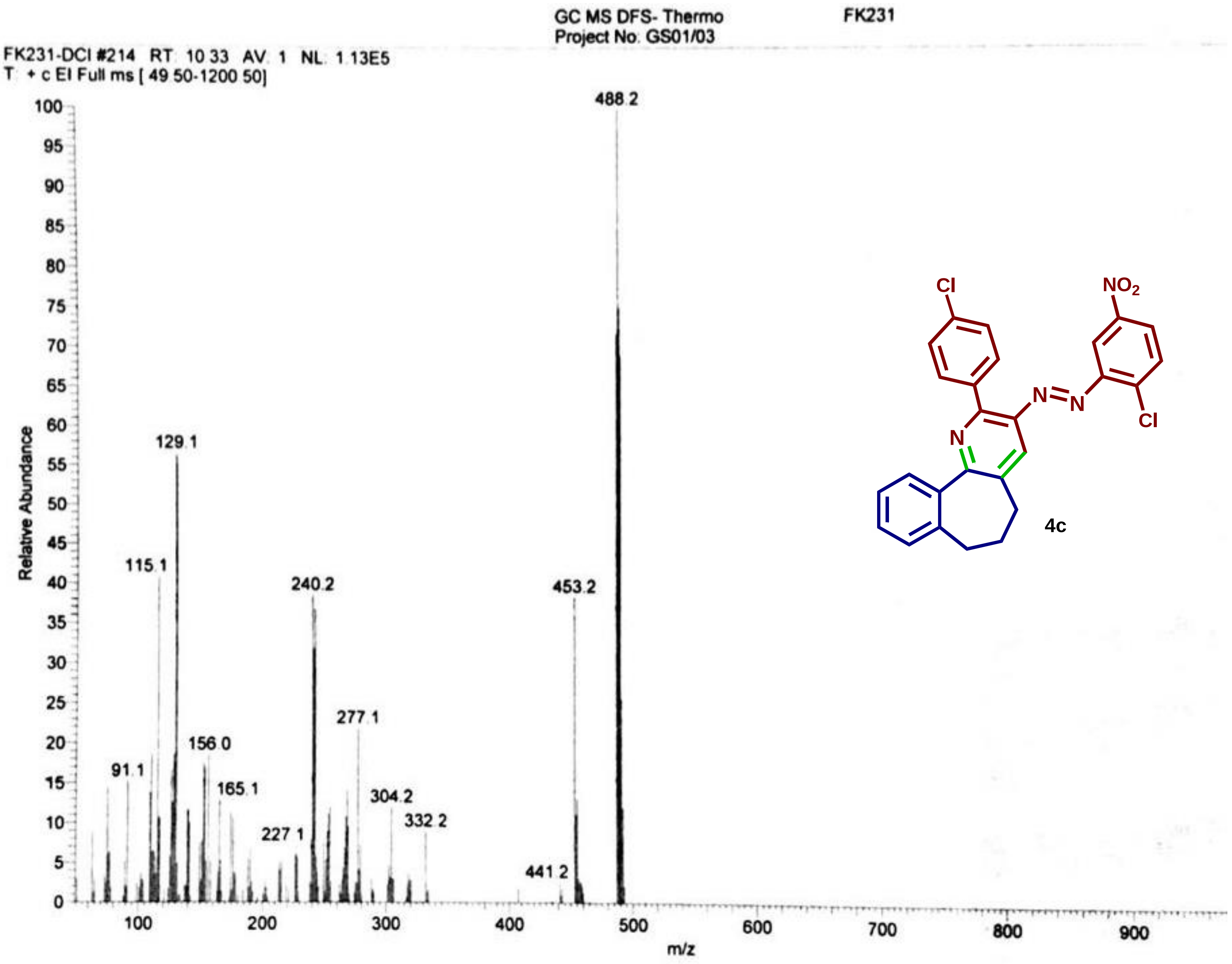

Figure S7. Mass Spectra for compound **4c**.

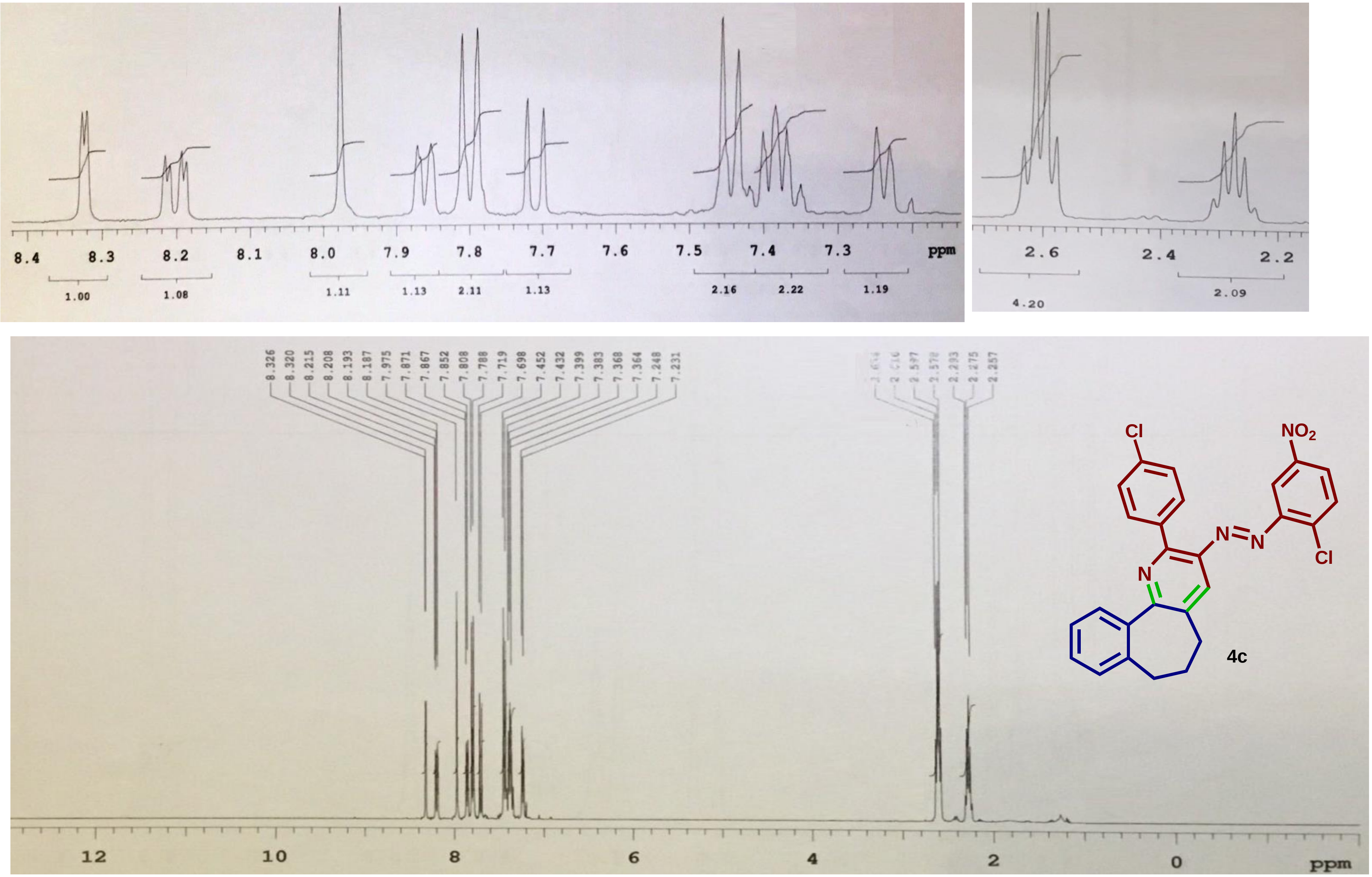

Figure S8. ^{1}H NMR Spectra (400 MHz, $CDCl_3$) for compound **4c**.

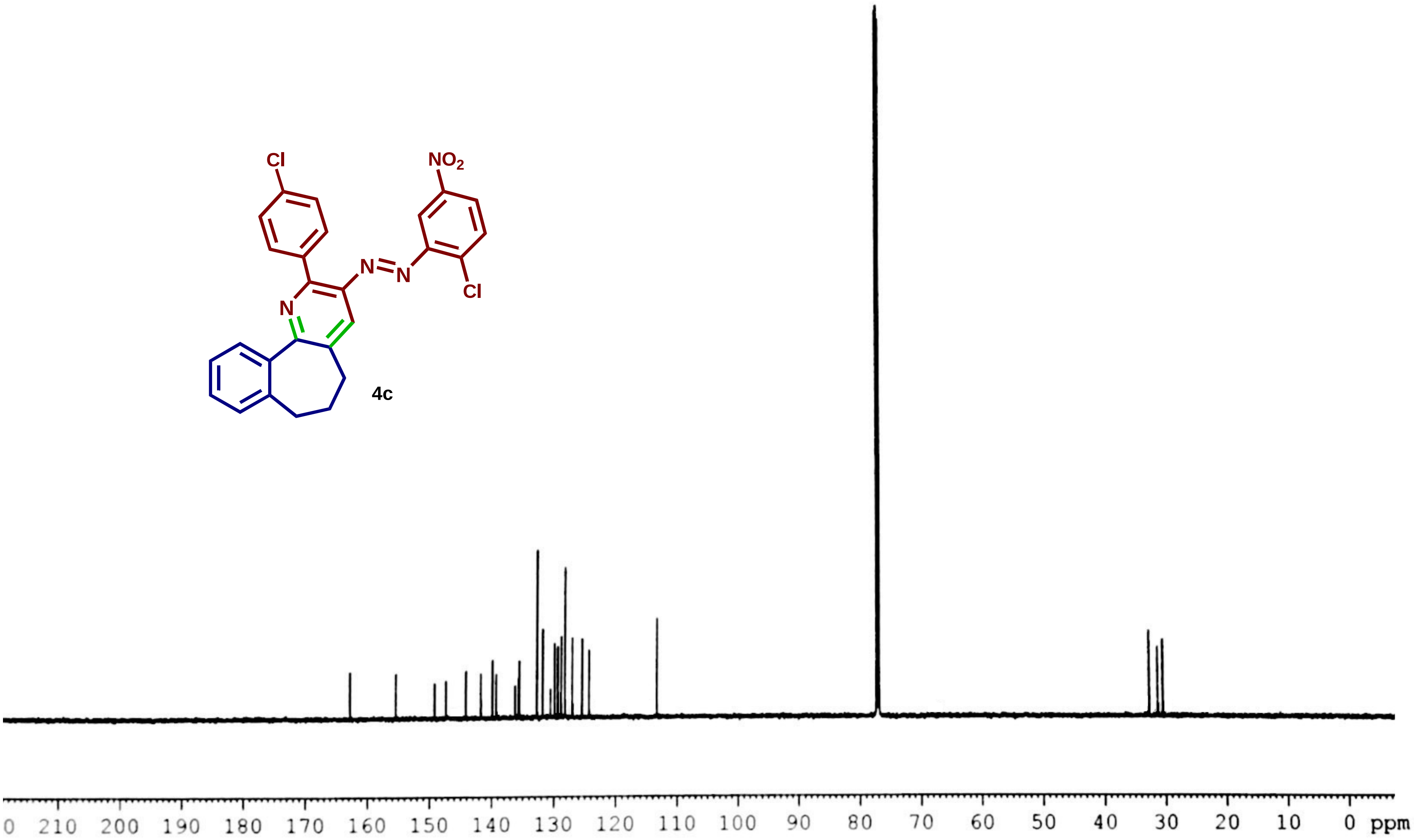

Figure S9. ^{13}C NMR Spectra ($CDCl_3$, 100 MHz) for compound **4c**.

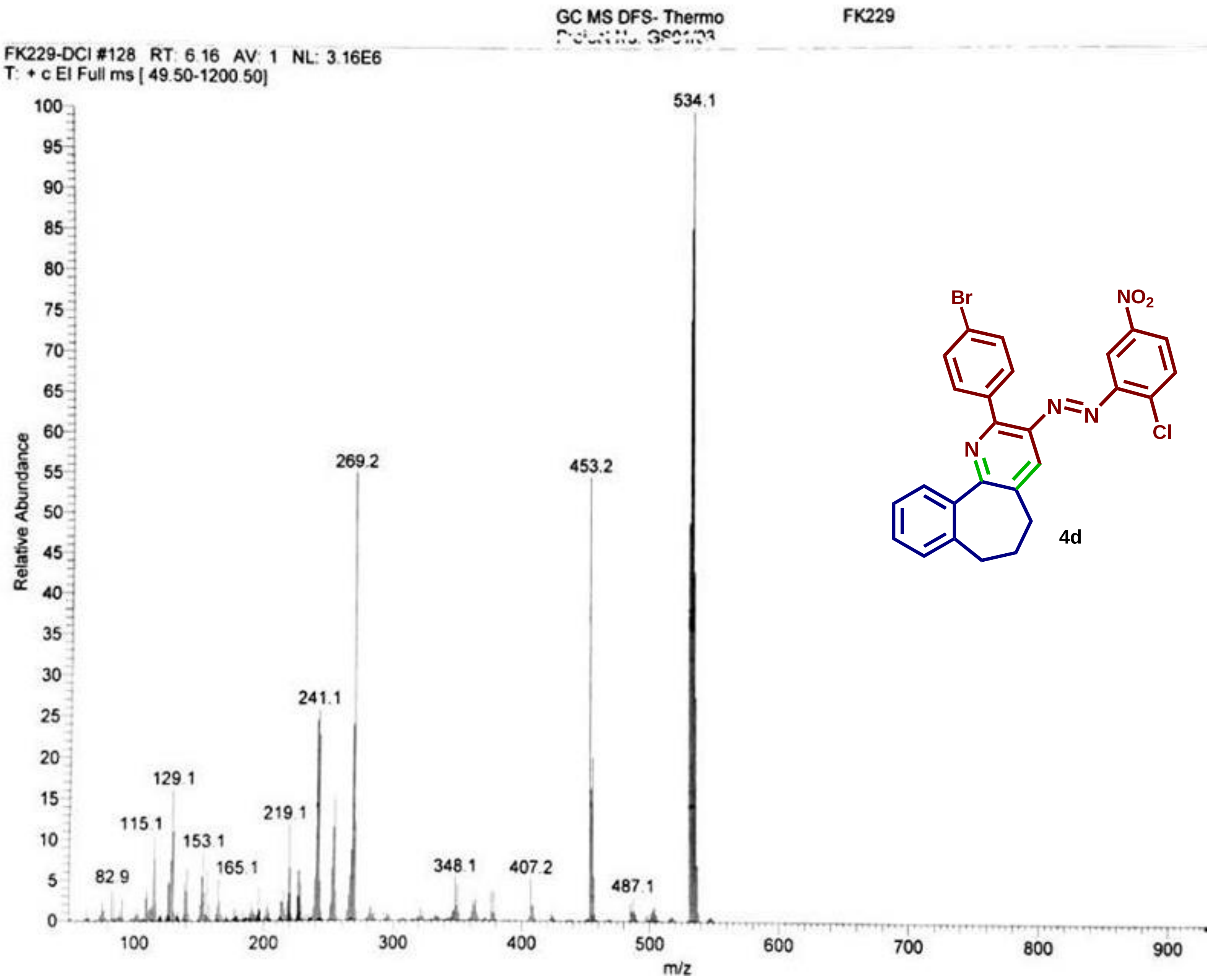

Figure S10. Mass Spectra for compound **4d**.

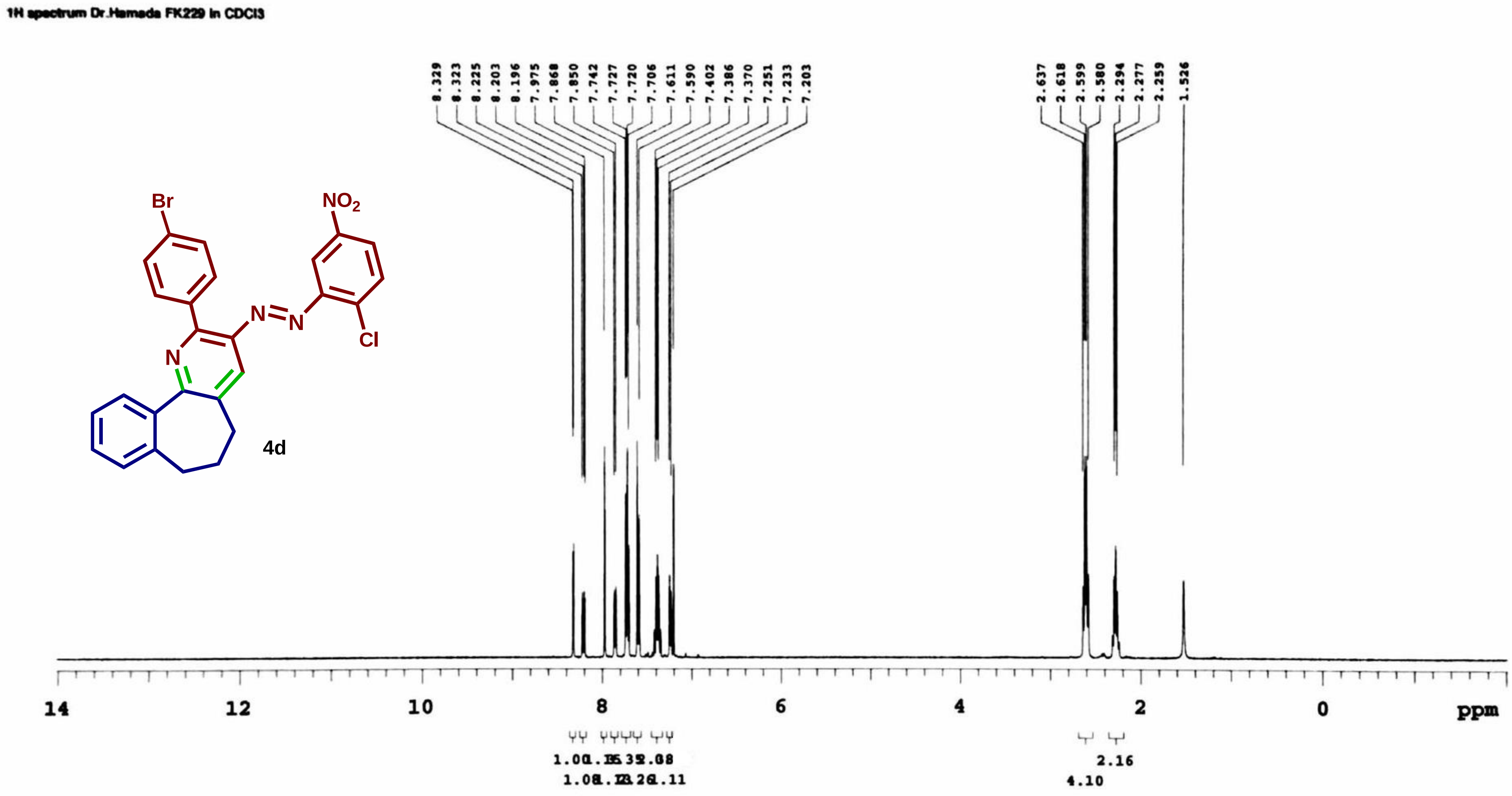

Figure S11. ^{1}H NMR Spectra ($CDCl_3$, 400 MHz) for compound **4d.**

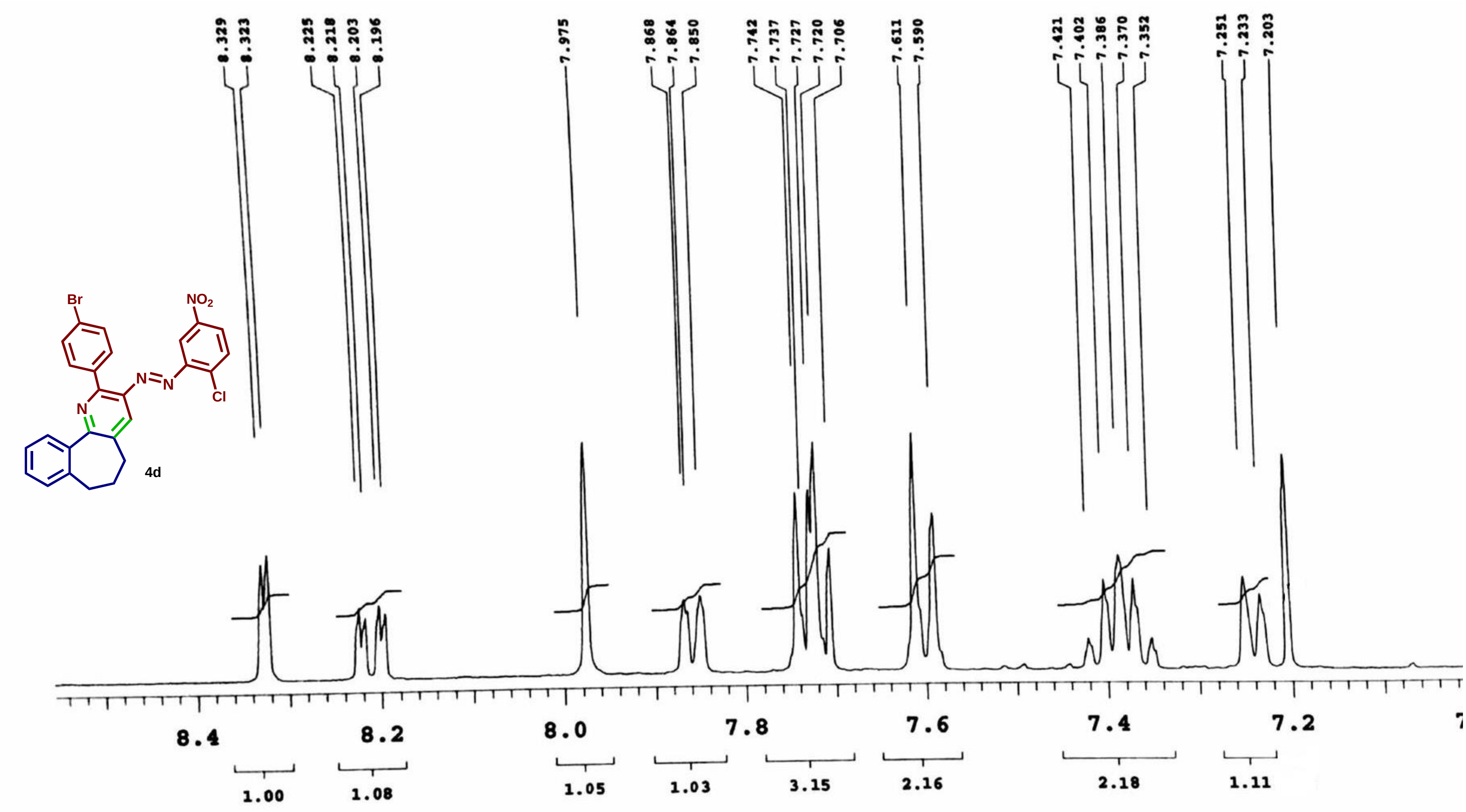

Figure S12. ^{1}H NMR Spectra ($CDCl_3$, 400 MHz) for compound **4d.**

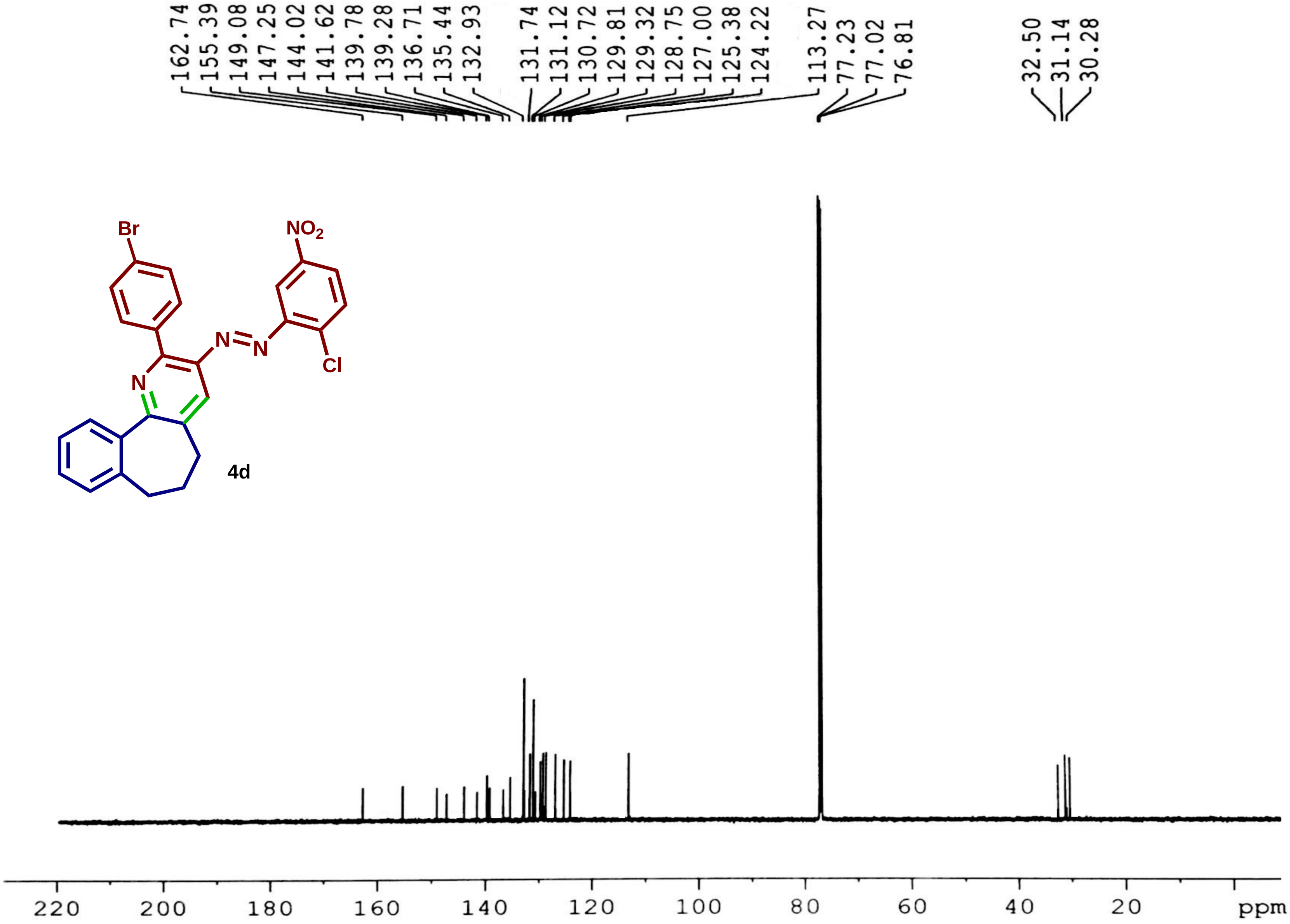

Figure S13. ^{13}C NMR Spectra ($CDCl_3$, 100 MHz) for compound **4d.**

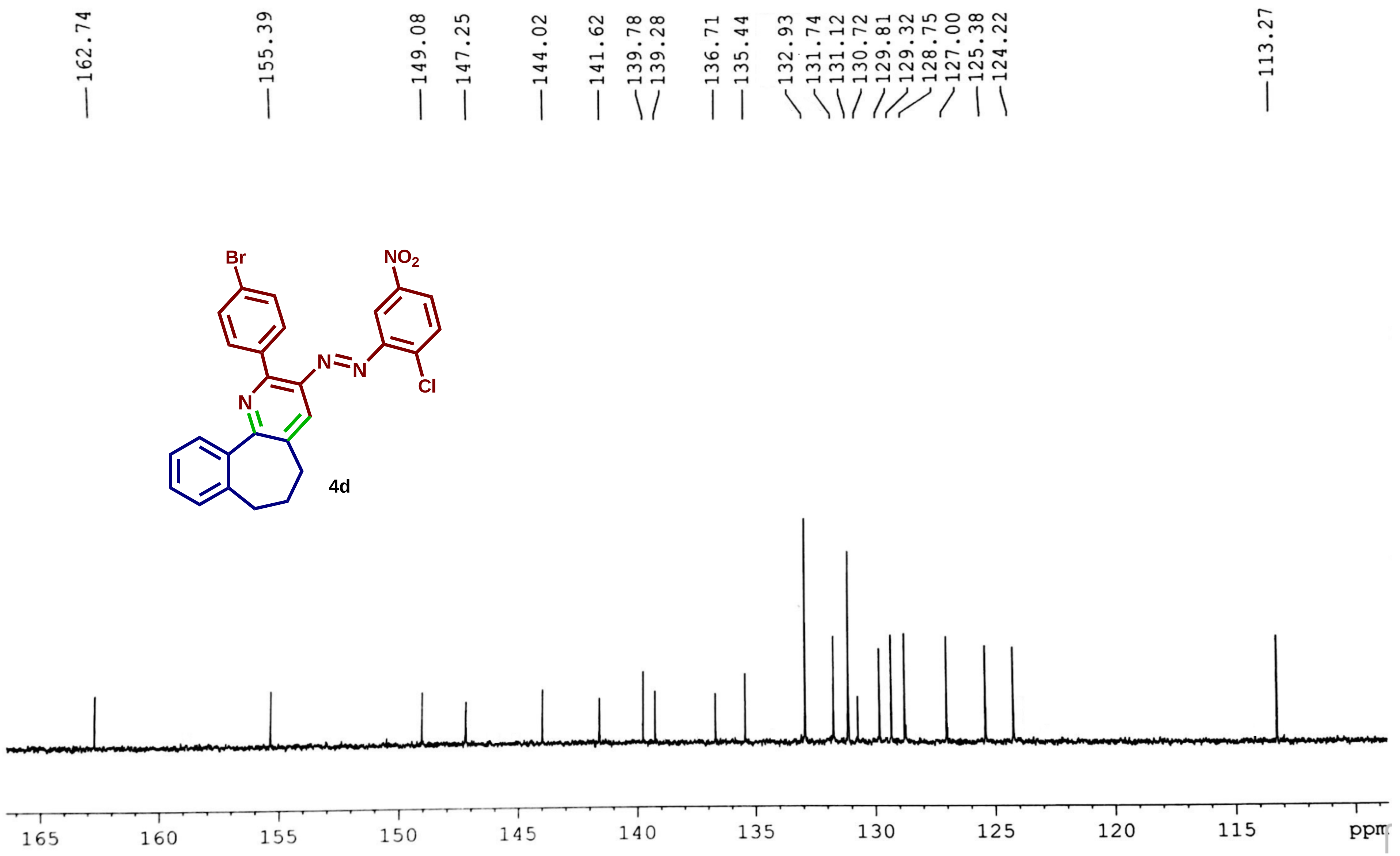

Figure S14. ^{13}C NMR Spectra ($CDCl_3$, 100 MHz) for compound **4d.**

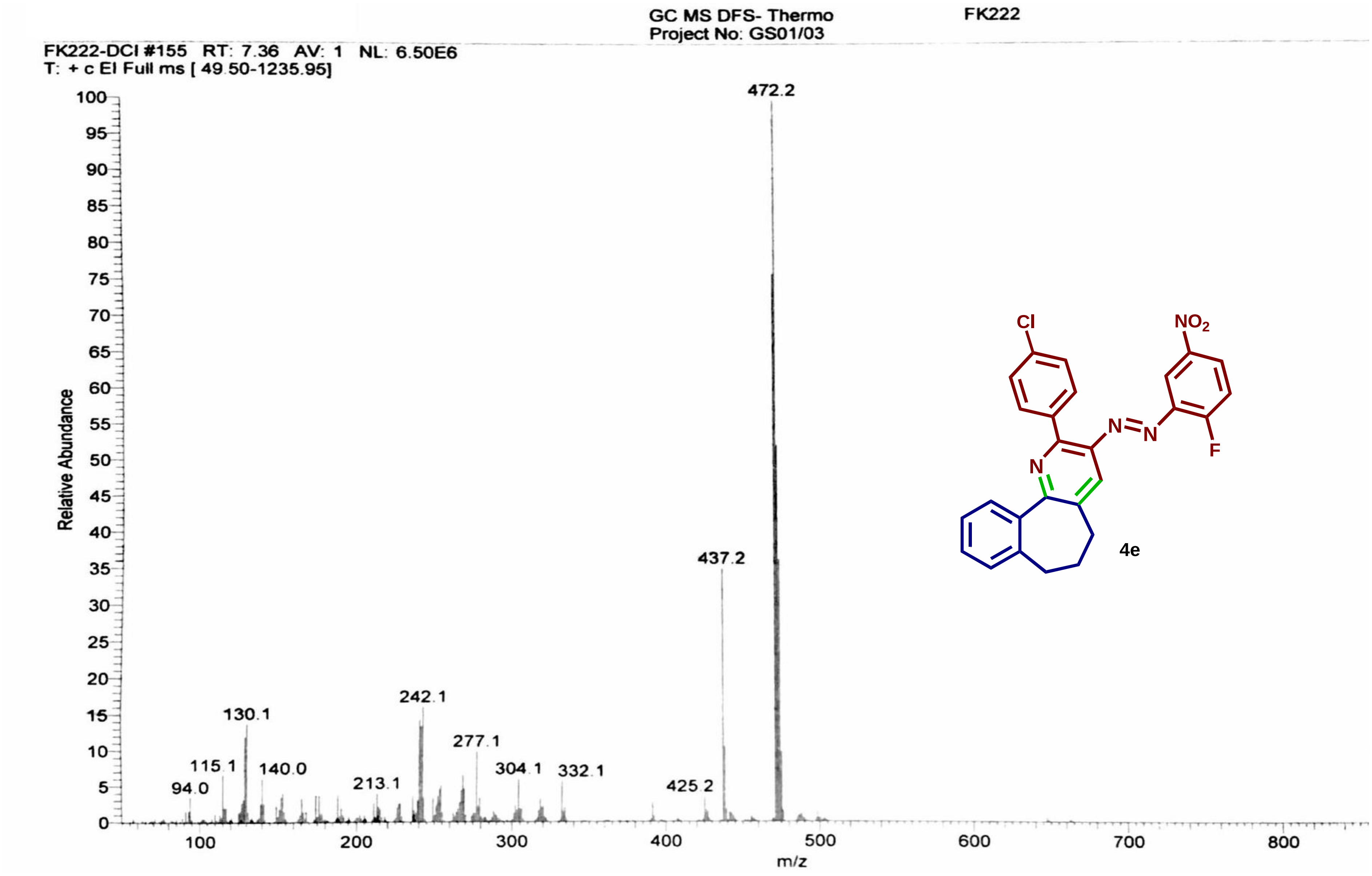

Figure S15. Mass Spectra for compound **4e**.

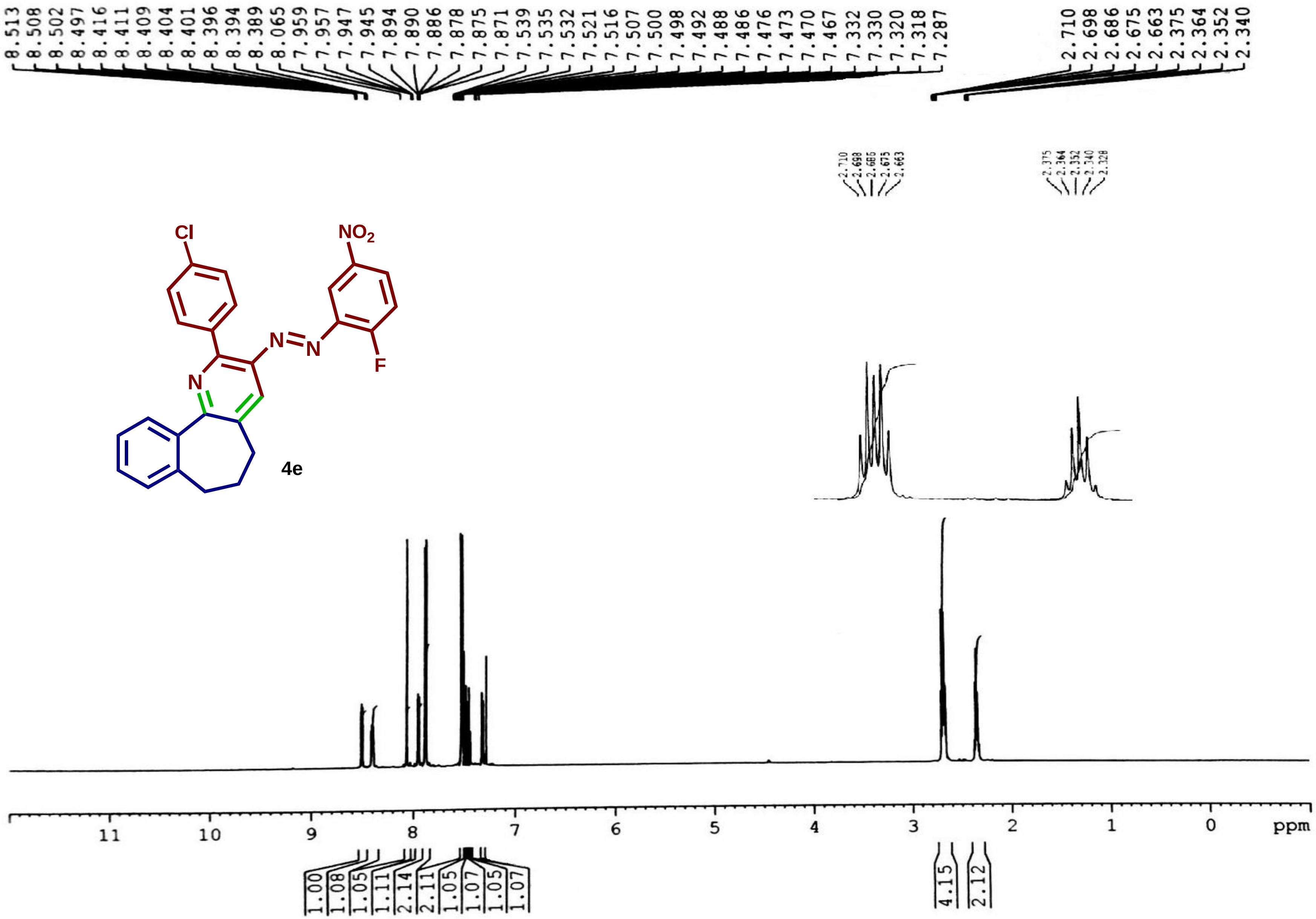

Figure S16. ^{1}H NMR Spectra (DMSO-d_6, 600 MHz) for compound **4e**.

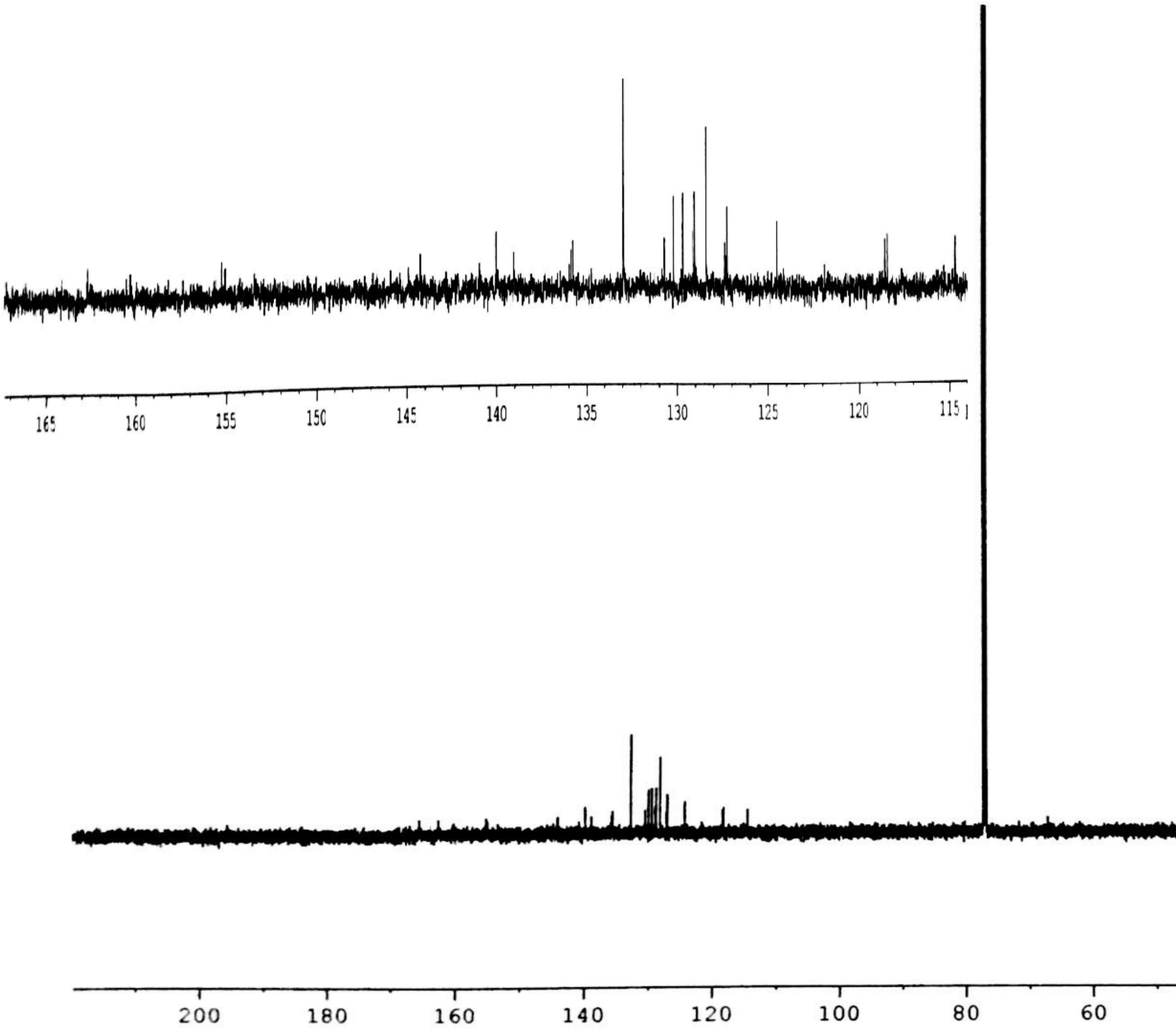

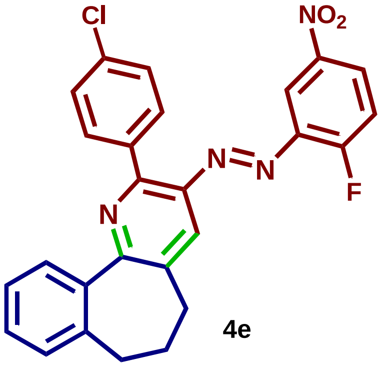

4e

Figure S17. ^{13}C NMR Spectra (DMSO-d_6, 150 MHz) for compound **4e**.

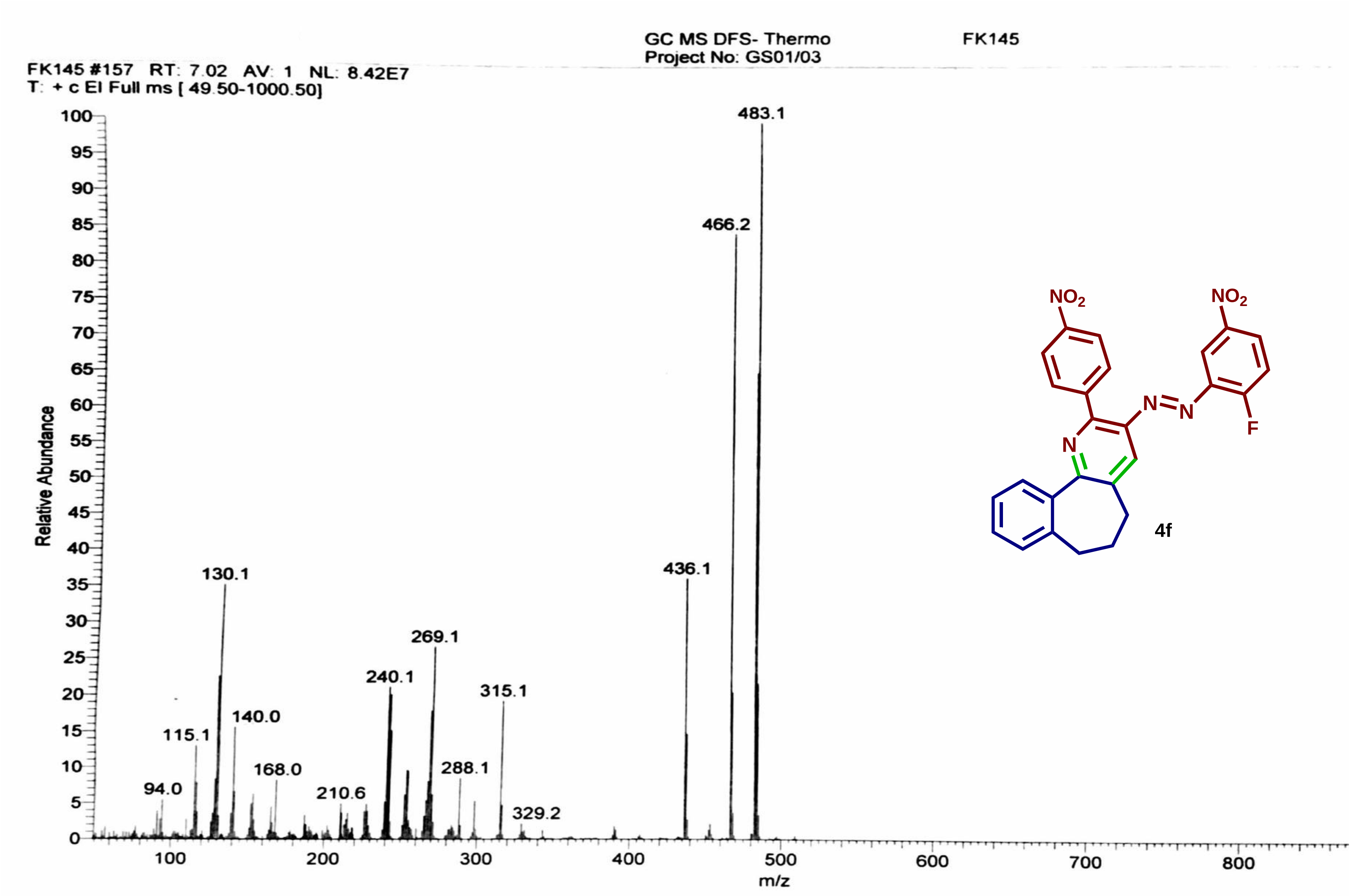

Figure S18. Mass Spectra for compound **4f**.

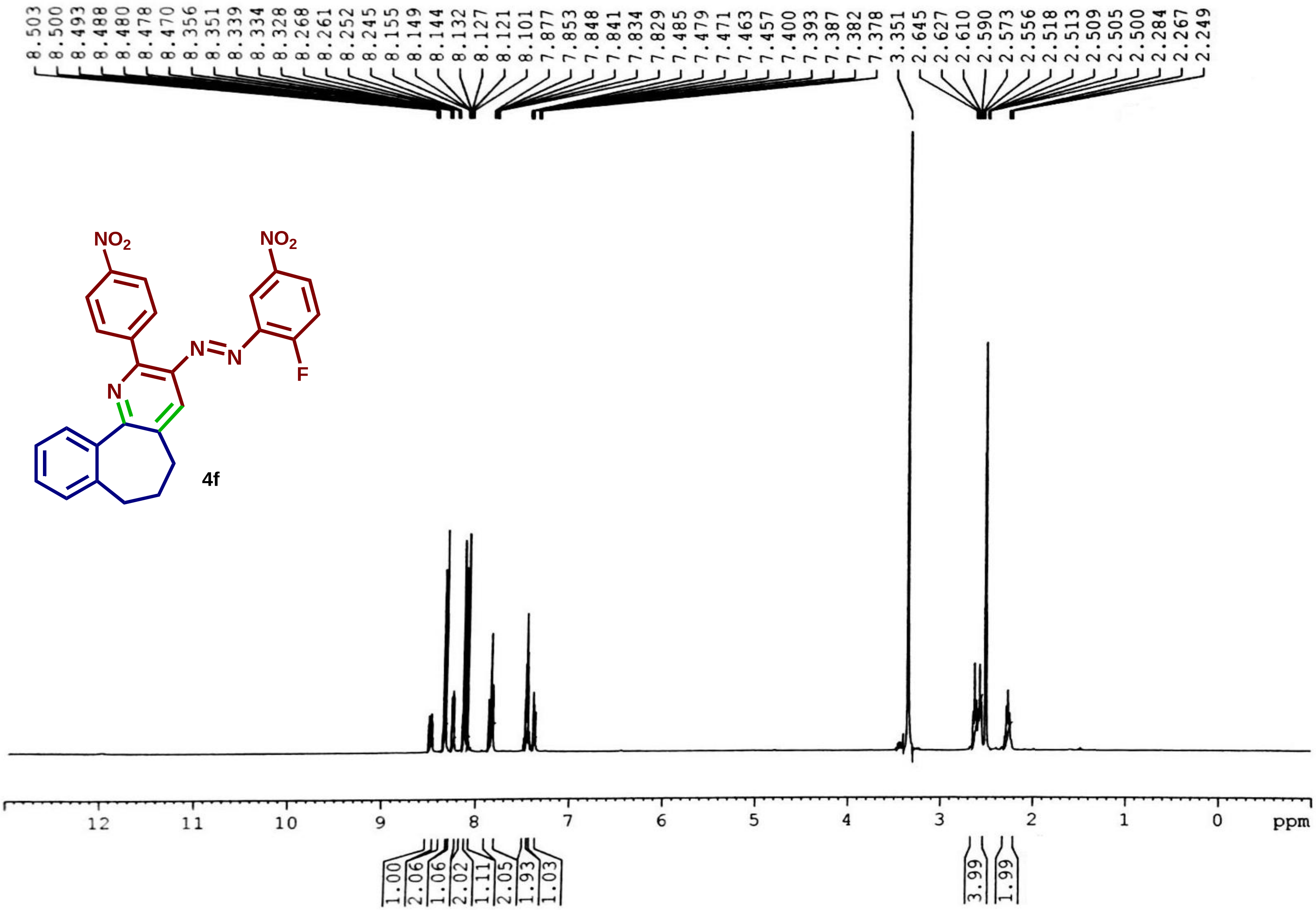

Figure S19. ^{1}H NMR Spectra (DMSO-d_6, 400 MHz) for compound **4f**.

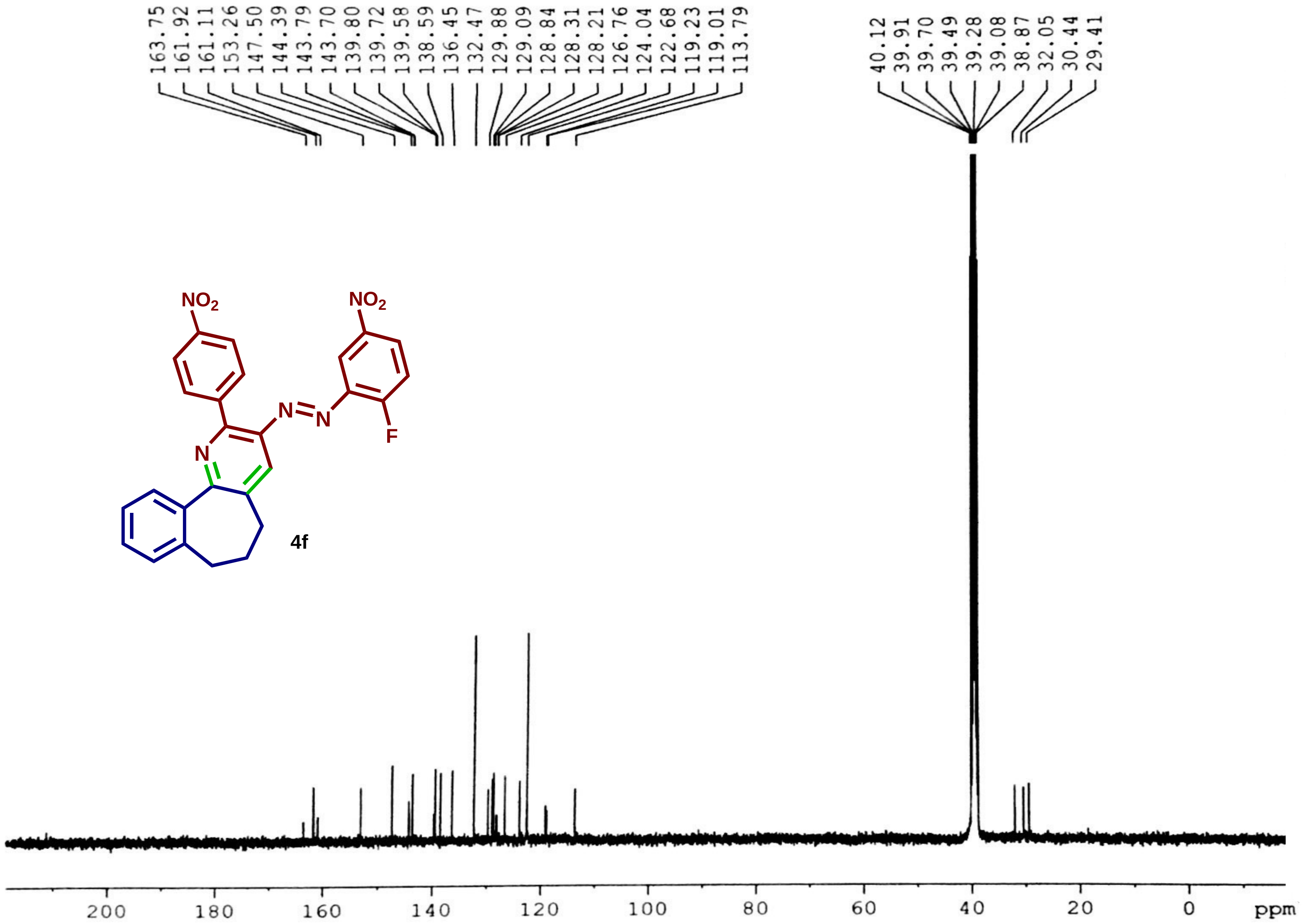

Figure S20. ^{13}C NMR Spectra (DMSO-d_6, 400 MHz) for compound **4f**.

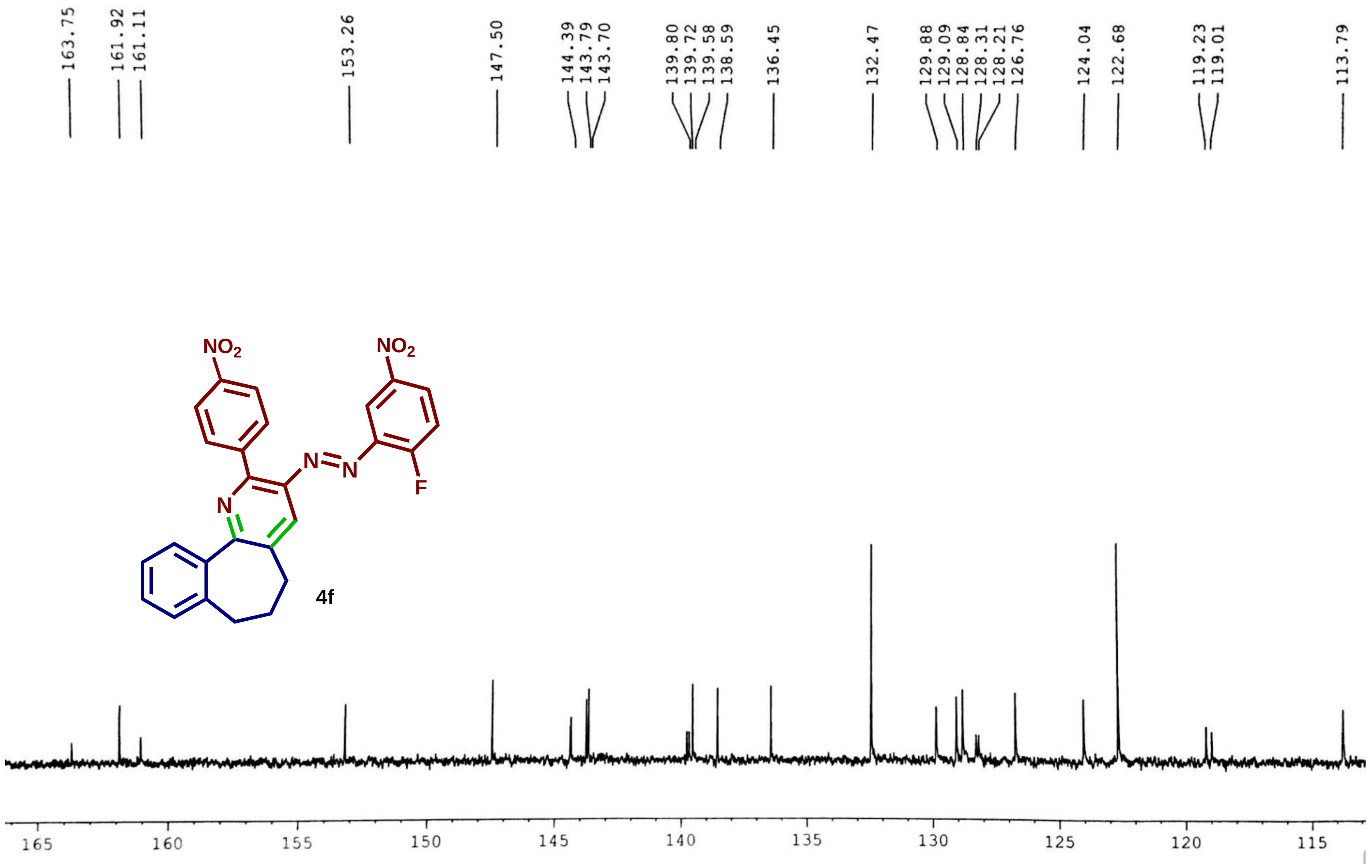

Figure S21. ^{13}C NMR Spectra (DMSO-d_6, 400 MHz) for compound **4f**.

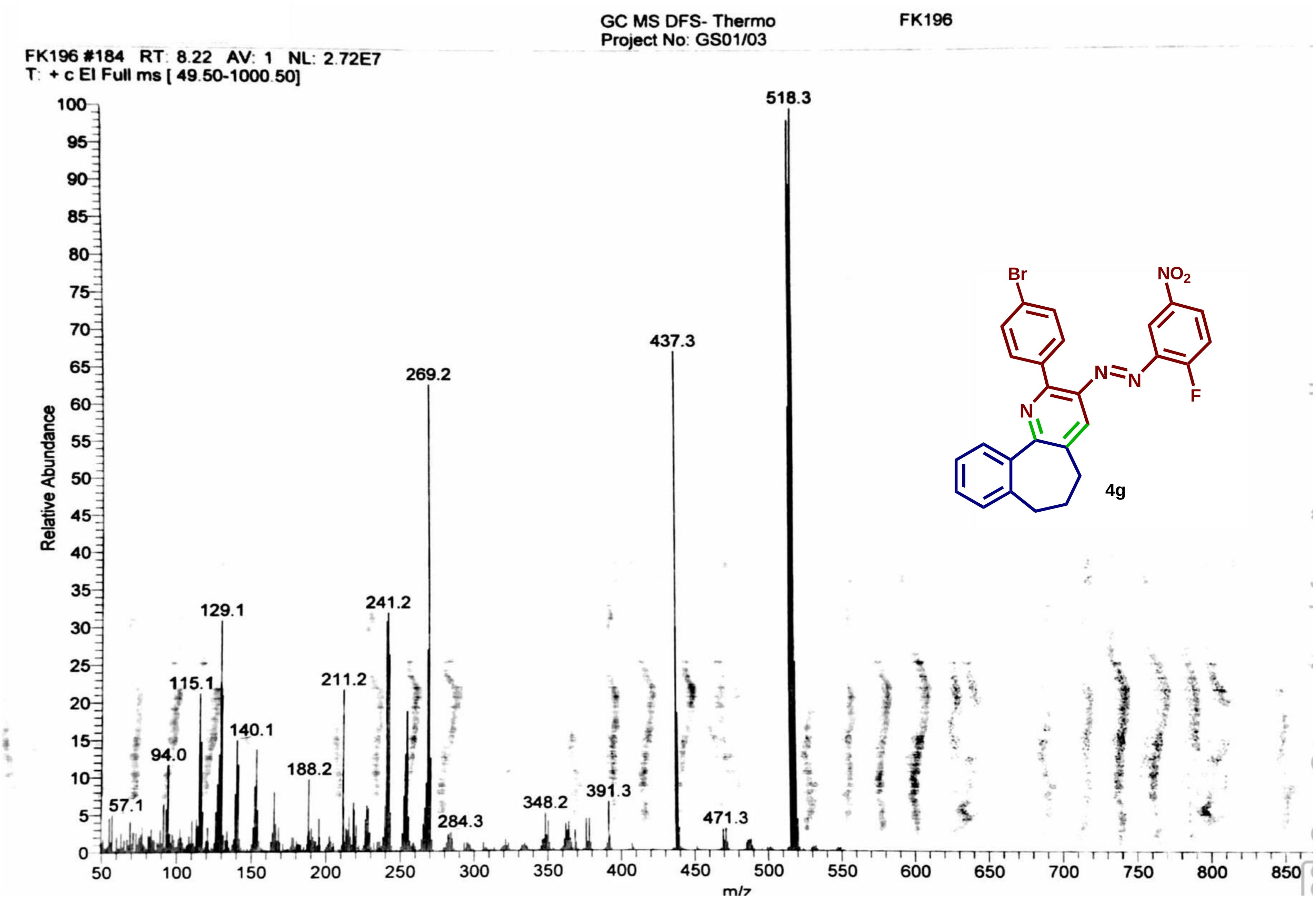

Figure S22. Mass Spectra for compound **4g**.

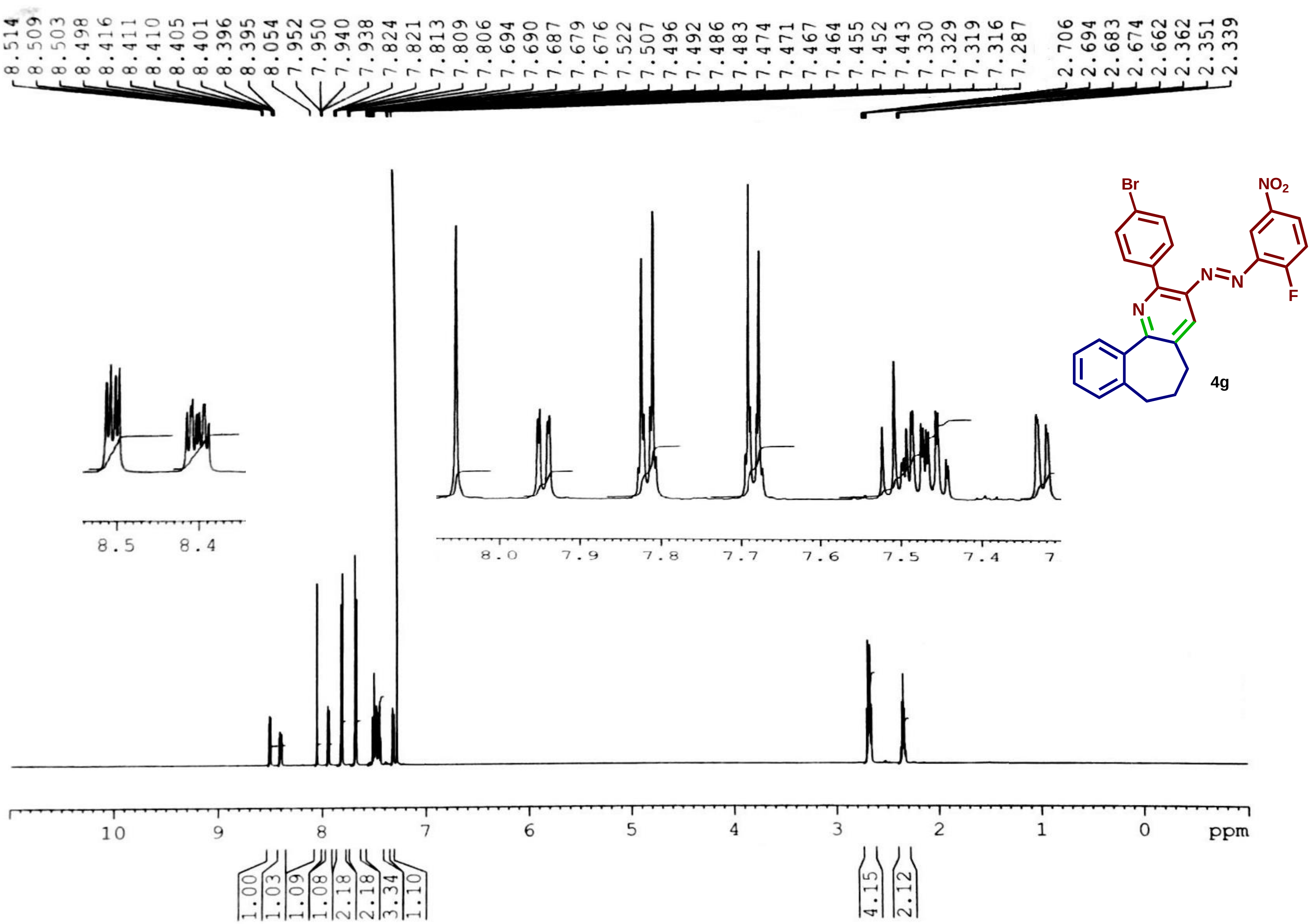

Figure S23. ^{1}H NMR Spectra ($CDCl_3$, 600 MHz) for compound **4g**.

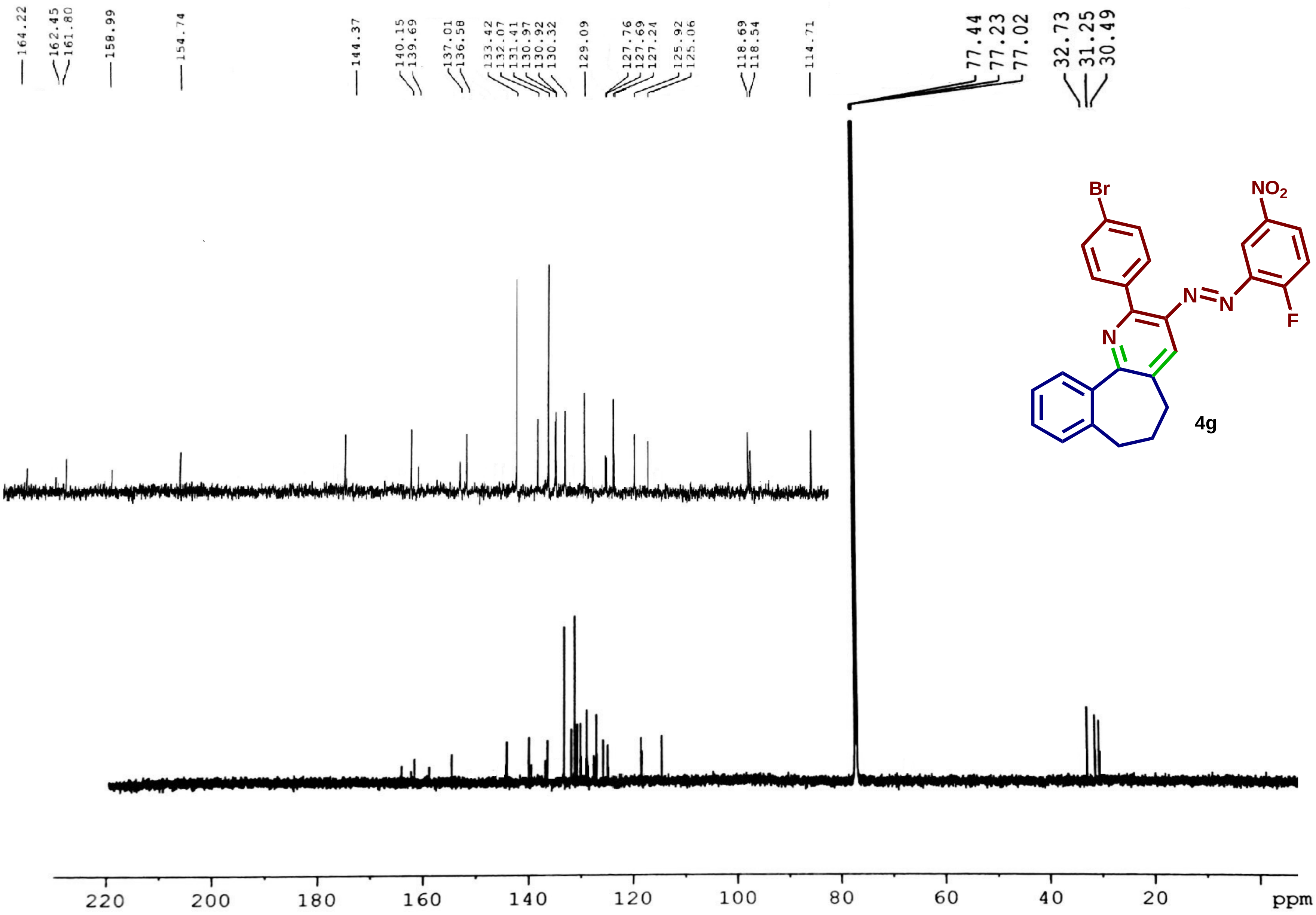

Figure S24. ^{13}C NMR Spectra ($CDCl_3$, 150 MHz) for compound **4g**.

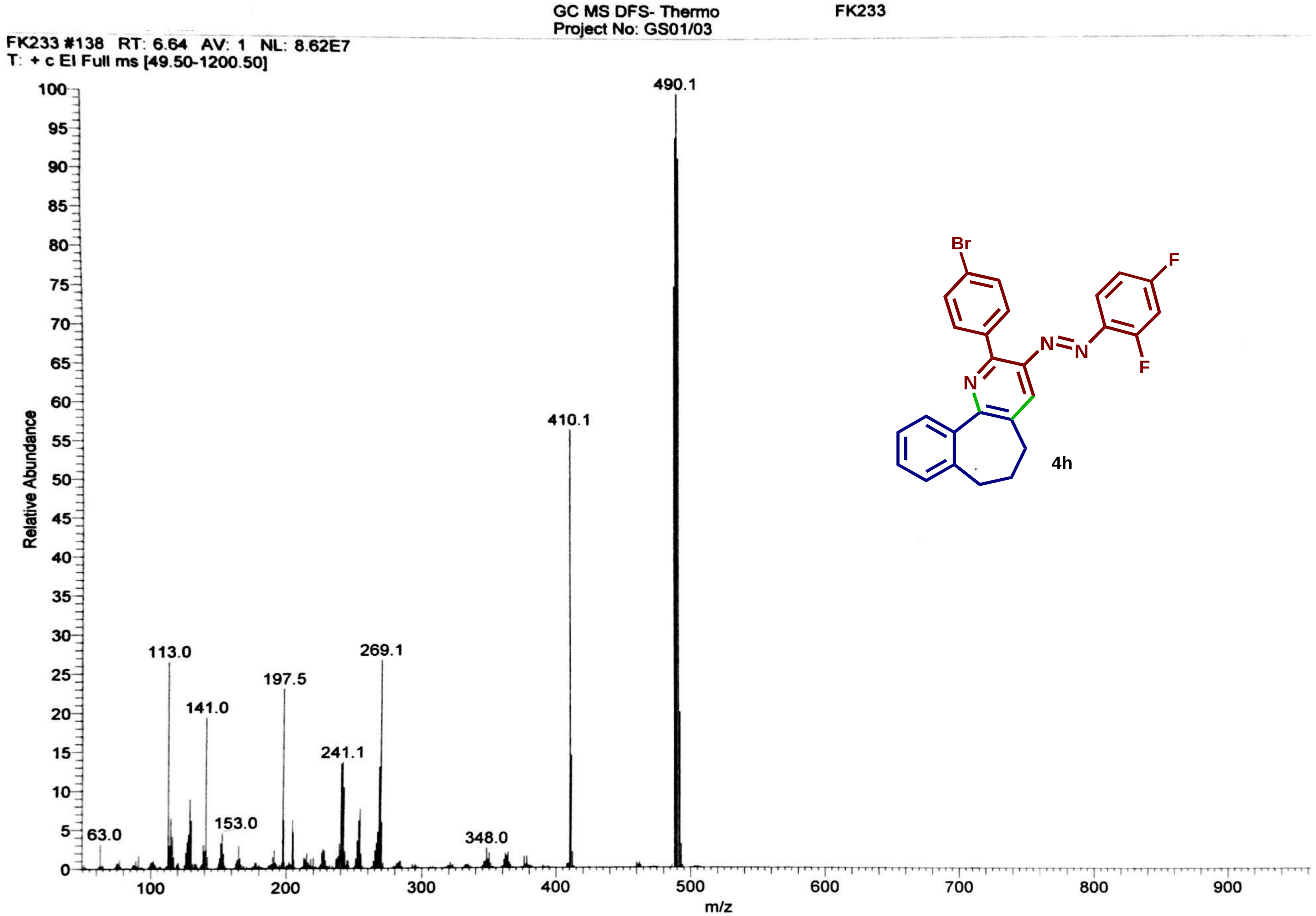

Figure S25. Mass Spectra for compound **4h**.

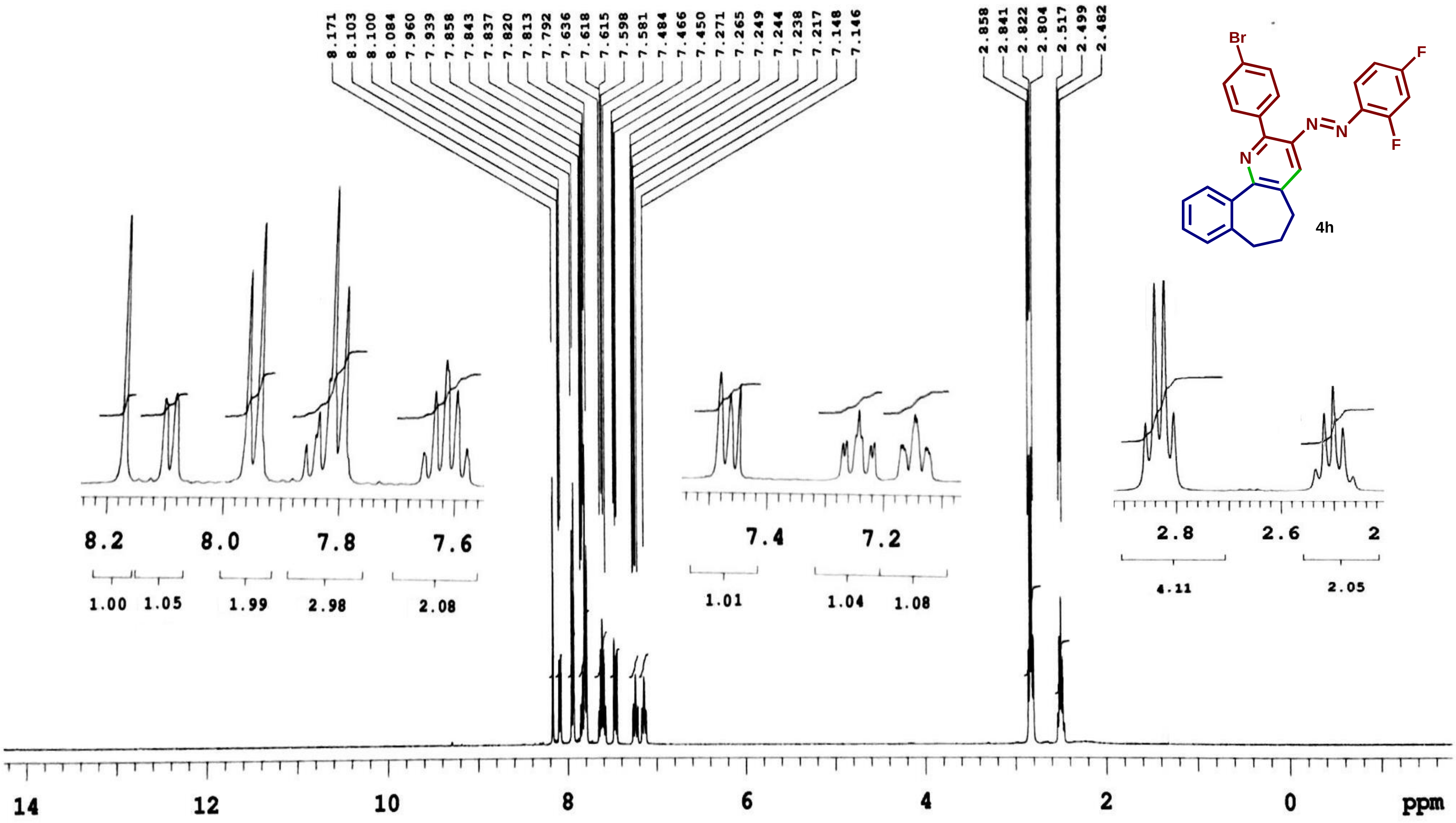

Figure S26. ^{1}H NMR Spectra ($CDCl_3$, 400 MHz) for compound **4h**.

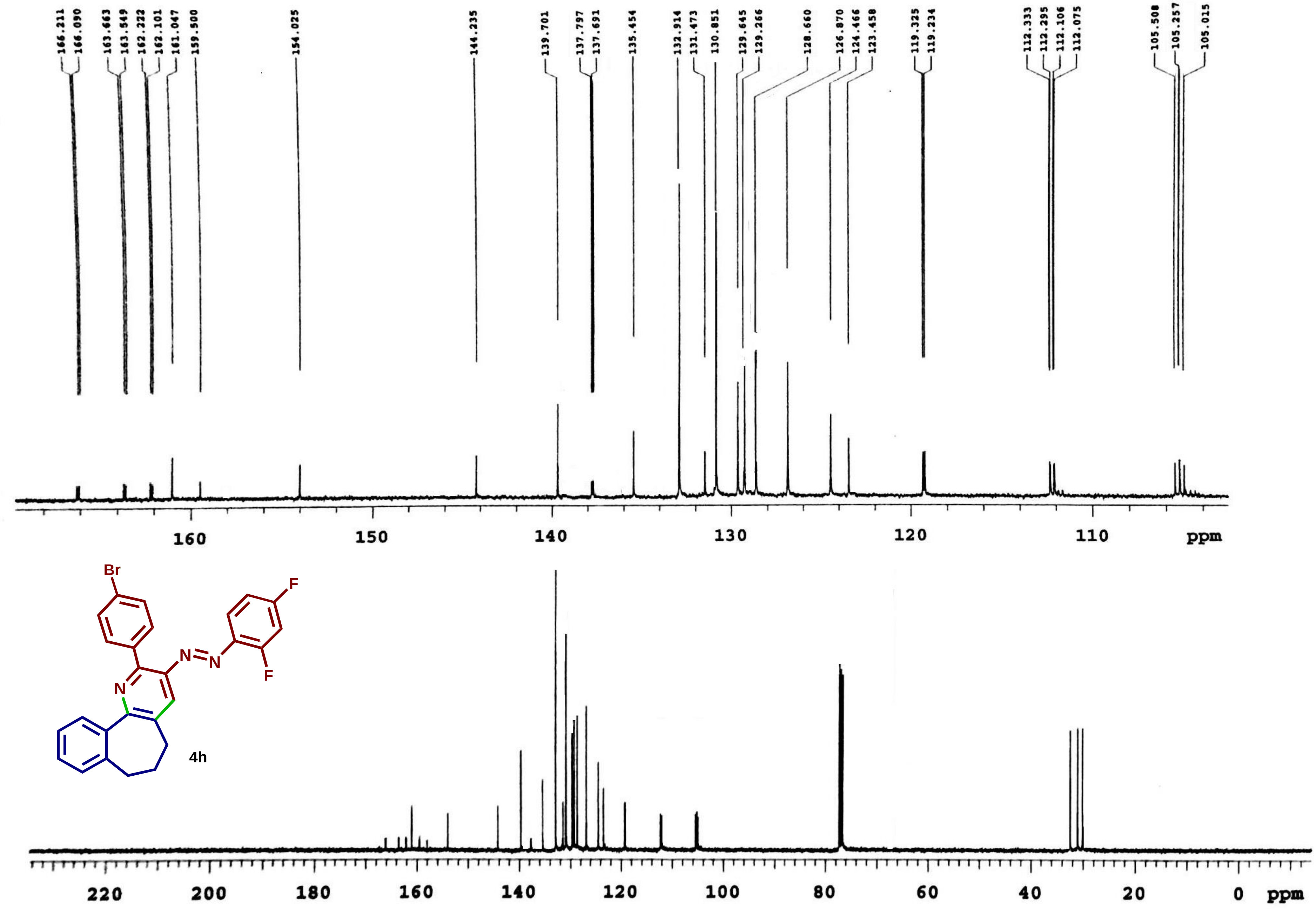

Figure S27. ^{13}C NMR Spectra ($CDCl_3$, 100 MHz) for compound**4h**.

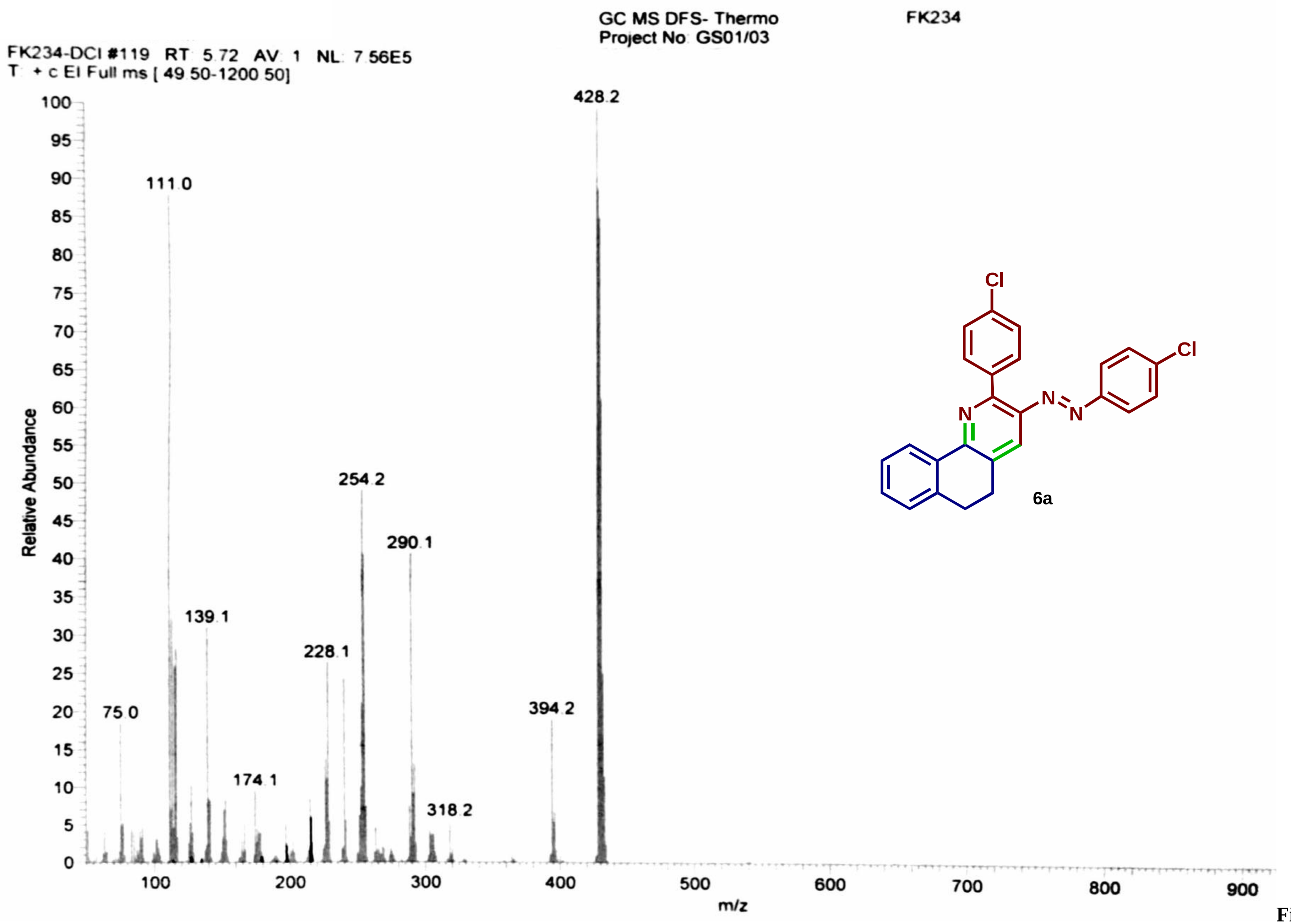

Figure S28. Mass Spectra for compound **6a**.

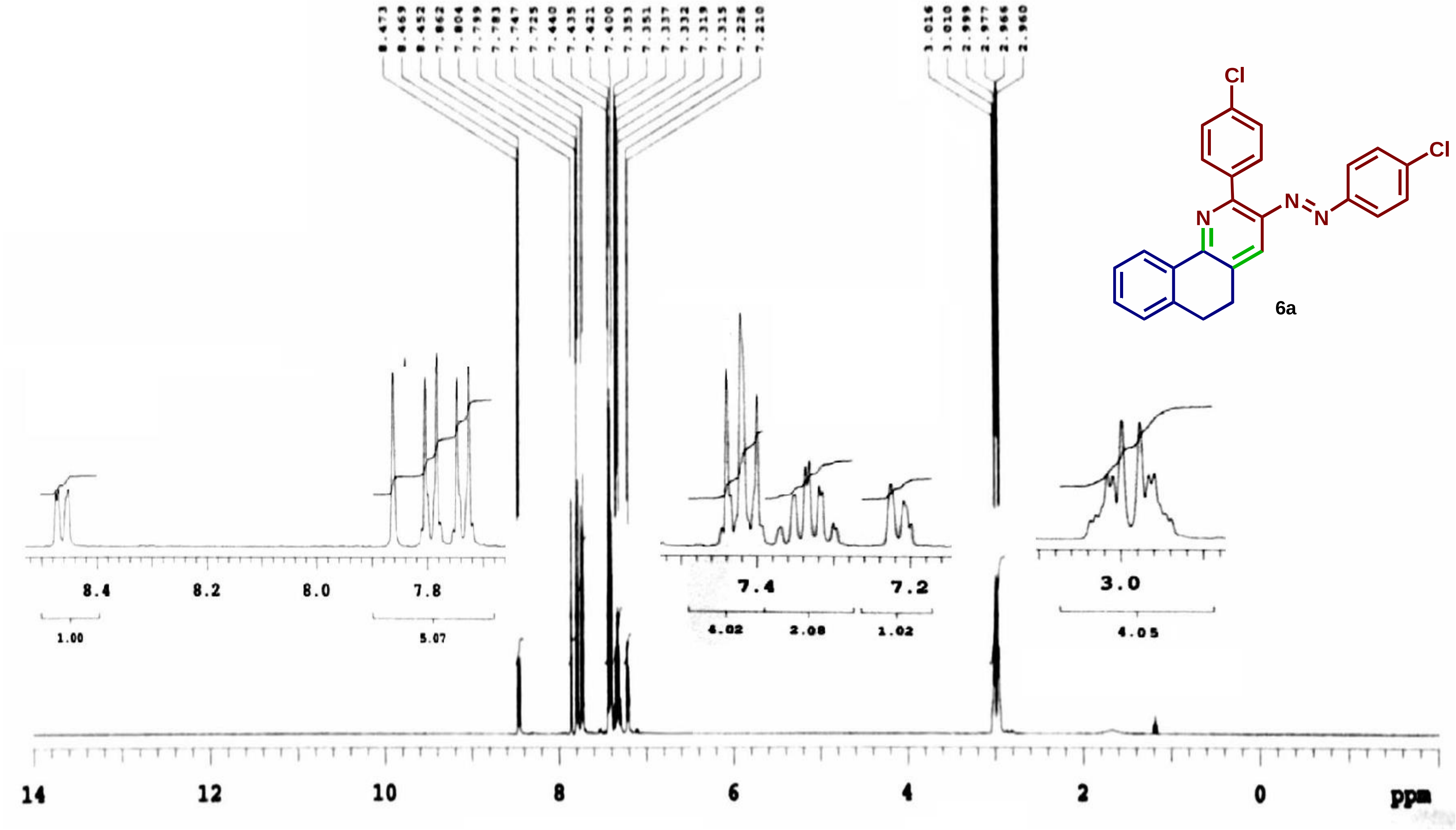

Figure S29. ^{1}H NMR Spectra ($CDCl_3$, 400 MHz) for compound **6a**.

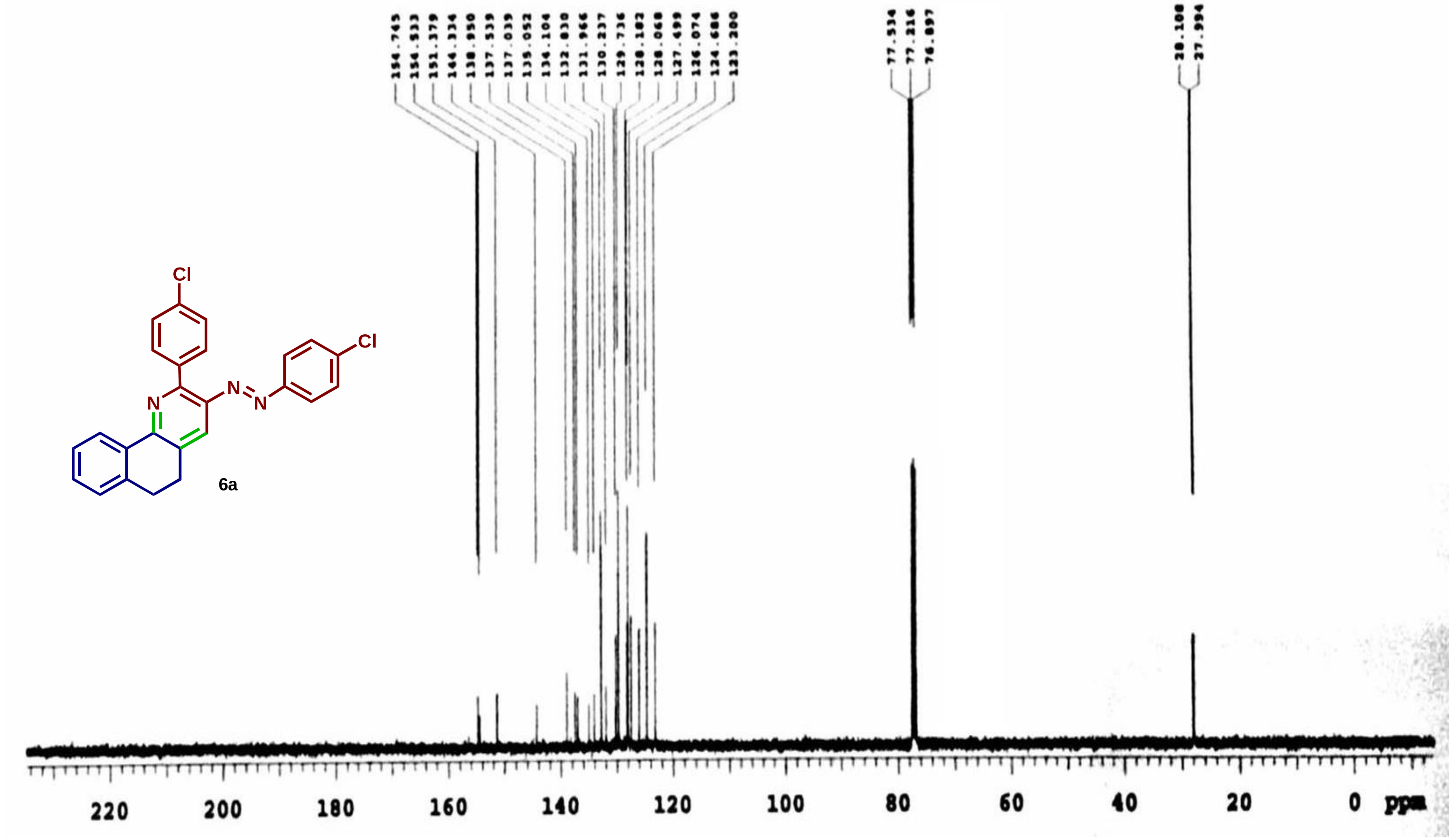

Figure S30. ^{13}C NMR Spectra ($CDCl_3$, 100 MHz) for compound **6a**.

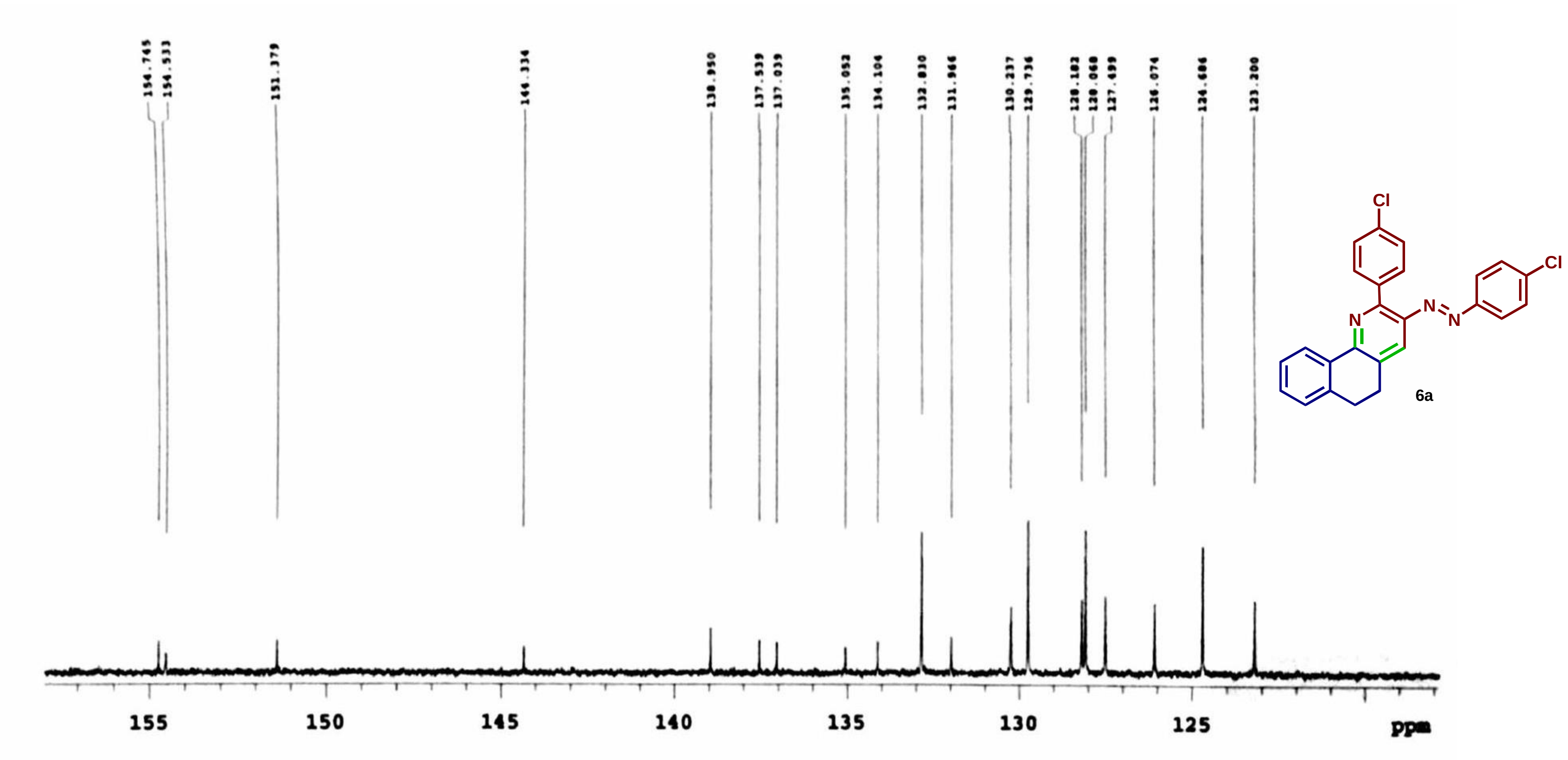

Figure S31. ^{13}C NMR Spectra ($CDCl_3$, 100 MHz) for compound **6a**.

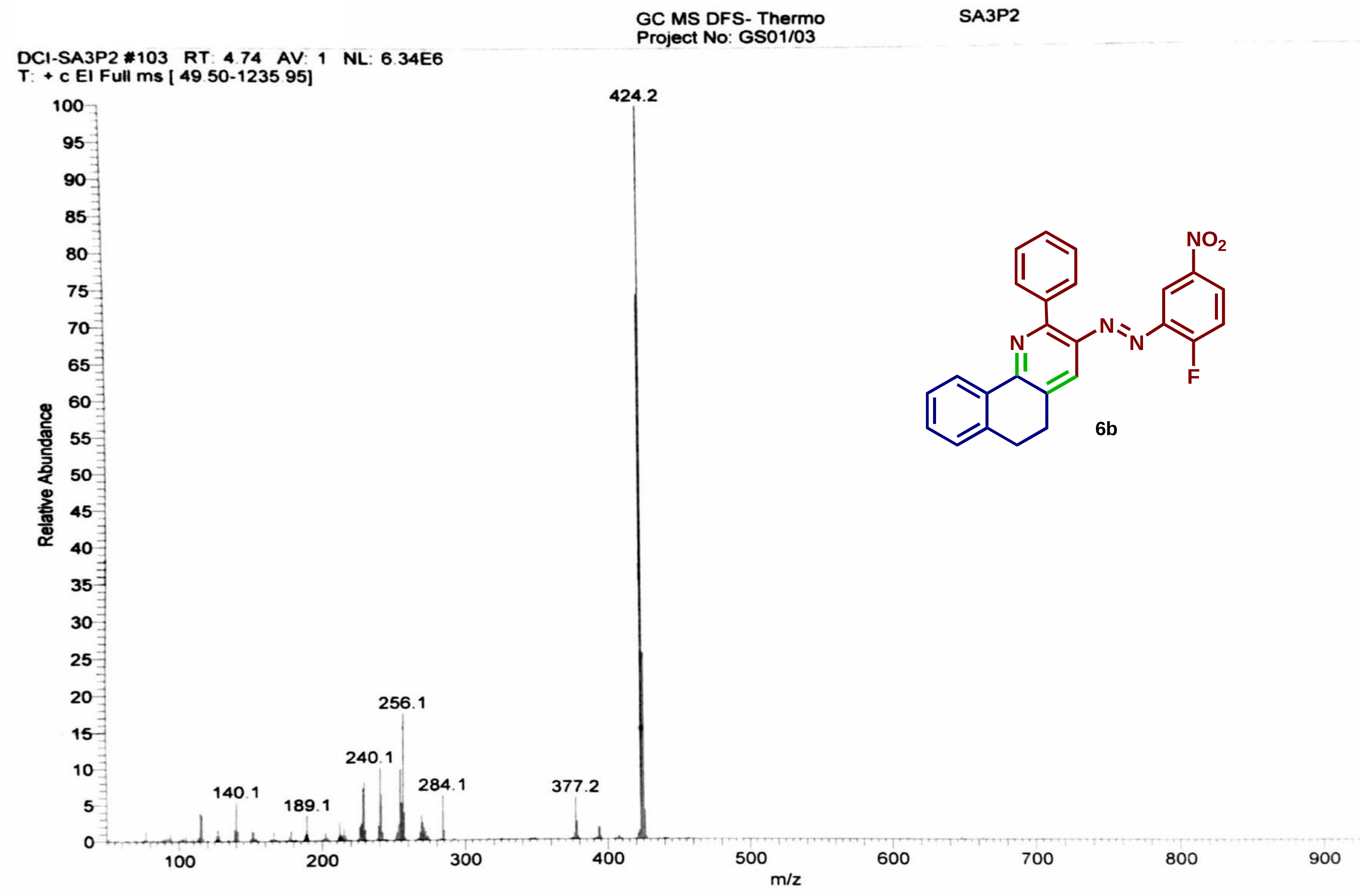

Figure S32. HRMS Spectra for compound **6b**.

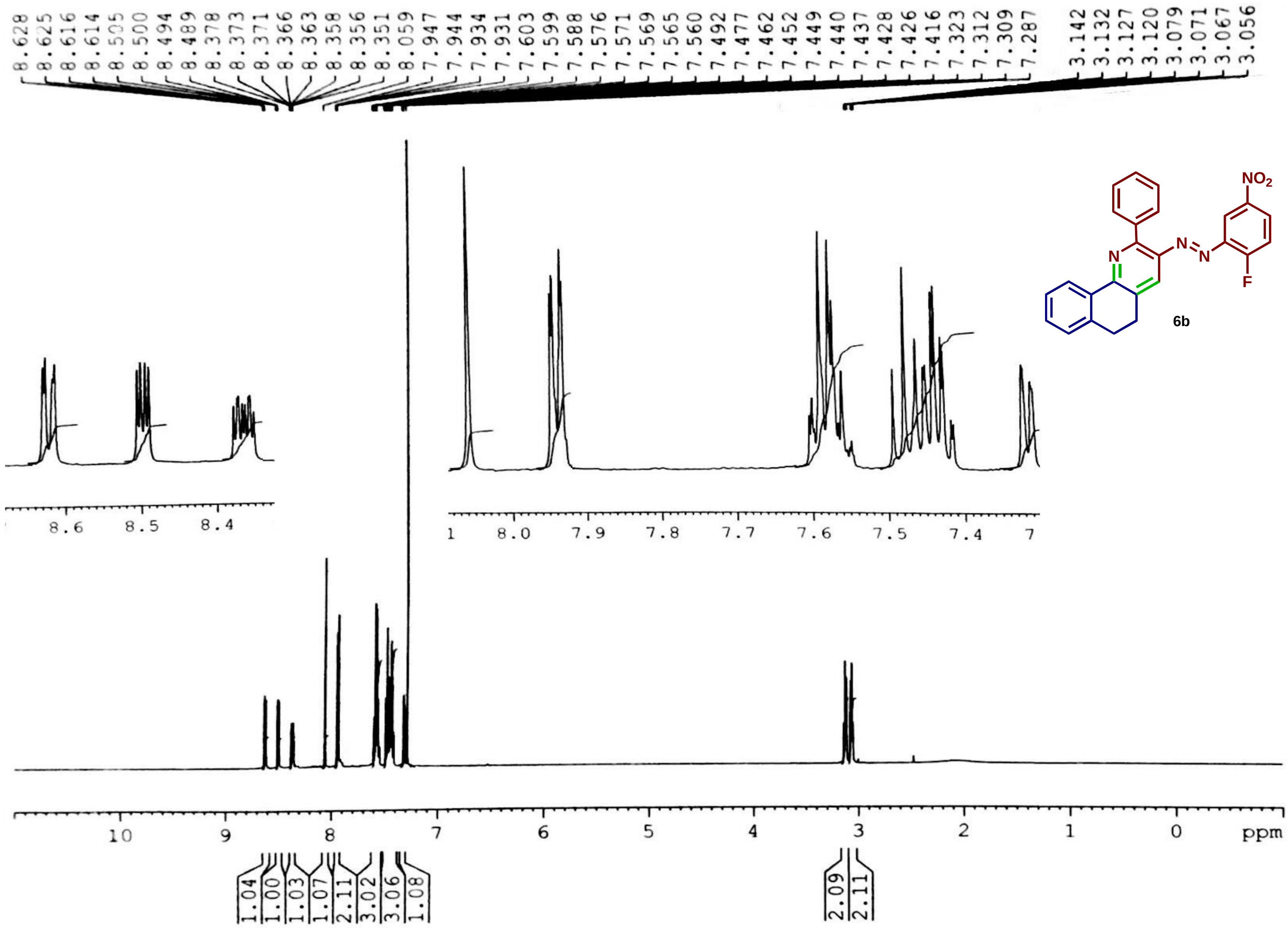

Figure S33. ^{1}H NMR Spectra ($CDCl_3$, 400 MHz) for compound **6b**.

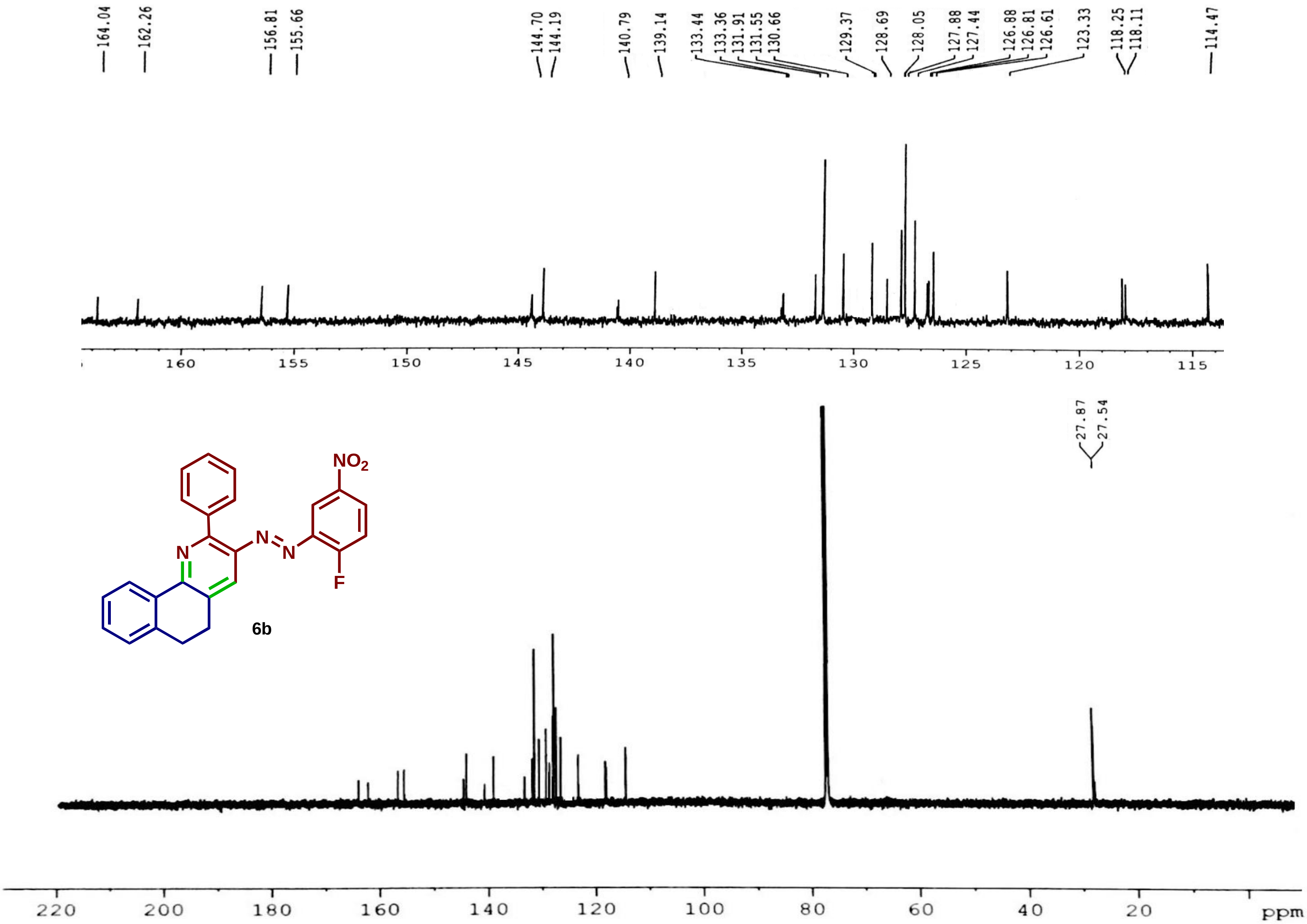

Figure S34. ^{13}C NMR Spectra ($CDCl_3$, 150 MHz) for compound **6b**.

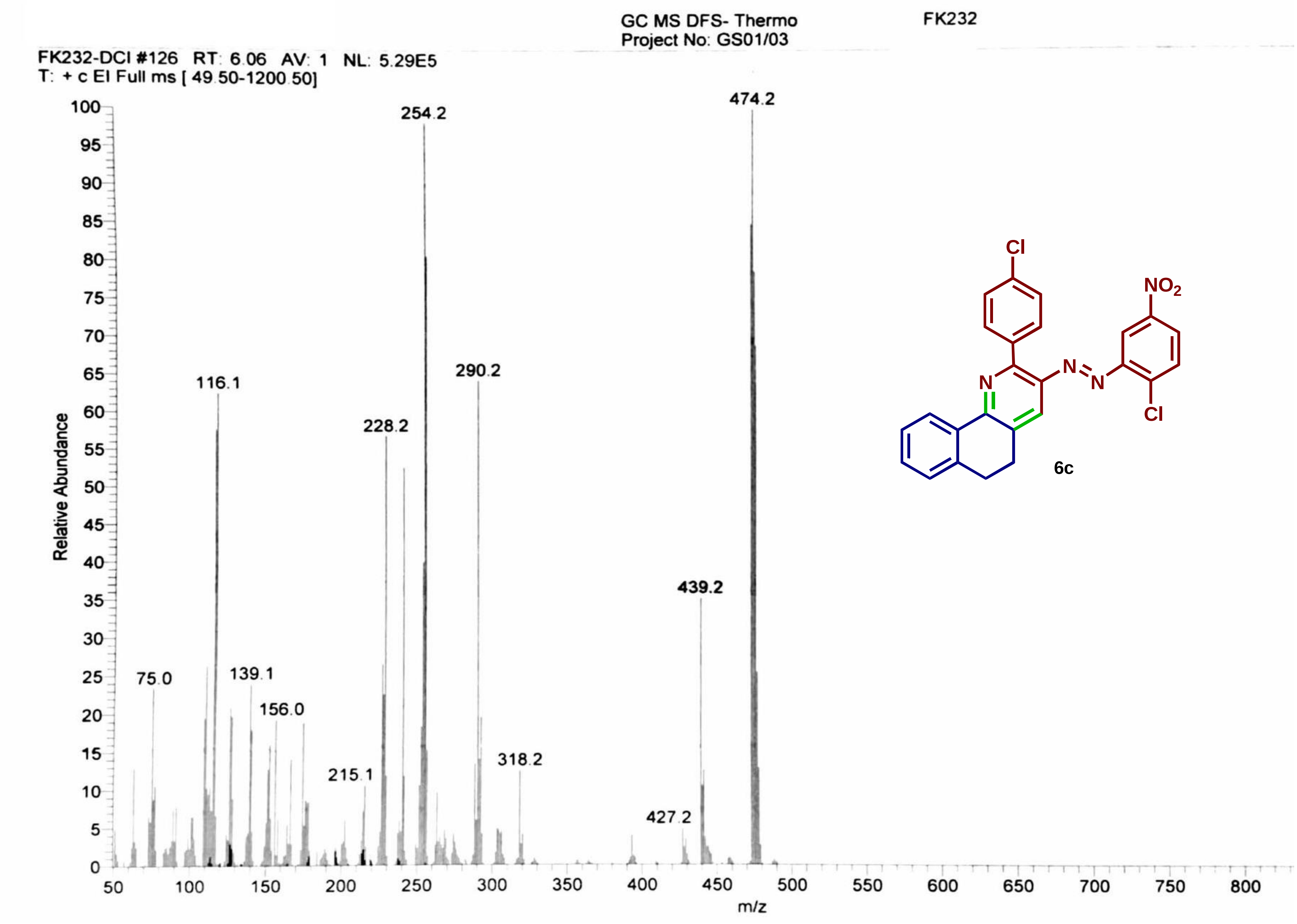

Figure S35. Mass Spectra for compound **6c**.

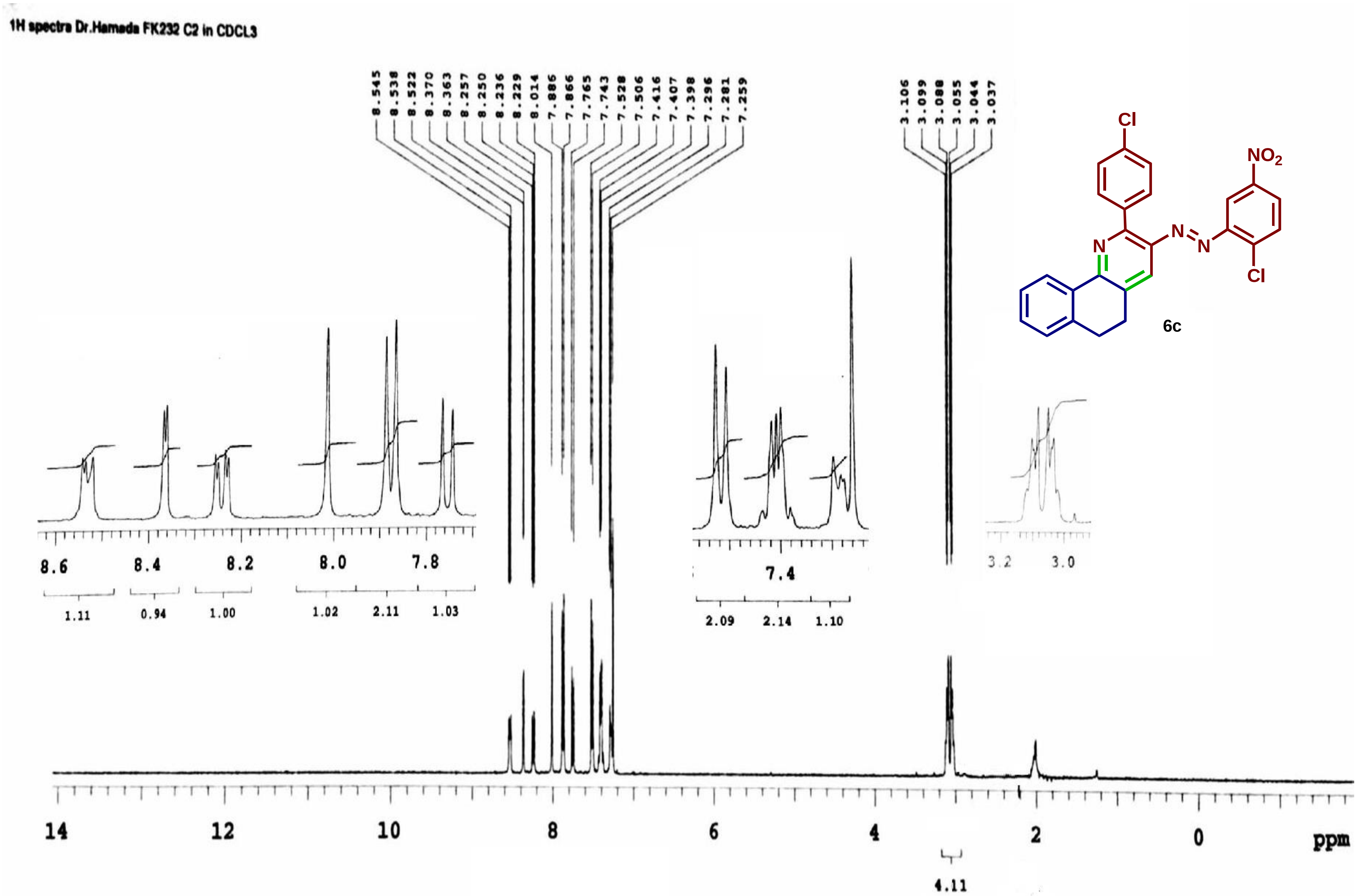

Figure S36. ^{1}H NMR Spectra ($CDCl_3$, 400 MHz) for compound **6c**.

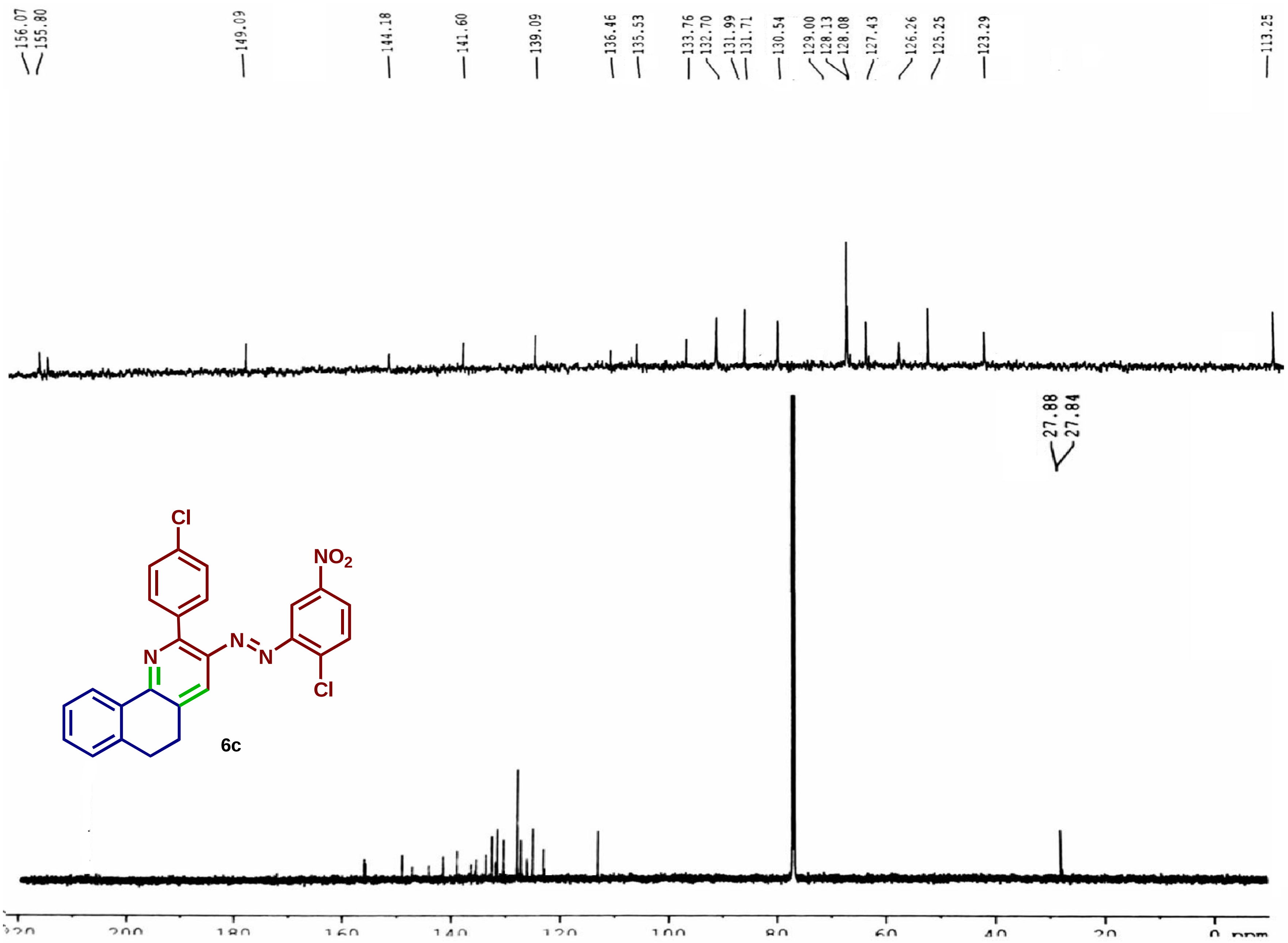

Figure S37. ^{13}C NMR Spectra ($CDCl_3$, 150 MHz) for compound **6c**.

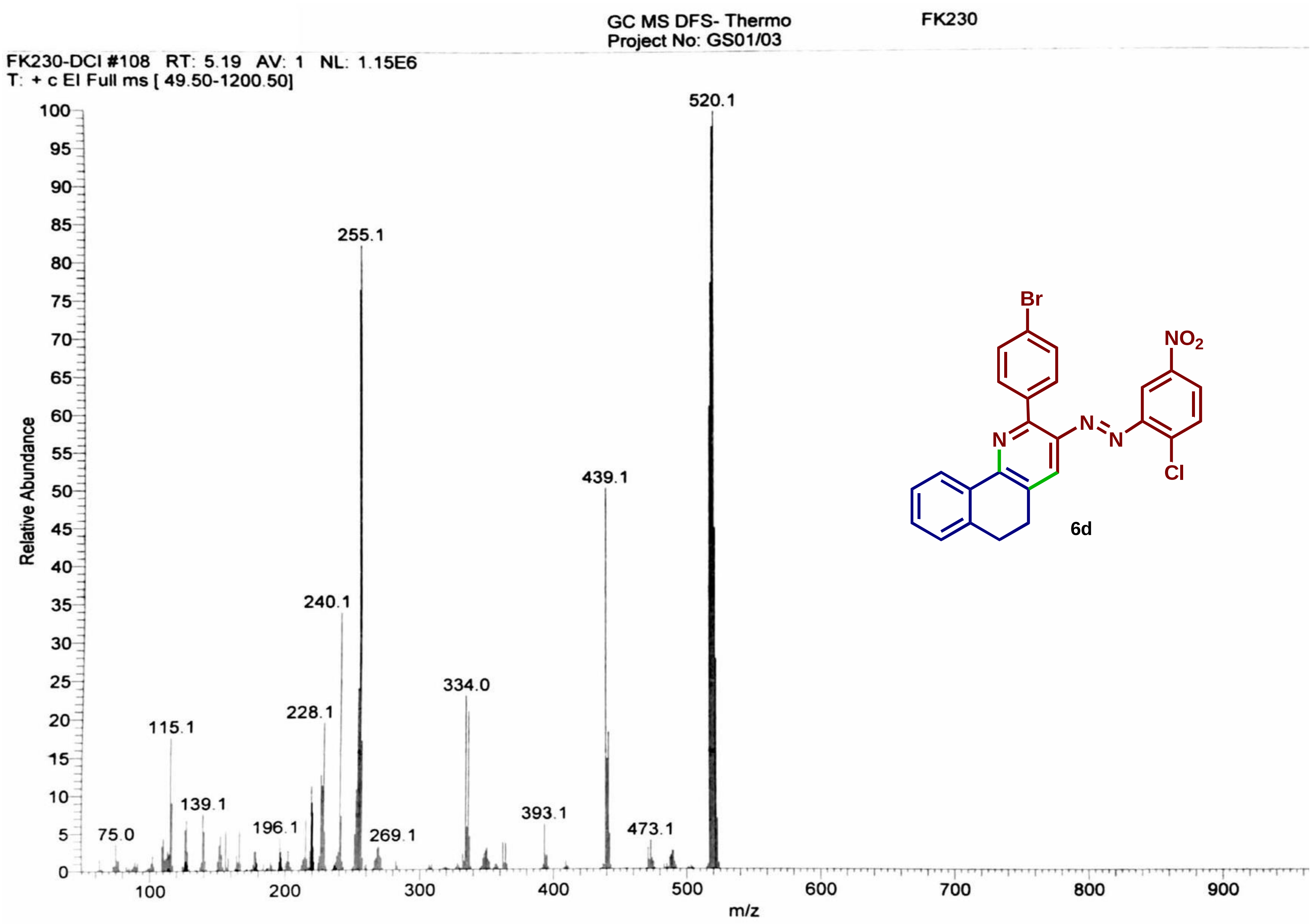

Figure S38. Mass Spectra for compound **6d**.

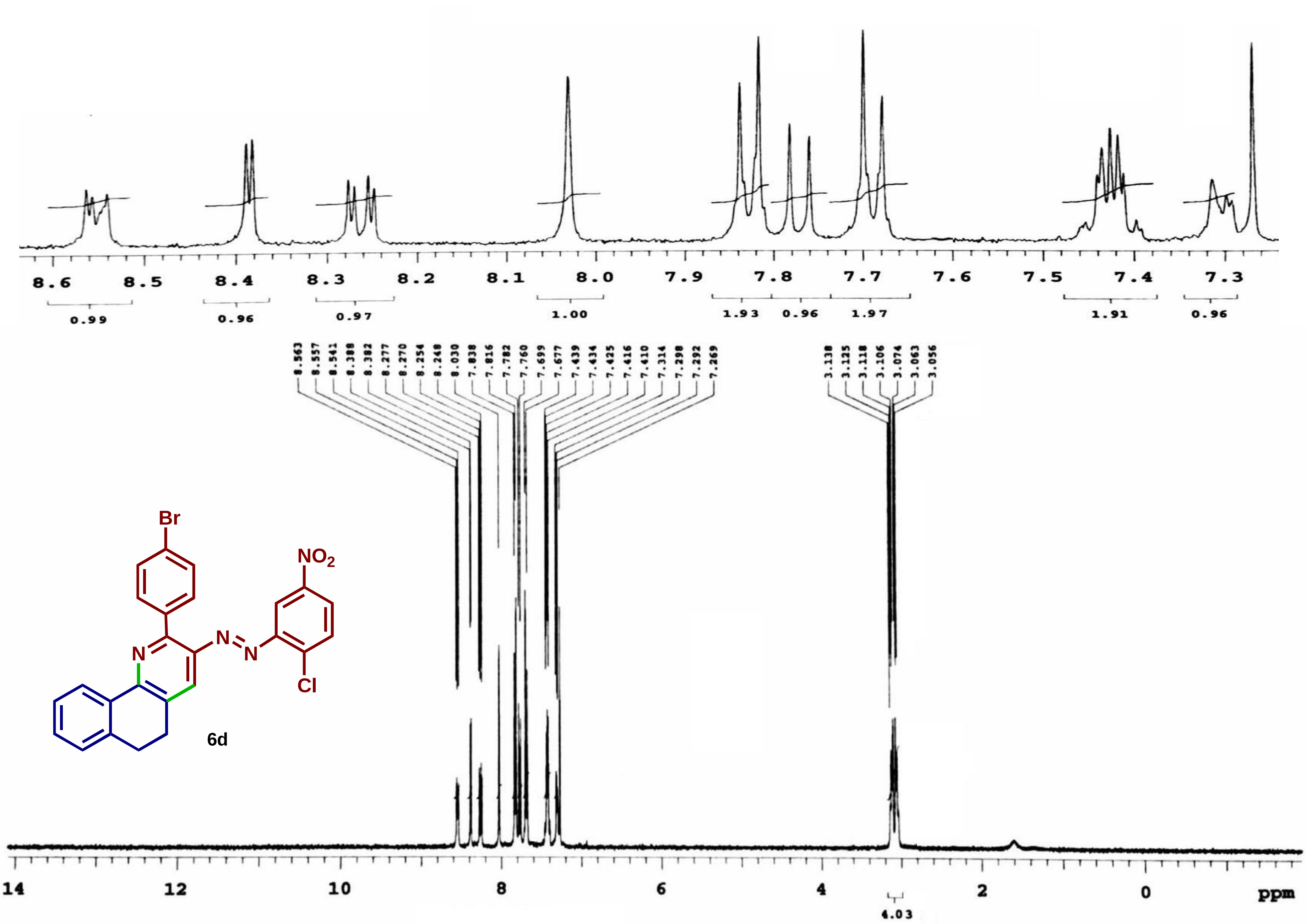

Figure S39. ^{1}H NMR Spectra ($CDCl_3$, 400 MHz) for compound **6d**.

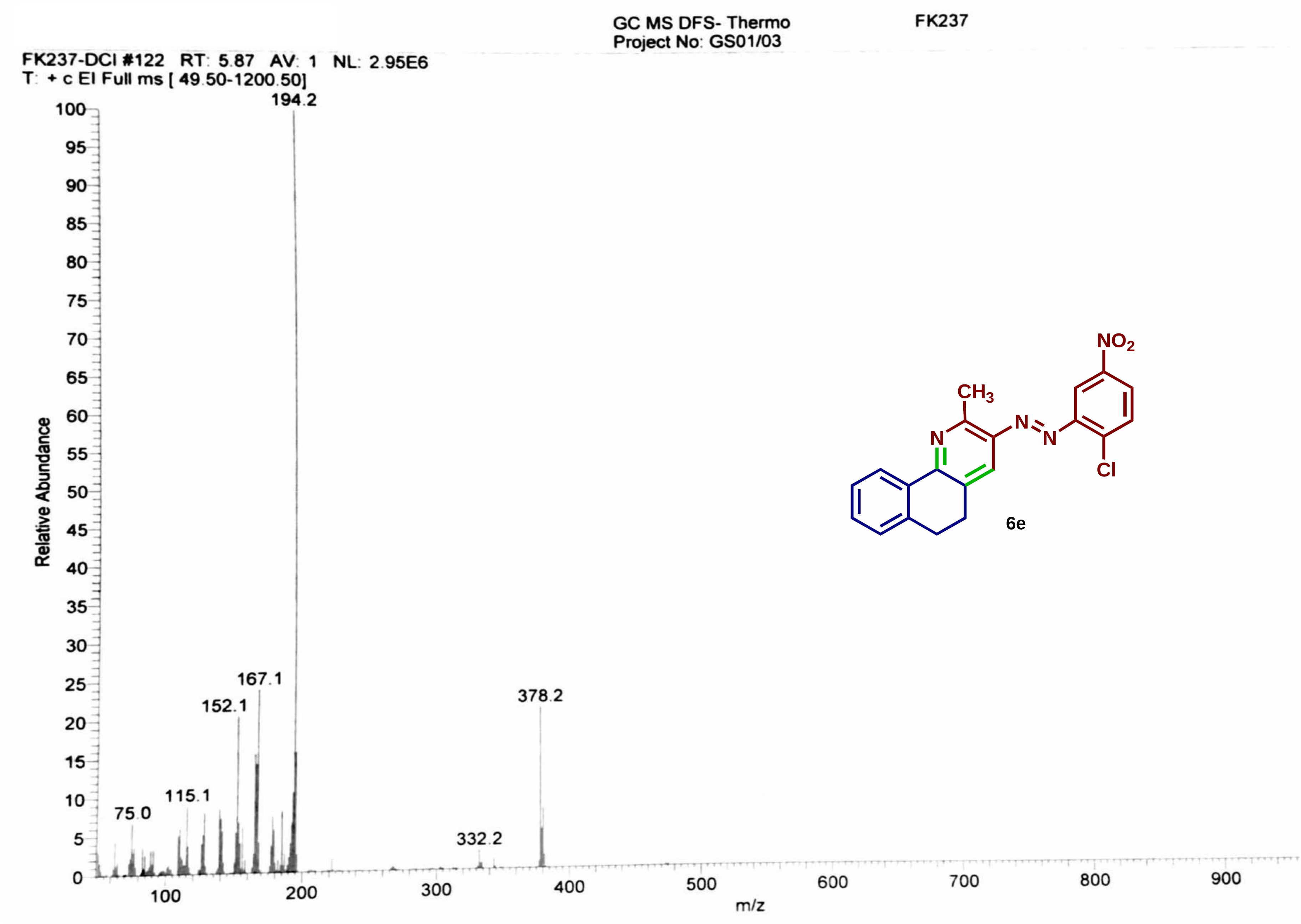

Figure S40. Mass Spectra for compound **6e.**

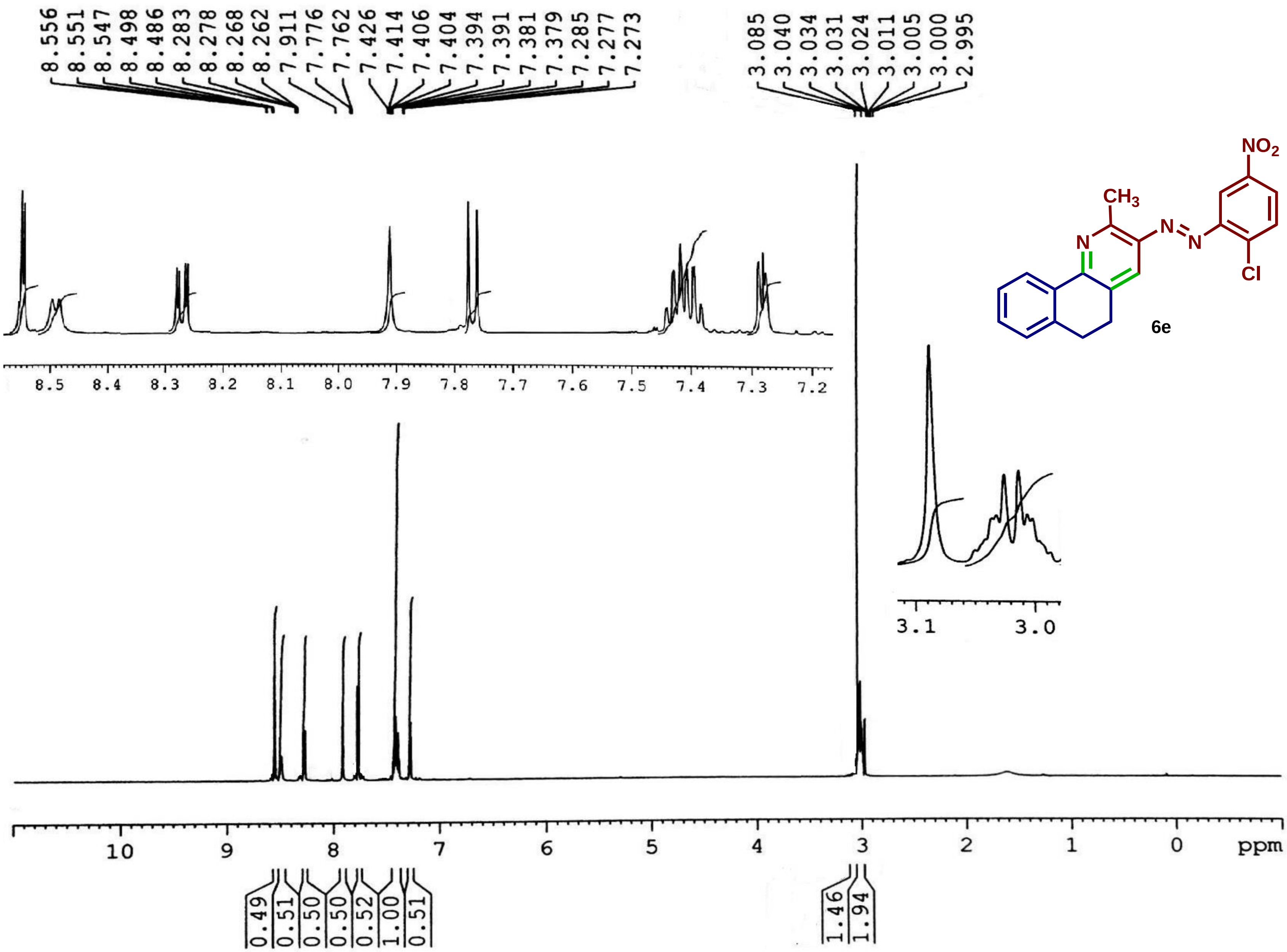

Figure S41. ^{1}H NMR Spectra ($CDCl_3$, 400 MHz) for compound **6e**.

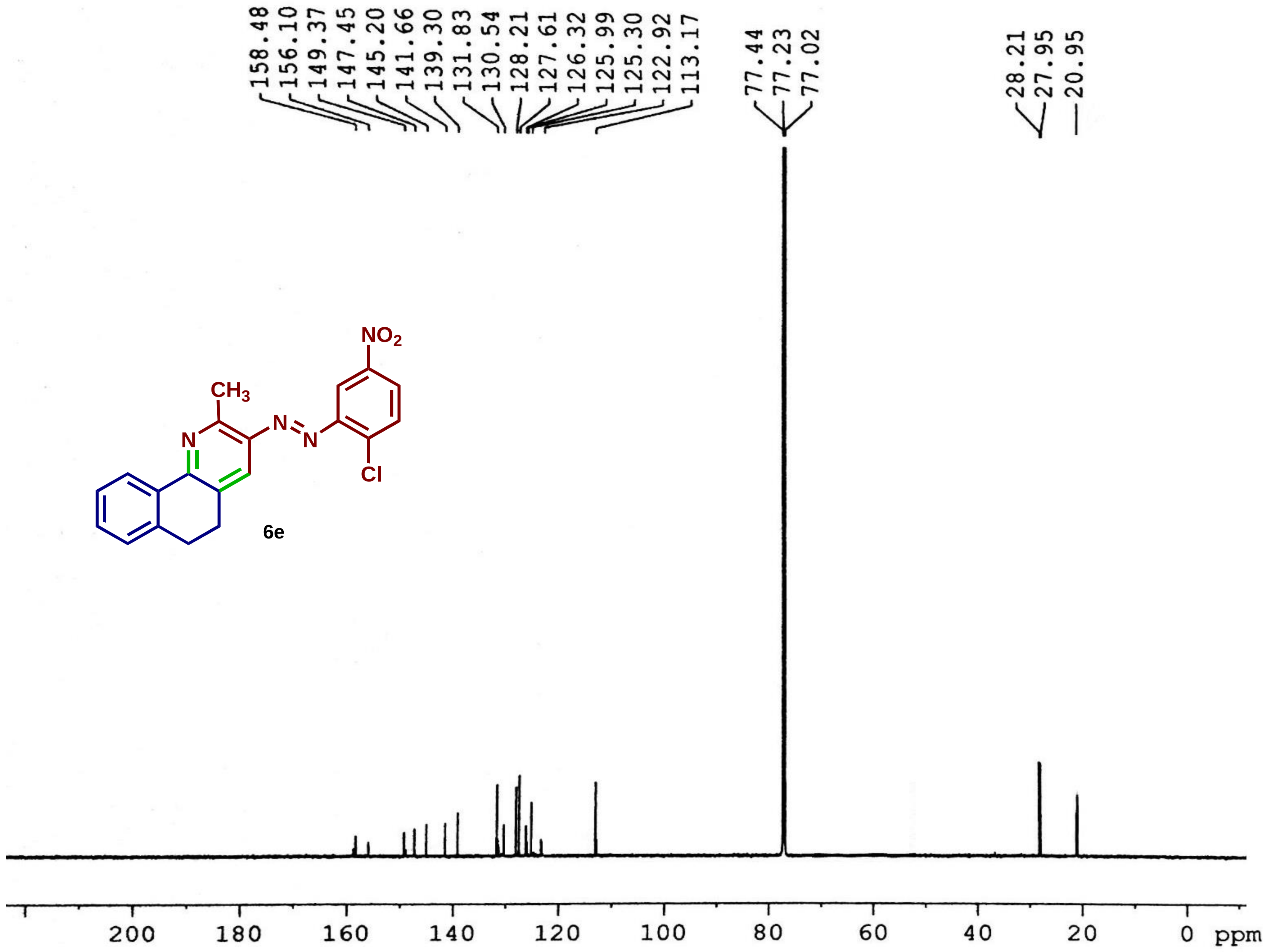

Figure S42. ^{13}C NMR Spectra ($CDCl_3$, 150 MHz) for compound **6e**.
