# Supplementary material for: High pressure assisted synthetic approach for novel 6,7-dihydro-5H-benzo[6,7]cyclohepta[1,2-b]pyridine and 5,6-dihydrobenzo[h]quinoline derivatives and their assessment as anticancer agents
Source: Sci Rep. 2020 Dec 10;10:21691. doi: 10.1038/s41598-020-78590-x (PMC7728779; doi:10.1038/s41598-020-78590-x)

checkCIF/PLATON report

Structure factors have been supplied for datablock(s) I

THIS REPORT IS FOR GUIDANCE ONLY. IF USED AS PART OF A REVIEW PROCEDURE FOR PUBLICATION, IT SHOULD NOT REPLACE THE EXPERTISE OF AN EXPERIENCED CRYSTALLOGRAPHIC REFEREE.

No syntax errors found. CIF dictionary Interpreting this report

Datablock: I

Bond precision: C-C = 0.0035 A Wavelength=1.54178

Cell:	a=14.9070(3)	b=9.3911(2)	c=15.6885(3)
	alpha=90	beta=101.620(1)	gamma=90
Temperature:	296 K		

	Calculated	Reported
Volume	2151.27(8)	2151.27(8)
Space group	P 21/c	P 1 21/c 1
Hall group	-P 2ybc	-P 2ybc
Moiety formula	C26 H19 F N4 O2	?
Sum formula	C26 H19 F N4 O2	C26 H19 F N4 O2
Mr	438.45	438.45
Dx,g cm-3	1.354	1.354
Z	4	4
Mu (mm-1)	0.771	0.771
F000	912.0	912.0
F000’	914.89	
h,k,lmax	17,11,18	17,11,18
Nref	3800	3755
Tmin,Tmax	0.887,0.955	0.800,0.950
Tmin’	0.857	

Correction method= # Reported T Limits: Tmin=0.800 Tmax=0.950
AbsCorr = MULTI-SCAN

Data completeness= 0.988 Theta(max)= 66.600

R(reflections)= 0.0526(2826) wR2(reflections)= 0.1761(3755)

S = 1.020 Npar= 298

The following ALERTS were generated. Each ALERT has the format
test-name_ALERT_alert-type_alert-level.
Click on the hyperlinks for more details of the test.

Alert level B

PLAT934_ALERT_3_B Number of (Iobs-Icalc)/Sigma(W) > 10 Outliers .. 2 Check

Alert level C

PLAT242_ALERT_2_C Low 'MainMol' Ueq as Compared to Neighbors of N4 Check
PLAT906_ALERT_3_C Large K Value in the Analysis of Variance ...... 10.542 Check
PLAT911_ALERT_3_C Missing FCF Refl Between Thmin & STh/L= 0.595 46 Report
PLAT918_ALERT_3_C Reflection(s) with I(obs) much Smaller I(calc) . 2 Check
PLAT939_ALERT_3_C Large Value of Not (SHELXL) Weight Optimized S . 33.54 Check

Alert level G

PLAT005_ALERT_5_G No Embedded Refinement Details Found in the CIF Please Do !
PLAT072_ALERT_2_G SHELXL First Parameter in WGHT Unusually Large 0.11 Report
PLAT093_ALERT_1_G No s.u.'s on H-positions, Refinement Reported as mixed Check
PLAT909_ALERT_3_G Percentage of I>2sig(I) Data at Theta(Max) Still 47% Note
PLAT978_ALERT_2_G Number C-C Bonds with Positive Residual Density. 0 Info

0 **ALERT level A** = Most likely a serious problem - resolve or explain
1 **ALERT level B** = A potentially serious problem, consider carefully
5 **ALERT level C** = Check. Ensure it is not caused by an omission or oversight
5 **ALERT level G** = General information/check it is not something unexpected

1 ALERT type 1 CIF construction/syntax error, inconsistent or missing data
3 ALERT type 2 Indicator that the structure model may be wrong or deficient
6 ALERT type 3 Indicator that the structure quality may be low
0 ALERT type 4 Improvement, methodology, query or suggestion
1 ALERT type 5 Informative message, check

It is advisable to attempt to resolve as many as possible of the alerts in all categories. Often the minor alerts point to easily fixed oversights, errors and omissions in your CIF or refinement strategy, so attention to these fine details can be worthwhile. In order to resolve some of the more serious problems it may be necessary to carry out additional measurements or structure refinements. However, the purpose of your study may justify the reported deviations and the more serious of these should normally be commented upon in the discussion or experimental section of a paper or in the "special_details" fields of the CIF. checkCIF was carefully designed to identify outliers and unusual parameters, but every test has its limitations and alerts that are not important in a particular case may appear. Conversely, the absence of alerts does not guarantee there are no aspects of the results needing attention. It is up to the individual to critically assess their own results and, if necessary, seek expert advice.

Publication of your CIF in IUCr journals

A basic structural check has been run on your CIF. These basic checks will be run on all CIFs submitted for publication in IUCr journals (*Acta Crystallographica*, *Journal of Applied Crystallography*, *Journal of Synchrotron Radiation*); however, if you intend to submit to *Acta Crystallographica Section C* or *E* or *IUCrData*, you should make sure that full publication checks are run on the final version of your CIF prior to submission.

Publication of your CIF in other journals

Please refer to the *Notes for Authors* of the relevant journal for any special instructions relating to CIF submission.

PLATON version of 04/06/2020; check.def file version of 02/06/2020

Datablock I - ellipsoid plot

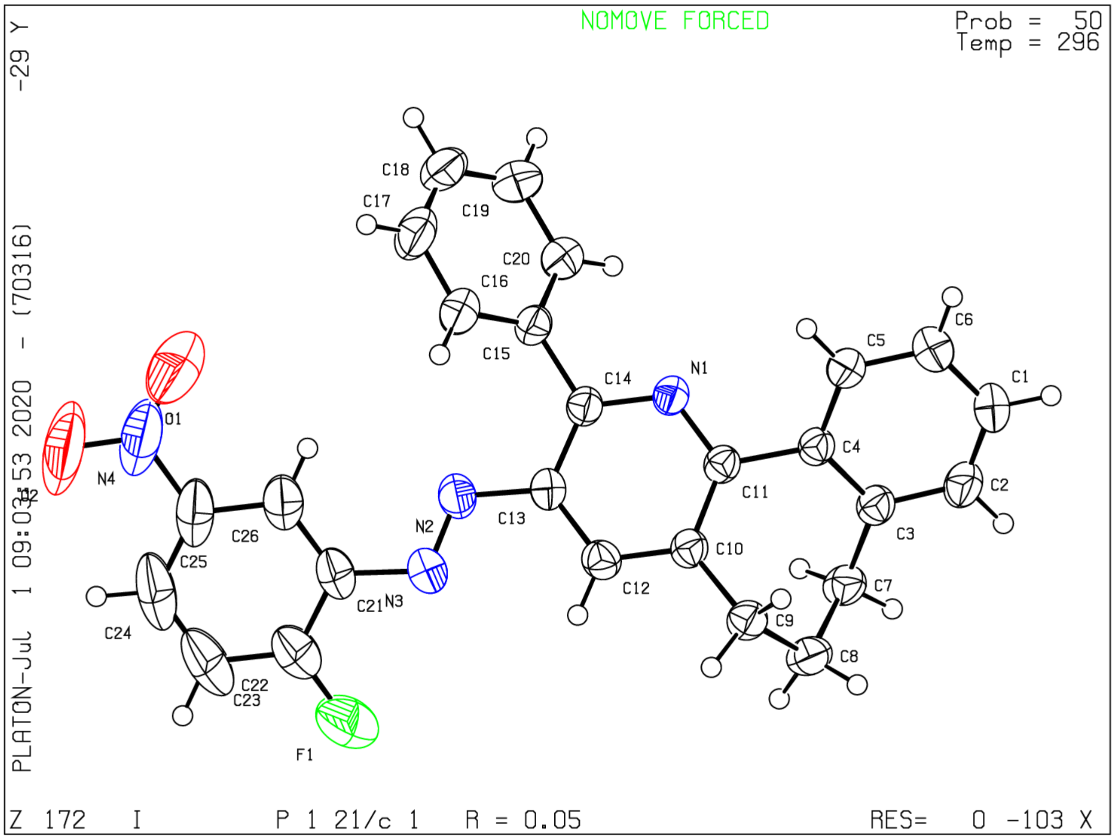

Supplement: Supplementary file 3 — Supplementary Information 2. [file 41598_2020_78590_MOESM3_ESM.pdf]
