# Supplementary material for: High pressure assisted synthetic approach for novel 6,7-dihydro-5H-benzo[6,7]cyclohepta[1,2-b]pyridine and 5,6-dihydrobenzo[h]quinoline derivatives and their assessment as anticancer agents
Source: Sci Rep. 2020 Dec 10;10:21691. doi: 10.1038/s41598-020-78590-x (PMC7728779; doi:10.1038/s41598-020-78590-x)

checkCIF/PLATON report

Structure factors have been supplied for datablock(s) User-defined

THIS REPORT IS FOR GUIDANCE ONLY. IF USED AS PART OF A REVIEW PROCEDURE FOR PUBLICATION, IT SHOULD NOT REPLACE THE EXPERTISE OF AN EXPERIENCED CRYSTALLOGRAPHIC REFEREE.

No syntax errors found. CIF dictionary Interpreting this report

Datablock: User-defined

```
Bond precision:  C-C = 0.0077 A          Wavelength=0.71075

Cell:           a=9.058(2)          b=10.791(3)      c=13.031(3)
                alpha=103.776(8)    beta=94.977(7)   gamma=108.471(8)
Temperature:    293 K

                 Calculated                 Reported
Volume           1155.0(5)                  1154.9(5)
Space group      P -1                       P -1
Hall group       -P 1                       -P 1
Moiety formula   C26 H18 Br Cl N4 O2        C26 H18 Br Cl N4 O2
Sum formula      C26 H18 Br Cl N4 O2        C26 H18 Br Cl N4 O2
Mr               533.79                     533.81
Dx,g cm-3        1.535                      1.535
Z                2                          2
Mu (mm-1)        1.928                      1.933
F000             540.0                      540.0
F000'            539.90
h,k,lmax         10,12,15                   10,12,15
Nref             4085                       4061
Tmin,Tmax        0.775,0.908                0.552,0.908
Tmin'            0.748

Correction method= # Reported T Limits: Tmin=0.552 Tmax=0.908
AbsCorr = MULTI-SCAN

Data completeness= 0.994          Theta(max)= 25.020

R(reflections)= 0.0501( 1929)     wR2(reflections)= 0.1387( 4061)

S = 0.968                 Npar= 307
```

The following ALERTS were generated. Each ALERT has the format
test-name_ALERT_alert-type_alert-level.
Click on the hyperlinks for more details of the test.

Alert level C

```
PLAT026_ALERT_3_C Ratio Observed / Unique Reflections (too) Low ..        48% Check
PLAT341_ALERT_3_C Low Bond Precision on  C-C Bonds ...............    0.00771 Ang.
PLAT906_ALERT_3_C Large K Value in the Analysis of Variance ......      7.695 Check
PLAT906_ALERT_3_C Large K Value in the Analysis of Variance ......      3.027 Check
PLAT910_ALERT_3_C Missing # of FCF Reflection(s) Below Theta(Min).          8 Note
PLAT911_ALERT_3_C Missing FCF Refl Between Thmin & STh/L=    0.595         13 Report
```

Alert level G

```
CHEMS02_ALERT_1_G  Please check that you have entered the correct
            _publ_requested_category classification of your compound;
            FI or CI or EI for inorganic; FM or CM or EM for metal-organic;
            FO or CO or EO for organic.
            From the CIF: _publ_requested_category   CHOOSE FI FM FO CI CM CO or A
            From the CIF: _chemical_formula_sum :C26 H18 Br1 Cl1 N4 O2
PLAT005_ALERT_5_G No Embedded Refinement Details Found  in the CIF     Please Do !
PLAT199_ALERT_1_G Reported _cell_measurement_temperature ..... (K)        293 Check
PLAT200_ALERT_1_G Reported   _diffrn_ambient_temperature ..... (K)        293 Check
PLAT434_ALERT_2_G Short Inter HL..HL Contact Br1      ..Br1              3.35 Ang.
                                                 1-x,2-y,-z  =      2_675 Check
PLAT882_ALERT_1_G No Datum for _diffrn_reflns_av_unetI/netI ......     Please Do !
PLAT978_ALERT_2_G Number C-C Bonds with Positive Residual Density.          1 Info
```

0 **ALERT level A** = Most likely a serious problem - resolve or explain
0 **ALERT level B** = A potentially serious problem, consider carefully
6 **ALERT level C** = Check. Ensure it is not caused by an omission or oversight
7 **ALERT level G** = General information/check it is not something unexpected

4 ALERT type 1 CIF construction/syntax error, inconsistent or missing data
2 ALERT type 2 Indicator that the structure model may be wrong or deficient
6 ALERT type 3 Indicator that the structure quality may be low
0 ALERT type 4 Improvement, methodology, query or suggestion
1 ALERT type 5 Informative message, check

It is advisable to attempt to resolve as many as possible of the alerts in all categories. Often the minor alerts point to easily fixed oversights, errors and omissions in your CIF or refinement strategy, so attention to these fine details can be worthwhile. In order to resolve some of the more serious problems it may be necessary to carry out additional measurements or structure refinements. However, the purpose of your study may justify the reported deviations and the more serious of these should normally be commented upon in the discussion or experimental section of a paper or in the "special_details" fields of the CIF. checkCIF was carefully designed to identify outliers and unusual parameters, but every test has its limitations and alerts that are not important in a particular case may appear. Conversely, the absence of alerts does not guarantee there are no aspects of the results needing attention. It is up to the individual to critically assess their own results and, if necessary, seek expert advice.

Publication of your CIF in IUCr journals

A basic structural check has been run on your CIF. These basic checks will be run on all CIFs submitted for publication in IUCr journals (*Acta Crystallographica*, *Journal of Applied Crystallography*, *Journal of Synchrotron Radiation*); however, if you intend to submit to *Acta Crystallographica Section C* or *E* or *IUCrData*, you should make sure that full publication checks are run on the final version of your CIF prior to submission.

Publication of your CIF in other journals

Please refer to the *Notes for Authors* of the relevant journal for any special instructions relating to CIF submission.

PLATON version of 04/06/2020; check.def file version of 02/06/2020

Datablock User-defined - ellipsoid plot

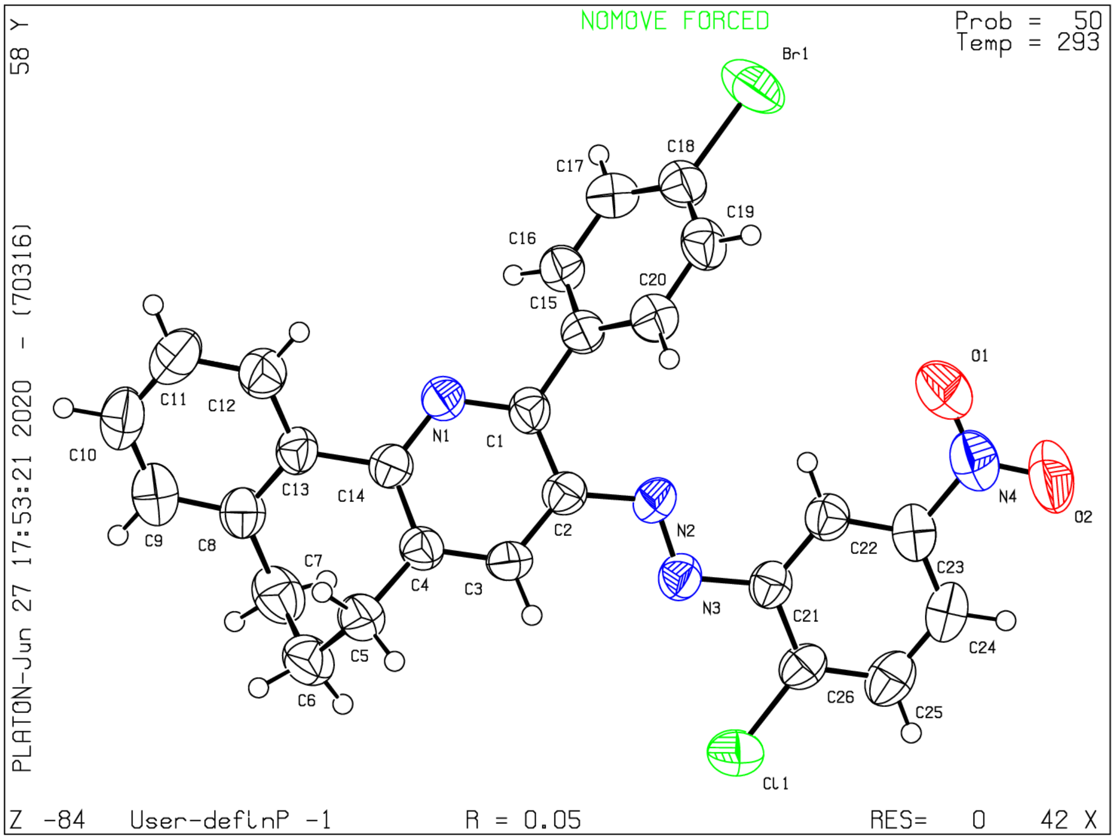

Supplement: Supplementary file 4 — Supplementary Information 3. [file 41598_2020_78590_MOESM4_ESM.pdf]
