# Supplementary material for: High pressure assisted synthetic approach for novel 6,7-dihydro-5H-benzo[6,7]cyclohepta[1,2-b]pyridine and 5,6-dihydrobenzo[h]quinoline derivatives and their assessment as anticancer agents
Source: Sci Rep. 2020 Dec 10;10:21691. doi: 10.1038/s41598-020-78590-x (PMC7728779; doi:10.1038/s41598-020-78590-x)

checkCIF/PLATON report

Structure factors have been supplied for datablock(s) User-defined

THIS REPORT IS FOR GUIDANCE ONLY. IF USED AS PART OF A REVIEW PROCEDURE FOR PUBLICATION, IT SHOULD NOT REPLACE THE EXPERTISE OF AN EXPERIENCED CRYSTALLOGRAPHIC REFEREE.

No syntax errors found.   CIF dictionary   Interpreting this report

Datablock: User-defined

```
Bond precision:  C-C = 0.0081 A          Wavelength=0.71075

Cell:           a=9.3531(12)    b=14.0117(16)     c=15.5851(19)
                alpha=90        beta=94.265(7)    gamma=90
Temperature:    293 K

                Calculated                Reported
Volume          2036.8(4)                 2036.8(5)
Space group     P 21/c                    P 1 21/c 1
Hall group      -P 2ybc                   -P 2ybc
Moiety formula  C25 H17 F N4 O2           C25 H17 F N4 O2
Sum formula     C25 H17 F N4 O2           C25 H17 F N4 O2
Mr              424.43                    424.43
Dx,g cm-3       1.384                     1.384
Z               4                         4
Mu (mm-1)       0.097                     0.097
F000            880.0                     880.0
F000'           880.40
h,k,lmax        11,16,18                  11,16,18
Nref            3601                      3578
Tmin,Tmax       0.991,0.995               0.256,0.995
Tmin'           0.981

Correction method= # Reported T Limits: Tmin=0.256 Tmax=0.995
AbsCorr = MULTI-SCAN

Data completeness= 0.994          Theta(max)= 25.030

R(reflections)= 0.0676( 1315)     wR2(reflections)= 0.2254( 3578)

S = 0.933                 Npar= 289
```

The following ALERTS were generated. Each ALERT has the format
test-name_ALERT_alert-type_alert-level.
Click on the hyperlinks for more details of the test.

Alert level B

```
PLAT026_ALERT_3_B Ratio Observed / Unique Reflections (too) Low ..        37% Check
```

Alert level C

```
PLAT241_ALERT_2_C High   'MainMol' Ueq as Compared to Neighbors of         C5 Check
PLAT242_ALERT_2_C Low    'MainMol' Ueq as Compared to Neighbors of         N4 Check
PLAT242_ALERT_2_C Low    'MainMol' Ueq as Compared to Neighbors of         C6 Check
PLAT334_ALERT_2_C Small Aver. Benzene C-C Dist C20      -C25             1.37 Ang.
PLAT340_ALERT_3_C Low Bond Precision on  C-C Bonds ...............    0.00815 Ang.
PLAT360_ALERT_2_C Short  C(sp3)-C(sp3) Bond  C4       - C5       .       1.39 Ang.
```

Alert level G

```
CHEMS02_ALERT_1_G  Please check that you have entered the correct
            _publ_requested_category classification of your compound;
            FI or CI or EI for inorganic; FM or CM or EM for metal-organic;
            FO or CO or EO for organic.
            From the CIF: _publ_requested_category   CHOOSE FI FM FO CI CM CO or A
            From the CIF: _chemical_formula_sum :C25 H17 F1 N4 O2
PLAT005_ALERT_5_G No Embedded Refinement Details Found  in the CIF     Please Do !
PLAT072_ALERT_2_G SHELXL First  Parameter in WGHT  Unusually Large       0.10 Report
PLAT199_ALERT_1_G Reported _cell_measurement_temperature ..... (K)        293 Check
PLAT200_ALERT_1_G Reported   _diffrn_ambient_temperature ..... (K)        293 Check
PLAT860_ALERT_3_G Number of Least-Squares Restraints .............         16 Note
PLAT882_ALERT_1_G No Datum for _diffrn_reflns_av_unetI/netI ......     Please Do !
```

```
   0 ALERT level A = Most likely a serious problem - resolve or explain
   1 ALERT level B = A potentially serious problem, consider carefully
   6 ALERT level C = Check. Ensure it is not caused by an omission or oversight
   7 ALERT level G = General information/check it is not something unexpected

   4 ALERT type 1 CIF construction/syntax error, inconsistent or missing data
   6 ALERT type 2 Indicator that the structure model may be wrong or deficient
   3 ALERT type 3 Indicator that the structure quality may be low
   0 ALERT type 4 Improvement, methodology, query or suggestion
   1 ALERT type 5 Informative message, check
```

It is advisable to attempt to resolve as many as possible of the alerts in all categories. Often the minor alerts point to easily fixed oversights, errors and omissions in your CIF or refinement strategy, so attention to these fine details can be worthwhile. In order to resolve some of the more serious problems it may be necessary to carry out additional measurements or structure refinements. However, the purpose of your study may justify the reported deviations and the more serious of these should normally be commented upon in the discussion or experimental section of a paper or in the "special_details" fields of the CIF. checkCIF was carefully designed to identify outliers and unusual parameters, but every test has its limitations and alerts that are not important in a particular case may appear. Conversely, the absence of alerts does not guarantee there are no aspects of the results needing attention. It is up to the individual to critically assess their own results and, if necessary, seek expert advice.

Publication of your CIF in IUCr journals

A basic structural check has been run on your CIF. These basic checks will be run on all CIFs submitted for publication in IUCr journals (*Acta Crystallographica*, *Journal of Applied Crystallography*, *Journal of Synchrotron Radiation*); however, if you intend to submit to *Acta Crystallographica Section C* or *E* or *IUCrData*, you should make sure that full publication checks are run on the final version of your CIF prior to submission.

Publication of your CIF in other journals

Please refer to the *Notes for Authors* of the relevant journal for any special instructions relating to CIF submission.

PLATON version of 04/06/2020; check.def file version of 02/06/2020

Datablock User-defined - ellipsoid plot

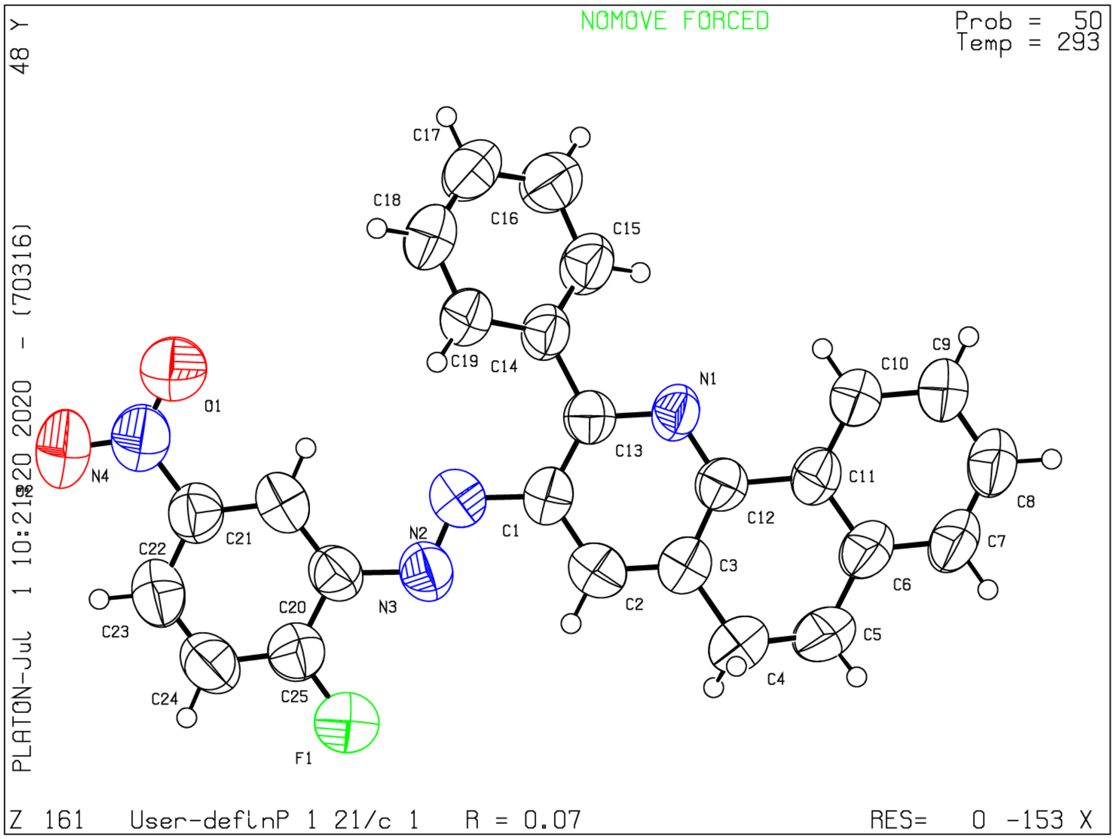

Supplement: Supplementary file 5 — Supplementary Information 4. [file 41598_2020_78590_MOESM5_ESM.pdf]
